# Analyzing bioactive effects of the minor hop compound xanthohumol C on human breast cancer cells using quantitative proteomics

**DOI:** 10.1371/journal.pone.0213469

**Published:** 2019-03-15

**Authors:** Simon Roehrer, Verena Stork, Christina Ludwig, Mirjana Minceva, Jürgen Behr

**Affiliations:** 1 Biothermodynamics, TUM School of Life and Food Sciences Weihenstephan, Technical University of Munich, Freising, Germany; 2 Bavarian Center for Biomolecular Mass Spectrometry, TUM School of Life and Food Sciences Weihenstephan, Technical University of Munich, Freising, Germany; Centre for Cellular and Molecular Biology, INDIA

## Abstract

Minor prenylated hop compounds have been attracting increasing attention due to their promising anticarcinogenic properties. Even after intensive purification from natural raw extracts, allocating certain activities to single compounds or complex interactions of the main compound with remaining impurities in very low concentration is difficult. In this study, dose-dependent antiproliferative and cytotoxic effects of the promising xanthohumol (XN) analogue xanthohumol C (XNC) were evaluated and compared to XN and a XN-enriched hop extract (XF). It was demonstrated that the cell growth inhibition of human breast cancer cell line (MCF-7) significantly increases after being treated with XNC compared to XN and XF. Based on label-free data-dependent acquisition proteomics, physiological influences on the proteome of MCF-7 cells were analyzed. Different modes of action between XNC and XN treated MCF-7 cells could be postulated. XNC causes ER stress and seems to be involved in cell-cell adhesion, whereas XN influences cell cycles and DNA replication as well as type I interferon signaling pathway. The results demonstrate the utility of using quantitative proteomics for bioactivity screenings of minor hop compounds and underscore the importance of isolating highly pure compounds into their distinct forms to analyze their different and possibly synergistic activities and modes of action.

## Introduction

**Fig 1:**
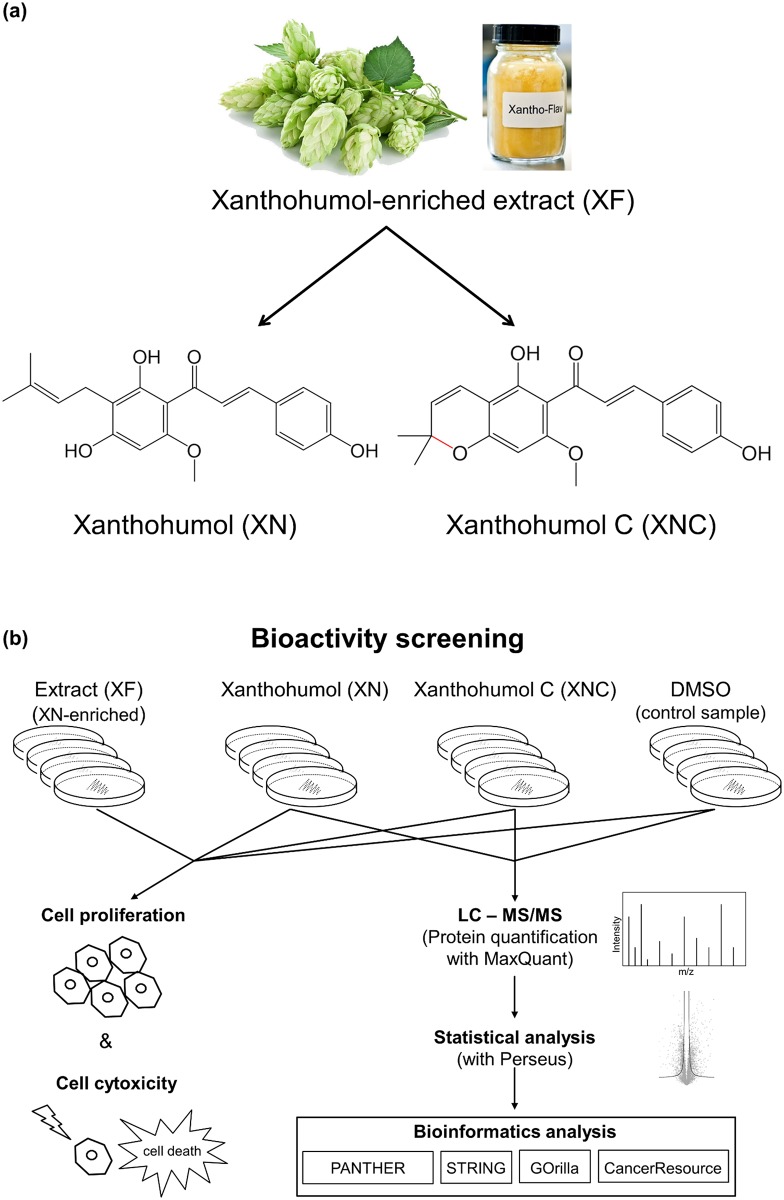
(a) Molecular structures of the hop compounds xanthohumol (XN) and xanthohumol C (XNC) obtained from the xanthohumol-enriched hop extract Xantho-Flav (XF). (b) Overview of the total workflow of the study.

Hop (*Humulus lupulus L*.) is well known as a medical plant with many bioactive effects. Due to the trend of evaluating natural compounds for its diverse bioactive effects, hop has been attracting increasing interest as a natural resource for promising biologically active phenolic compounds. In this context, natural compounds have shown promise due to their antimicrobial, antiviral and also anticarcinogenic or (chemo)preventive effects [1, 2]. Hop contains a huge variety of prenylated phenolic compounds [3–6]. The most abundant prenylated chalcone in hops is xanthohumol (XN) [7]. Great importance has been placed on this compound due to its multiple biological activities, including anti-inflammatory [8], neuro-protective [9, 10], anti-microbial [11, 12], anticarcinogenic [13–15] and even radio-sensitizing [16] effects. Furthermore, promising results from in vivo and in vitro studies with several cancer cell lines, including MCF-7 breast cancer cells, have shown its antiproliferative activity [13–15, 17]. Xanthohumol shows co-action with several antibiotics [11, 12], but is less active in combination with other hop compounds [12] against gram-positive bacteria. Moreover, XN showed anti-obesity effects by inhibiting the differentiation of preadipocytes and inducing apoptosis in mature adipocytes by using the mouse cell line 3T3-L1 [18, 19]. All these explorations strongly suggest XN and its analogues as potential compounds for the prevention and treatment of many diseases [2, 3, 6, 7, 14, 16, 20–23]. Especially naturally occurring minor chalcones from hops gained interest due to their different investigated properties. Dietz et al. showed an efficacy improvement of bioactive hop compounds linked to the concentration of minor compounds in the natural extract [5]. Interestingly, many of these seem to be even more bioactive in comparison to XN [4, 7, 24, 25]. 8-prenylnaringenin is known as one of the most potent phytoestrogen [6], desmethylxanthohumol shows e.g. anti-oxidant activity [26], isoxanthohumol inhibits angiogenesis [27], and 6-prenylnaringenin has anti-fungal activities against Trichophyton spp. [28]. Another recently upcoming minor chalcone is xanthohumol C (XNC), which has lately received much attention due to its antiproliferative, cytotoxic [15, 17], neuro-protective [9], and anti-oxidative activities [24, 29, 30]. Xanthohumol C, also called dehydrocycloxanthohumol, was first identified by Stevens et al. [31] and its molecular structure differs to xanthohumol due to a ring closure of the prenyl-side chain with the hydroxyl group at position 4’ (see Fig 1A). Miranda et al. [15] already showed a significant antiproliferative and cytotoxic effect of various prenylated flavonoids and assumed an inhibition capacity only of XNC at lower concentrations compared to XN and iso-xanthohumol for the growth of MCF-7 breast cancer cells. In contrast, Popłoński et al. [17] recently showed a lower in vitro antiproliferative activity of XNC compared to XN in prostate, colon, and breast cancer cell lines. In another study, XNC was identified as the most active compound within a group of hop-derived prenylflavonoids for the differentiation of neuronal precursor cells [9]. While XN and other major phenolic hop compounds are already well characterized in literature, pharmacological data concerning minor compounds such as XNC are scarce due to its limited availability via isolation from natural sources and therefore is still in the process of investigation [32]. In addition, in natural products studies it is often difficult to link a certain bioactivity to a single compound due to the presence of many minor impurities and their unknown influence on the analyzed target compound.

The potential of liquid-liquid chromatography, i.e. countercurrent chromatography (CCC) and centrifugal partition chromatography (CPC), has recently been shown to provide even minor hop compounds in sufficient amount and high purity for antimicrobial or pharmacological testing [33]. In the current study, a XN-enriched hop extract (Xantho-Flav^™^, XF) was used as a natural resource containing 65–85% XN and a huge variety of other different minor compounds such as XNC (0.24–2.2%). Subsequently, the bioactive effects of purified XNC (>99.8%) were evaluated and compared to pure XN (>98.6%). Both compounds were previously gained from XF with liquid-liquid chromatography [33].

The objective of this study was to compare the effects of single compounds to the natural crude resource, the XN-enriched hop extract XF, as a complex multicomponent mixture. XF contains a huge variety of minor compounds that theoretically could interact with each other and hence enhance or reduce the bioactive effect, or evoke the biological effect only in combination with each other.

Mass spectrometry based proteomics has been exploited as a powerful technology for capturing complex information about proteome structure and function. This can be used to gain an understanding of the biological processes ongoing in cellular samples of interest [34–39]. In the context of compound-protein interactions quantitative proteomics can provide molecular insights into biological pathways and processes influenced by a given compound [40, 41].

In this study, we established an experimental approach for the bioactivity analysis of promising minor hop compounds (see flow chart in Fig 1B). First, the dose dependent antiproliferative and cytotoxic effect of XN and XNC were evaluated in MCF-7 cell culture experiments and compared to a complex crude hop extract, the commercially available XN-enriched hop extract (XF). Subsequently, protein expression in the MCF-7 cell line after XN and XNC treatment was analyzed by label-free data-dependent acquisition proteomics and the results evaluated by various bioinformatics tools, such as Perseus, PANTHER, STRING, and GOrilla. Such analysis could give a fast overview of possible functions that can guide for further specific analysis. This enables a targeted approach and consequently also reduces experimental time as well as sample amounts of such rare highly purified minor natural products.

## Materials and methods

### Chemicals

The pre-concentrated commercial xanthohumol (65–85%, HPLC) crude extract, Xantho-Flav (XF), was supplied by the company Hopsteiner (Germany). Xanthohumol (>98.6%, HPLC) and xanthohumol C (>99.8%, HPLC) were purified by liquid-liquid chromatography (CCC/CPC) as described in a previous work [33]. Chemical structures were drawn with Marvin 17.2.27.0, 2017, ChemAxon http://www.chemaxon.com).

### Cell culture

The MCF-7 breast cancer cell line was kindly provided by the Chair of Proteomics and Bioanalytics (Technical University of Munich, Germany), originally purchased from the National Cancer Institute (NCI, USA). The complete growth media of MCF-7 cells consisted of Gibco (Dulbecco's Modified Eagle Medium, DMEM, high glucose) from Thermo Fisher Scientific Inc. (USA) and 10% fetal bovine serum (FBS) from Sigma-Aldrich (USA). The media for bioactivity assays composed of DMEM, high glucose, HEPES, no phenol red from gibco (Thermo Fisher Scientific Inc., USA) and 5% FBS. For sub-culturing cells phosphate-buffered saline (PBS) from Merck KGaA (Germany) and a trypsin-EDTA solution from Sigma-Aldrich (USA) were used.

Proliferation was determined by the CellTiter 96 Aqueous Non-Radioactive Cell Proliferation Assay from Promega (USA), which contains 3-(4,5-dimethylthiazol-2-yl)-5-(3-carboxymethoxyphenyl)-2-(4-sulfophenyl)-2H-tetrazolium, inner salt (MTS) and the electron coupling reagent phenazine methosulfate (PMS).

### Equipment and implementation of cell culture bioactivity tests

The cells were cultivated in T-75 (75 cm^2^) or T-25 (25 cm^2^) flasks (Falcon Tissue Culture Flasks, Corning Inc., USA) at 37°C in a humidified atmosphere of 95% air / 5% CO_2_, until 80% confluency. In each new T-75 flask between 8.5 ∙ 10^5^ and 1.1 ∙ 10^6^ MCF-7 cells were plated. For bioactivity screenings, cells were centrifuged (120 rcf, 20°C, 10 min) and the complete growth medium was exchanged with media for bioactivity assays. Per well 5 000 MCF-7 cells were plated into a 96-well microtiter plate (Corning Inc., USA). After 1 day of incubation, the cells were treated with the test substances XN, XNC and XF in varying concentrations (100, 75, 50, 25, 10, 7.5, 5, 2.5, 1, 0.1, 0.01 μM) dissolved in DMSO (maximal 0.2% v/v). As control DMSO without a component was used (0.2% v/v).

At the day of treatment (day 0), a proliferation assay was conducted on cells without stimulation (without bioactive compounds) for calculating cytotoxicity. After incubation for 2, 4 and 6 days, the proliferation assay was performed on all treated cells and the control samples. Therefore, old medium was removed, cells were washed with 150 *μ*L PBS, and 100 *μ*L of fresh medium was added for the bioactivity tests. The MTS/PMS-solution (ratio 20 to 1) was added (16 *μ*L) and incubated for 90 minutes at 37°C, 5% CO_2_. As described in the technical bulletin (Promega Corp., USA), MTS is converted to an aqueous, soluble formazan by dehydrogenase enzymes found in metabolically active cells [42]. The absorbance of the formazan was recorded at 490 nm by the “infinite M200 multimode microplate reader” (Tecan Group Ltd., Switzerland). The experiments were performed in triplicate.

Antiproliferative effects were calculated as a ratio of OD_490nm_ in XN, XNC, and XF treated wells and the wells treated with DMSO. Cytotoxicity was determined as the ratio between OD_490nm_ in a treated well and OD_490nm_ at day 0 (day of treatment) (technical bulletin, Promega Corp., USA). Statistical analyses were determined by Student's *t*-test. In all cases, *P* values <0.05 were considered statistically significant.

### Cell culture preparation for proteomics analysis

For the proteomic experiments, MCF-7 cells were cultured as described in the cell culture section before and transferred to T-25 cm^2^ flasks. After one day of incubation, cells were treated with XN, XNC, and DMSO as control. For the treatment, the IC_50_ concentration determined by antiproliferative assays was used, respectively (XN: 12.25 *μ*M, XNC: 4.18 *μ*M). After two more days of incubation, medium was removed completely and cells were washed two times with PBS. Finally, 1 mL PBS was added, cells were scraped from the surface of the flask and transferred to an Eppendorf Protein LoBind Tube (Eppendorf, Germany). Cells were centrifuged at 16000 rcf, for 10 min, and 4°C. Supernatant was completely discarded and the pellet was shock frozen in dry ice, containing 80% EtOH and stored at -80°C until protein extraction was performed. The pelletized MCF-7 cells were sampled in quadruplicates.

### Sample preparation for proteomics analysis

The stored cell pellets were thawed slowly on ice and resuspended in lysis buffer (8 M urea, 5 mM EDTA di-sodium salt, 100 mM (NH_4_)HCO_3_, 1 mM Dithiothreitol (DDT), 10% (v/v) protease inhibitor cocktail (SigmaFast 10x stock), pH 8.0) by thoroughly up- and down-pipetting and subsequent ultrasonication for 40 seconds. Total protein concentration of the lysate was determined using the BCA method (Pierce BCA protein assay kit, Thermofisher, Germany). 100 *μ*g protein extract was used per sample for in-solution digestion. Proteins were reduced with 10 mM DTT (at 30°C for 30 min), and subsequently carbamidomethylated with 55 mM chloroacetamide in the dark at room temperature for 60 min. Proteins were digested with 1 *μ*g trypsin (1:100 trypsin:protein) for 3 hours at 37°C and another 1 *μ*g of trypsin overnight at 37°C. Digested peptide samples were desalted according to the manufacturer’s instructions by C18 solid phase extraction using Sep-Pak columns (Waters, WAT054960) [43]. Purified peptide samples were dried in a SpeedVac and resuspended in 2% acetonitrile, 98% H_2_O, 0.1% formic acid to a final concentration of 0.25 *μ*g *μ*L^-1^ as determined by Nanodrop measurement.

### Liquid chromatography and tandem mass spectrometry

Generated peptides were analyzed on a Dionex Ultimate 3000 nano LC system coupled to a Q-Exactive HF mass spectrometer (Thermo Scientific, Germany). Peptides were delivered to a trap column (75 *μ*m × 2 cm, self-packed with Reprosil-Pur C18 ODS-3 5 *μ*m resin, Dr. Maisch, Ammerbuch) at a flow rate of 5 *μ*L min^-1^ in solvent A0 (0.1% formic acid in water). Peptides were separated on an analytical column (75 *μ*m × 40 cm, self-packed with Reprosil-Gold C18, 3 *μ*m resin, Dr. Maisch, Ammerbuch) using a 120 min linear gradient from 4–32% solvent B (0.1% formic acid, 5% DMSO in acetonitrile) and solvent A1 (0.1% formic acid, 5% DMSO in water) at a flow rate of 300 nL min^-1^. The mass spectrometer was operated in data-dependent acquisition (DDA) mode, automatically switching between MS and twenty MS2 spectra.

MS1 spectra were acquired over a mass-to-charge (*m/z*) range of *m/z* 360–1 300 at a resolution of 60 000 (at *m/z* 200) using a maximum injection time of 10 ms and an AGC target value of 3e6. Up to 20 peptide precursors were isolated (isolation window *m/z* 1.7, maximum injection time 50 ms, AGC value 2e5), fragmented by HCD using 25% NCE and analyzed at a resolution of 30 000 with a scan range from *m/z* 200 to 2 000. Precursor ions that were singly-charged, unassigned or with charge states >6+ were excluded. The dynamic exclusion duration of fragmented precursor ions was 35 s.

### Peptide and protein identification, intensity-based absolute quantification (iBAQ) and label-free quantification (LFQ) of proteins

The proteomic experiments were performed with MCF-7 cells treated with XN or XNC as well as with a control sample containing only DMSO as a solvent. Peptide and protein identification and label free quantification were performed with MaxQuant (version 1.5.8.3, http://www.coxdocs.org/doku.php?id=:maxquant:start) [44] (see MaxQuant configuration in S3 File) by searching the MS2 data against all protein sequences obtained from UniProt—Reference proteome Homo sapiens (UP000005640, canonical, 20 236 reviewed entries, last modified on July 18, 2017) using the embedded search engine Andromeda [45]. Carbamidomethylated cysteine was a fixed modification; oxidation of methionine, and N-terminal protein acetylation were variable modifications. Trypsin/P was specified as the proteolytic enzyme and up to two missed cleavage sites were allowed. Precursor and fragment ion tolerances were 10 ppm and 20 ppm, respectively. Label-free quantification (LFQ) [46] and data matching between consecutive analyses were enabled within MaxQuant. Search results were filtered for a minimum peptide length of seven amino acids, 1% peptide and 1% protein FDR plus common contaminants and reverse identifications. Each condition was monitored in four biological replicates (see all identified peptides in S1 File).

Intensity-based absolute quantification (iBAQ) values, which are proportional to the molar protein quantities in the samples, were used to relatively compare the concentration of different proteins within one sample. iBAQ protein intensity values were retrieved from the MaxQuant software as the sum of all peptide intensities per proteins divided by the number of theoretical peptides per protein. For protein quantification between samples, label free quantification (LFQ) [46] intensities were obtained (see all identified protein groups in S2 File).

After raw data processing with MaxQuant, quality control (QC) was performed by using the R-based QC pipeline *Proteomics Quality Control* (*PTXQC* package). Measured bias, consistency, and errors were analyzed by creating a QC report (see S4 File) containing a set of QC metrics [47].

### Statistical analysis with Perseus

MaxQuant results were further statistically analyzed using the MaxQuant associated software suite Perseus (version 1.6.0.7, http://www.coxdocs.org/doku.php?id=perseus:start). First, raw data from XN and XNC treated replicates were filtered by excluding potential contaminants, reverse and only identified by site proteins leading to a table of all identified proteins (S2 File). This data set was further filtered based on the criterion that at least 3 non-zero values must be present in at least one treatment or control group. Missing values were imputed on the basis of a Gaussian distribution using the Perseus algorithm to allow for statistical analysis, resulting in all quantified proteins (S5 File). Statistical significance was assessed by the Student’s t-test between control and treated groups. Quantitative differences between the analyzed groups were considered to be statistically significant for adjusted p < 0.05. The Log2 (Fold Ratios) of the proteins were calculated in all condition comparisons and means and as well as standard deviations (SD) of the differences were calculated. Proteins displaying large magnitude changes within a defined cutoff (1 SD) were classified as differentially expressed (DE) proteins and visualized in volcano plots. The data were expressed as the mean (± 1 SD) and further categorized as: upregulated or downregulated proteins (S6–S9 Files). The significance of the differences was determined by the Student’s t-test.

### Bioinformatics analysis

The identified proteins were classified and compared by different bioinformatics tools, such as PANTHER, STRING, GOrilla, to obtain further information about protein annotation, molecular function, biological process, subcellular localization, protein interactions and their potential pathways.

The PANTHER classification system (version 13.0, http://www.pantherdb.org) was used for protein identification and classification in terms of molecular function, biological process and cellular component [48]. For that, protein IDs (UniProtKB) of identified, quantified, and DE proteins including up- and downregulated DE proteins after XN and XNC treatment were submitted to PANTHER and *Homo sapiens* was selected as the organism. In addition to the obtained protein amounts, the mapped protein IDs were subsequently statistically analyzed with the chi-squared test (X^2^) for comparison between the samples. Again, p<0.005 indicates significance and p<0.001 was selected for indicating a highly significant difference.

The signaling pathways and protein interactions of proteins with changed expression levels were identified with the help of the STRING-tool (Version 10.5, https://string-db.org). In analogy to the PANTHER analysis, protein IDs were submitted and active interaction sources were selected: experiments, co-expression, neighborhood, gene fusion, and co-occurrence. For the protein network visualization, only the main interactions were considered. A default medium confidence score (>0.4) was used for XN. In terms of XNC, a high confidence score (>0.7) was used in order to reduce the high number of proteins. However, no protein interactions at high confidence score were found for XN. In addition, disconnected nodes in the network were hidden [49, 50].

Further, visualization of shared DE proteins between XN and XNC treated cells was achieved with the online plotting tool Venny 2.1.0 (Version 2.1.0, http://bioinfogp.cnb.csic.es/tools/venny). The intersections and complements are plotted based on protein IDs.

A functional enrichment analysis was performed for the identification of significantly overrepresented or underrepresented proteins in molecular function, biological process and cellular component. For that, the protein IDs were submitted for the organism *Homo sapiens* to the tool GOrilla (http://cbl-gorilla.cs.technion.ac.il) [51]. For each of the different Gene Ontology (GO) categories, an enrichment factor, FDR p-values and corresponding p-values were calculated (details see S1 and S2 Tables). Accessions with p-values of more than 10^−3^ and FDR adjusted p-values of more than 10^−2^ were automatically removed from the corresponding enrichment table.

Single proteins were also analyzed by open access database CancerResource (http://data-analysis.charite.de/care/). Drug targets were searched by gene identifiers, and possible cancer relevant pathways and mutation profiles were examined in more detail.

## Results and discussion

### Evaluation of antiproliferative effects of xanthohumol (XN), xanthohumol C (XNC), and a XN-enriched extract (XF) on MCF–7 breast cancer cell line

In previous studies, XN and several analogues demonstrated antiproliferative and cytotoxic effects in human cancer cell lines, such as human breast cancer cells (MCF-7), colon cancer cells (HT-29) or ovarian cancer cells (A-2780).[15] The activities of xanthohumol derivatives are highlighted as promising new bioactive compounds.[25, 52] In addition to their various bioactive effects, natural products usually show a low toxicity and can therefore be used as favorable preventive substances or as additives that enhance the effect of e.g. cytostatic drugs against breast cancer. In this study, XNC was evaluated for its antiproliferative and cytotoxic effects on breast cancer cells (MCF-7) and its activity is compared to the reference XN and the XN-enriched hop extract Xantho-Flav (XF) containing a high amount of XN (65–85%) as well as a huge variety of different minor hop compounds in very low concentrations (including 0.24–2.2% of XNC).

**Fig 2:**
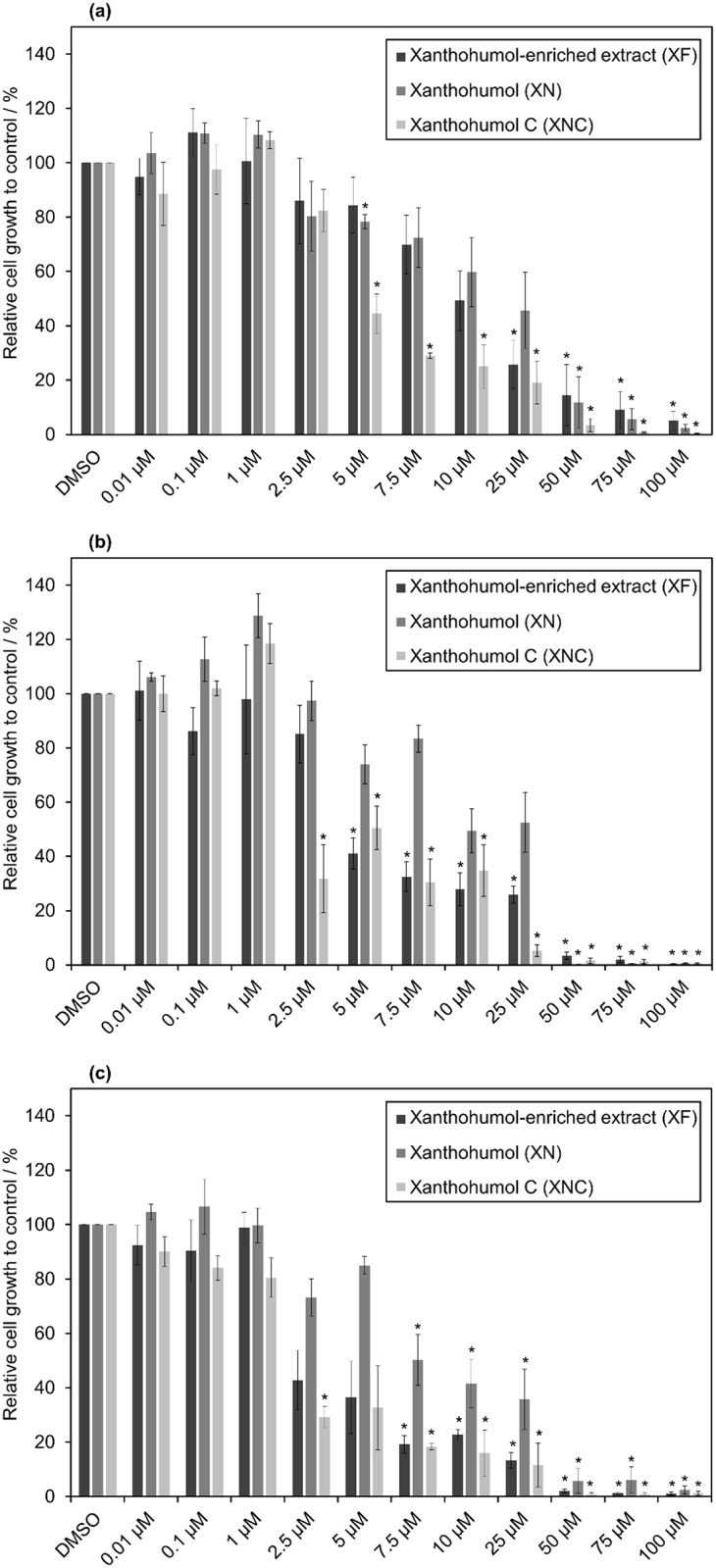
Relative cell growth of MCF-7 cells determined by MTS-assay after 2 (a), 4 (b) and 6 (c) days of incubation at different concentrations of XF, XN and XNC (*statistical significance to control, p-value<0.05).

The antiproliferative effects of XN, XNC and XF on MCF-7 cell line were determined with the MTS-assay (for details see Materials and methods section) [42]. In Fig 2 the relative growth-inhibitory activity of the three compounds in MCF-7 cells after a treatment of 2 (a), 4(b) and 6(c) days is shown.

Cells incubated with XN always grew best. Interestingly, the raw extract XF, which consists of about 80% XN, inhibited cell growth more than pure XN. This seems to indicate strong effects of minor components included in XF. One of these minor compounds is the minor hop chalcone XNC. Cells incubated only with XNC, showed the strongest growth inhibition.

In addition, the inhibition concentration of the compounds at 50% cell growth inhibition (IC_50_) was determined for all three compounds. In all cases, the IC_50_ was lowest after 6 days of incubation. There was no decreased sensitivity of the cancer cells after 6 days of exposure, whereby the generation of resistances can be excluded for this period of time. After all three periods of treatment, the IC_50_ of XNC was lowest and the one of XN the highest. After two days of incubation, the IC_50_ of XNC (4.18 *μ*M) was by a factor of 2 lower compared to XF (8.84 *μ*M), and only a third compared to XN (12.25 *μ*M). After four days (XNC: 1.9 *μ*M, XF: 4.3 *μ*M, XN: 8.8 *μ*M) and six days (XNC: 1.70 *μ*M, XF: 2.2 *μ*M, XN: 7.1 *μ*M), the difference between XNC and XN was even more distinct. Interestingly, XN and XF show similar growth after 2 days of incubation (Fig 2A), but clearly differ after 4 (Fig 2B) and especially 6 days (Fig 2C). This indicates some kind of a delayed effect of XF, resulting in a stronger effect of XF compared to the pure compound XN. Statistical significance was determined by Student’s *t*-test (p-value < 0.05). Interestingly, only XNC showed a significant relative cell growth inhibition at 2.5 *μ*M after 4 and 6 days of incubation. In accordance with literature [15, 24, 53], XNC shows significant antiproliferative effects compared to XN. It is noteworthy that a higher antiproliferative activity of XN and in particular XNC after two days of incubation was observed compared to the data of Popłoński et al. [17], who showed a higher IC_50_ concentration for both compounds (XN: 8.1±0.8 μM, XNC: 15.0±1.8 μM) as well as a different tendency with XN as the more active compound. A possible reason for the different tendency might be the use of different purification methods. An isolation of XN and XNC based on flash chromatography or liquid-liquid chromatography could result in different purities and hence a different interaction of the target compound with minor impurities that could potentially influence activity. Another reason could be a difference in the cell line itself due to the unstable genome of cancer cells that can result in different gene expression and properties between different labs. Other effects like isomerization of XN and XNC induced by thermal treatment [6] or alkaline conditions [54] are rather unlikely due to the selected experimental conditions and the relatively short experimental time of few days. Nevertheless, all results show an in vitro antiproliferative activity in a similar concentration range and emphasize the anticarcinogenic potential of the investigated minor hop compounds. In comparison to other promising natural phenolic agents, such as resveratrol (IC_50_: 100μM) or curcumin (IC_50_: 35μM), XNC shows a much higher antiproliferative effect against MCF-7 cells after two days [55].

### Cytotoxic effects of XN, XNC and XF on MCF-7 cells

**Fig 3:**
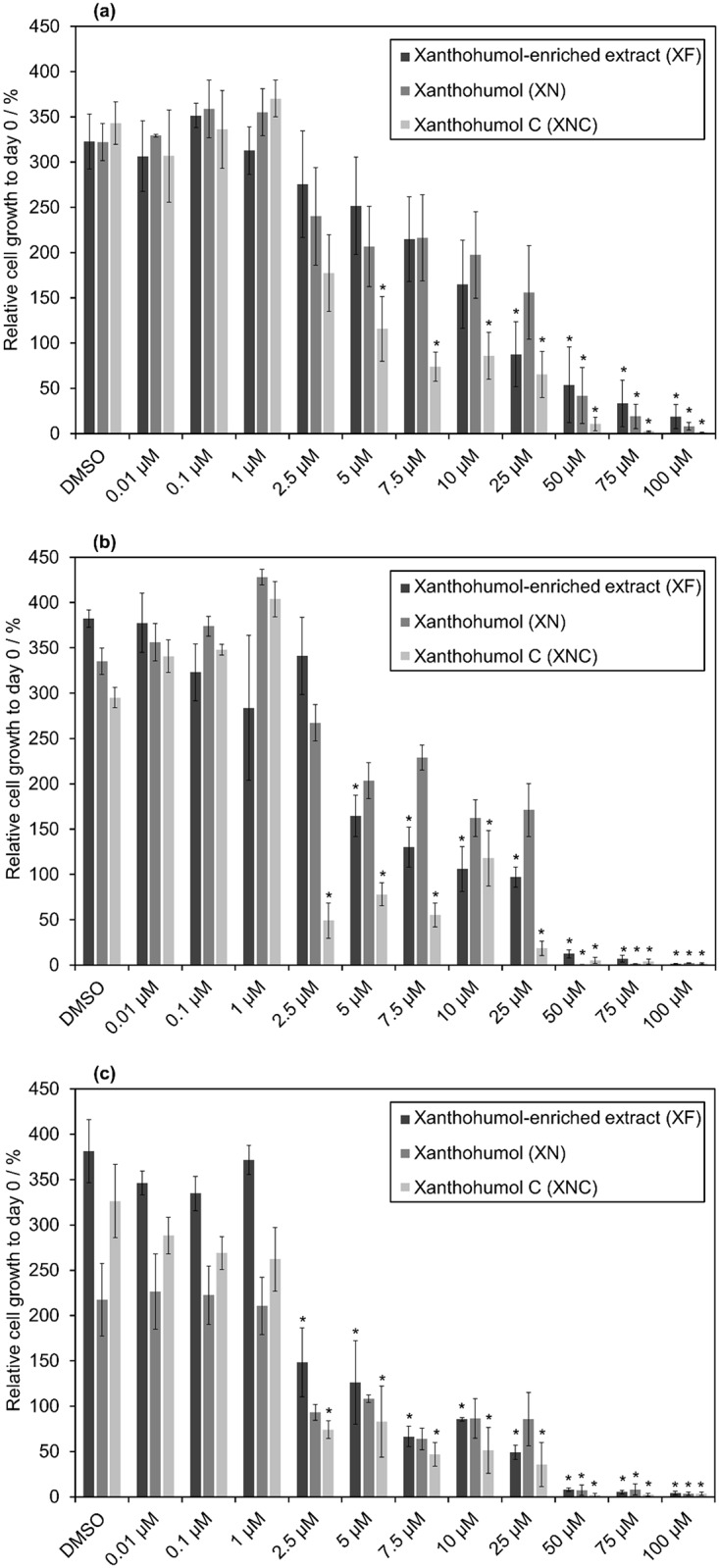
Cytotoxic effect on MCF-7 cells after 2 (a), 4 (b) and 6 (c) days of incubation with different concentrations of XF, XN and XNC (* statistical significance to control, p-value<0.05). Note: Experiments with XN treatment in Fig 3B were only performed in duplicate.

Cytotoxicity was calculated in analogy to Miranda et al.[15] by comparing OD units of treated cells with OD units of untreated cells at day 0, which is the start of the compound incubation. Cell death was indicated by lower cell viabilities after treating the cells compared to cell viability before treatment. The cytotoxic effect on the MCF-7 cell line is shown in Fig 3 after 2 (a), 4 (b) and 6 (c) days of incubation with different concentrations of XN, XNC, and XF. After two days of incubation, XNC was the most cytotoxic compound and showed cell death even at a concentration of 5 μM.

After 4 and 6 days of incubation this effect was already observed at a concentration of 2.5 *μ*M (XNC). Above 50 *μ*M all of the substances were cytotoxic to MCF-7 cells. At a concentration below 1 *μ*M the cell death was not affected by the components. Statistical significance was determined by Student’s *t*-test (p-value <0.05) indicating cell death induced by the three compounds. Interestingly, after 2 days, only XNC showed cytotoxic effects at low concentrations, while after 4 and 6 days the difference in cytotoxicity between the compounds is decreasing.

From the cell culture studies, i.e. antiproliferative and cytotoxic analysis, it can be assumed that XF as a complex mixture might affect various cell processes just like the single compounds. For a deeper understanding of the effects on molecular level, pure compounds are necessary in order to avoid misinterpretation of the experimental results and to find the effects of the actual single target compounds.

### Quantitative proteomics analysis of MCF-7 cells

In order to get insights into pharmacological influence or anticarcinogenic behavior of the hop compounds xanthohumol C (XNC) and xanthohumol (XN), a quantitative proteomics approach was used to analyze the global proteome of MCF-7 breast cancer cells in order to find possible molecular mechanisms of XN and XNC treatment [56, 57]. Although proteomics studies have been applied to hop and its bioactivity before [58–60], data about how minor hop compounds, such as XN and XNC, affect the proteome of cancer cells are still lacking. In this study, the global proteome of MCF-7 human breast cancer cells was quantified after treatment with XN and XNC. Further, the involved biological processes are characterized using bioinformatics analysis tools. This global proteome characterization enabled an evaluation of possible effects of XN and XNC in order to generate new hypotheses about how these compounds might influence the function of the cells on a molecular level. This strategy can easily be extended and applied to screenings of a wider range of other bioactive minor hop compounds as well as to other cancer cell lines.

**Fig 4:**
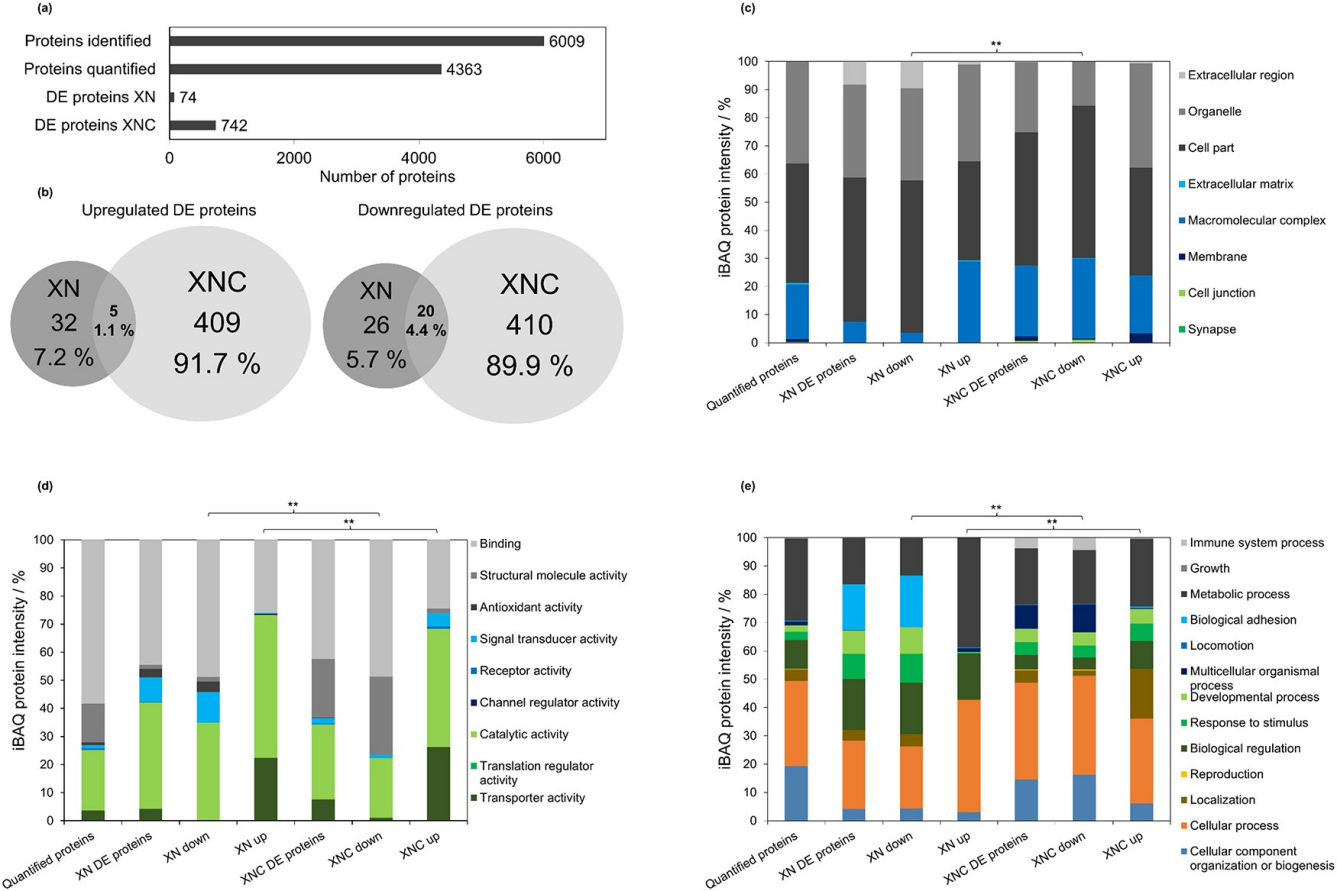
Global proteome characterization from MCF-7 cells after treatment with XN and XNC at IC_50_ concentration. (a) Comparison of the number of identified, quantified, and differentially expressed (DE) proteins. (b) Comparison of shared up- and downregulated DE proteins (c) Predicted cellular localization of quantified and DE proteins in percentage based on iBAQ intensities (d) Predicted molecular function of quantified and DE proteins in percentage based on iBAQ intensities (e) Predicted biological process of quantified and DE proteins in percentage based on iBAQ intensities. (*significant difference, p < 0.005; **highly significant difference, p < 0.001).

The cells were treated with the IC_50_ concentration (XN: 12.25 *μ*M, XNC: 4.18 *μ*M) for two days in quadruplicate and measured by label-free data-dependent acquisition proteomics. Additionally, DMSO was used as a control condition. As shown in Fig 4A, in total 6 009 proteins could be identified using the software MaxQuant, which covers about 30% of the 20 236 entries from UniProt database reference proteome. From these, 4 363 proteins were quantified with good reproducibility (at least three valid measurements out of four replicate measurements), of which 74 were differentially expressed (DE) (41 down- and 33 upregulated) in case of XN (S1 Fig including molecular functions) and 742 (366 down- and 376 upregulated) in case of XNC (S2 Fig including molecular functions). The number of DE proteins seems to be concomitant to the higher activity and growth inhibition of XNC in the cell culture experiments. A higher effect of XNC could cause stronger defense mechanisms of the MCF-7 cell, which consequently results in more DE proteins. The comparison between the XN and XNC regulated proteome shows not only a large difference in the total number of DE proteins but also in the low number of shared DE proteins. An overlap of only 5 upregulated (1.1%) and 20 downregulated (4.4%) proteins as shown in Fig 4B was found. In addition, the shared proteins were spread over different possible pathways and functions, so that no specific common function could be found. This indicates different target structures and modes of action between XN and XNC.

Next, a protein classification was performed by applying a gene ontology (GO) analysis with the PANTHER classification system on various functional and cellular levels. Quantified and DE proteins were analyzed in terms of cellular localization, molecular function and biological processes (Fig 4C–4E). For all three categories, a difference (p-value< 0.001) between DE proteins after treatment with XN and XNC was determined. In addition, DE proteins were further analyzed by differentiating DE proteins in up- and downregulated proteins after treatment of XN or XNC and statistical significance was calculated by the chi-squared test. For XN and XNC comparisons, significance was conducted between up- and downregulated proteins respectively. A complete coverage of all subcellular compartments was observed, revealing a successful sample preparation including harvest, cell disruption, and digestion.

Applying the analysis of cellular localization (Fig 4C), it was possible to associate DE proteins with mainly organelle, cell part, macromolecular complex, membrane and extracellular regions. The predicted cellular localization after XN and XNC treatment clearly differs in downregulated proteins. In terms of XN treated MCF-7 cells a higher percentage of DE proteins from extracellular region were downregulated, while for XNC a higher amount of proteins from macromolecular complexes was downregulated. In upregulated proteins, XNC and XN mainly differ in the higher amount of upregulated membrane proteins for XNC.

Concerning molecular function (Fig 4D), mostly proteins assigned to binding and catalytic activity could be found. Both downregulated and upregulated proteins are different between XN and XNC. Noteworthy is that the signal transducer activity is downregulated in XN compared to an upregulation in XNC. In addition, the structural molecule activity is more severely downregulated with XNC.

In allocating the DE proteins to certain biological processes (Fig 4E), downregulated proteins differentiate in biological adhesion in XN, and immune system processes and multicellular organismal processes in XNC. In terms of upregulated proteins, a higher amount of proteins involved in localization, developmental processes and response to stimulus after treatment with XNC was observed.

In general, a multifactorial response from treatment with hop compound XN and XNC becomes apparent from this analysis. Due to many different influenced proteins and functions, the biological activity of XN and XNC cannot be assigned to one specific cluster of function or target structure.

**Fig 5:**
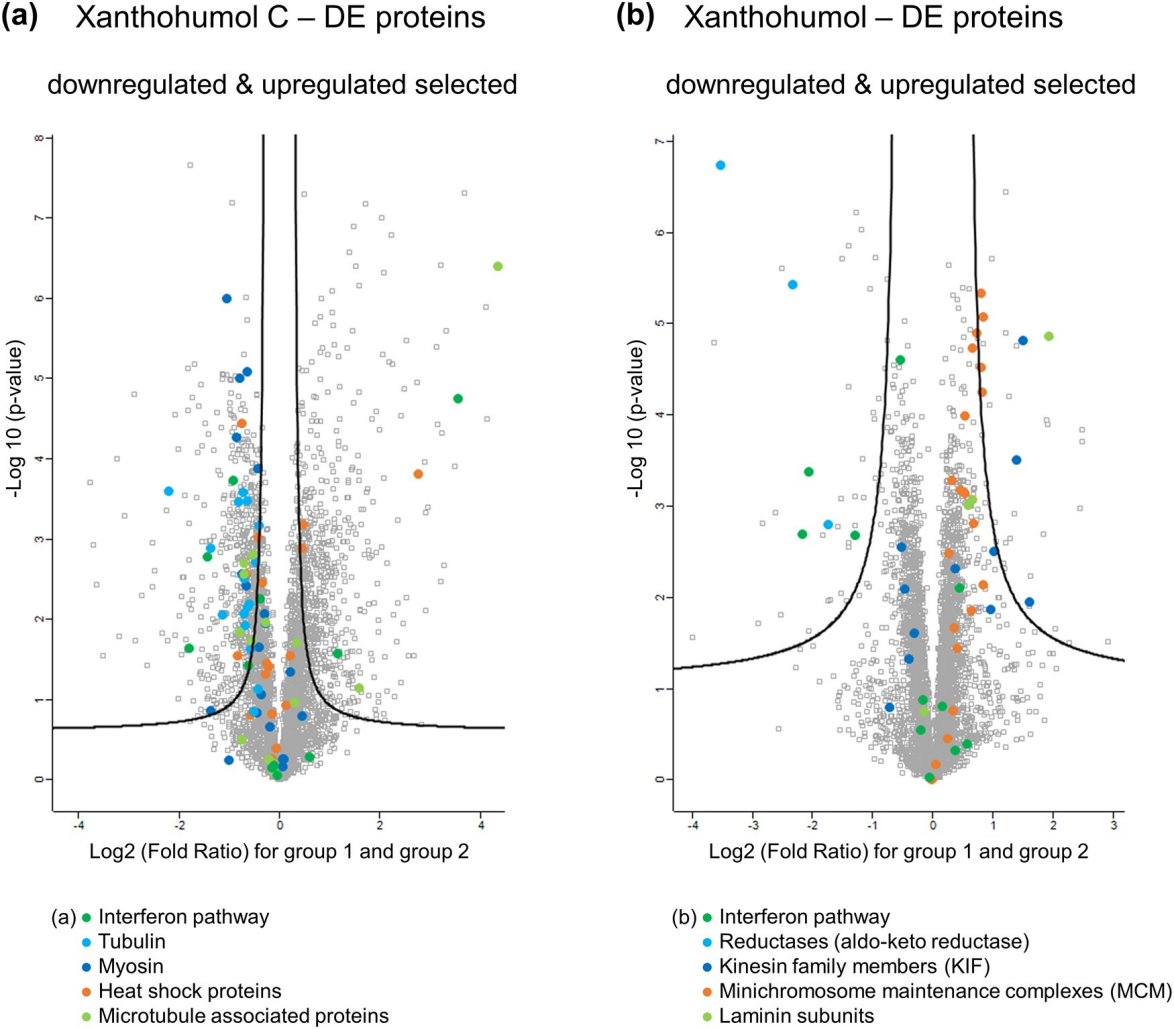
Volcano plots represent significant differences between MXF-7 treated cells (group 1) and DMSO control cells (group 2) for XNC (a) and XN (b). Significant up- and downregulated proteins that could be assigned to one function or family are marked. The protein names are listed in supporting information S6–S9 Files.

Next we specifically investigated statistically significant differences between XN or XNC treated MCF-7 cells and the DMSO control cells as shown in the volcano plot analysis in Fig 5. In MCF-7 cells after treatment with XN, reductases (aldo-keto reductase) and proteins involved in the interferon pathway were significantly downregulated. In contrast, upregulated proteins (kinesin family members, KIF, and minichromosome maintenance complexes, MCM) were allocated to the laminin pathway. Proteins from the laminin pathway also contribute to cell growth and cell adhesion and are therefore involved in catalytic activity, binding and receptor activity. This is also in accordance to functional network analysis (see S3 and S4 Figs), where a strong interaction of these upregulated proteins could be observed. Besides kinesin family members (KIF) and minichromosome maintenance complexes (MCM), also aurora kinases (AURK) were present in the network. These proteins are implicated in mitotic spindle elongation, cell cycle, and deoxyribonucleic acid (DNA) replication. This is expected, since these proteins (i.e. kinesin family members) were shown to be overexpressed associated with breast cancer cell growth [61]. Based on western blot analysis, Shimo et al. [61] suggested this protein family as a promising molecular target for anticancer treatment. A downregulation could possibly suppress the growth of such breast cancer cells. In addition, MCM proteins were described as promising markers for targeting cancer cells, since they are involved in DNA replication and therefore mostly upregulated in cancer cells [62, 63]. Aurora kinases B and C are also upregulated in correspondence with cancer cell growth and implicated to several biological cell processes. Consequently, the described upregulation mainly confirms the cell stress response after treatment with XN.

On the side of downregulation after treatment with XN, especially proteins involved in type I interferon signaling pathway were affected, including SQSTM1, ISG15, OAS1, OAS3, IRF9, STAT1, PARP9, and DTX3L. In general, interferon is involved in the JAK/STAT (Janus kinase/ signal transducer and activator of transcription) signaling pathway and hence induces cell apoptosis (see also cancer data base: http://data-analysis.charite.de/care/). Musgrove et al. described an in vitro downregulation of the interferon pathway in tamoxifen resistant breast cancer cells [64]. Furthermore, they concluded from the downregulation of the interferon pathway among others, a reason for therapeutic resistances against tamoxifen and other antiestrogens. Consequently, a downregulation of proteins involved in the type I interferon signaling pathway might be an adjustment of MCF-7 cells to treatment with XN. To sum up the xanthohumol analysis, a general stress response of MCF-7 cells was found after treatment.

Compared to treatment with XN, a much higher number of proteins were differentially expressed after treatment with XNC. As mentioned before, this could be associated with the higher activity of XNC. Mostly proteins part of tubulin and myosin were downregulated (see functional network in S4(b) Fig), whose molecular function is involved in binding and catalytic activity. In contrast, so called heat shock proteins (HSP) signaling cell stress and influencing structural molecule activity were upregulated (see HSPA5, HSPA13, HSP90B1, HSP40 in functional network, S4(a) Fig). An enrichment analysis of the different functional categories of all upregulated proteins leads to an overrepresentation of microtubule-associated protein 1 light chain 3 alpha and beta, and beta 2 (MAP1LC3A / MAP1LC3B / MAP1LC3B2), which are involved in the cellular response to nitrogen levels, autophagy of mitochondrion, and mitochondrion disassembly. However, these enriched upregulated DE proteins occurred as single nodes only and hence, could not be assigned to a certain network within the functional network analysis. Nevertheless, the upregulation of these proteins might explain the cytotoxic effect of XNC in MCF-7 cells. A list of all enriched molecular functions is provided in supplementary S1 and S2 Tables including GO terms and description. The upregulated heat shock proteins are generally expressed as a reaction to endoplasmic reticulum (ER) stress and are involved in protein folding. In addition, they protect proteins’ secondary structure under extreme conditions and are involved in mitogen-activated protein kinases (MAPK) signal pathways. This might be a reason for cell apoptosis as a consequence after cell treatment with XNC (see also cancer data base: http://data-analysis.charite.de/care/). As reported by Nollen et al. [65], both upregulation and downregulation of these proteins, i.e. HSP70 or HSP90, can result in aberrant growth control, developmental malformations and cell death. This might depend on the relative amount of these proteins. In addition, Chen et al.[66] described a downregulation of a different HSP (i.e. HSP27) after MCF-7 treatment with the antitumor drug doxorubicin and showed a target related action on the HSP27 expression. It is assumed that the influence on the HSP level might be useful for the control of breast tumor growth [67–70]. In our study, upregulated HSP levels were found. However, no HSP27 was detected after treatment with XN or XNC, which requires further specific analysis in this issue of different altered HSP levels.

In terms of the downregulated proteins after treatment with XNC, i.e. tubulin (e.g. TUBA1A, TUBA1B, TUBA1C, TUBA3D, TUBB6), myosin (e.g. MYL12A, MAL12B, MYL6, MYL9, MYH7B, MYH9) and various kinases (e.g. CDKs, MAPKs, PAK, PKC) were detected. Tubulin and myosin participate in cell-cell adhesion or cell-matrix adhesion such as tight junction and focal adhesion. A downregulation of these proteins might affect cell-cell contacts, resulting in a reduction of cell growth or cell death. This is in accordance with the enrichment analysis (see summary in S1 and S2 Tables), which indicates a significant overrepresentation of proteins involved in the regulation of cell proliferation and the homotypic cell-cell adhesion. A downregulation of tubulin and myosin by XNC might result in an inhibition of microtubule dynamics. As described by Rathinasamy et al.[71] for the compound griseofulvin and Banerjee et al. [72] for the compound curcumin, a suppression of microtubule dynamics inhibits the proliferation of MCF-7 cells and induces cell apoptosis. This indicates also a possible reason for the proliferative and cytotoxic effect of XNC in our study. Since kinases are involved in cell proliferation, DNA damage checkpoint regulation and pathway repair, they are often deregulated in cancer. Therefore, our analysis suggests XNC also as a potential anticancer agent for targeting various kinases [73–75]. Downregulation of tubulin, myosin and various kinases could possibly be related to the cytotoxic effect of XNC in MCF-7 cell line. However, further experiments that proof this hypothesis are needed. Noteworthy and similar to the treatment with XN, also proteins involved in the type I interferon signaling pathway (ISG15, OAS1, OAS3, IRF9, STAT1, and IFI35) were downregulated in XNC treated cells. This might be an indication for a MCF-7 defense mechanism to the compound treatment with XN and XNC.

To conclude the proteomics analysis, the comparison between the XN and XNC regulated proteomes suggests different modes of action. The strong effect of pure XNC on the growth of MCF-7 compared to XN seems to be related to the downregulation of cell-cell adhesion proteins, i.e. cytoskeletal proteins such as tubulin, myosin and various kinases, and the suppression of microtubule dynamics. In terms of XN, the downregulation of the interferon pathway and JAK/STAT signaling pathway seems to cause cell apoptosis. These findings underscore also the cytotoxic effects of the compounds observed in the cell culture experiments before.

Confirming the proteomics approach with a much higher number of affected proteins, pure XNC shows a stronger effect on the growth of MCF-7 compared to pure XN. The higher effect of the XN -enriched extract XF in the cell culture experiments, including also small amounts of XNC and other minor hop compounds, demonstrates the supporting activity of minor hop compounds. Further, our results demonstrate the potential of pure minor hop compounds to be more active than extracts still containing a bunch of minor compounds as impurities. Concluding, minor hop compounds could also be deployed in a targeted way and added to anticarcinogenic drugs or preventive food additives to enhance their bioactivity.

## Conclusions

In this study, we explored the effect of xanthohumol C (XNC) compared to xanthohumol (XN) and the commercial XN -enriched hop extract Xantho-Flav (XF) on human breast cancer cell line MCF-7 in vitro. All three showed dose-dependent antiproliferative and cytotoxic effects after 2, 4, and 6 days of incubation. Remarkably, XN was less cytotoxic than the XN -enriched extract XF, while the activity of XNC was highest. All three compounds clearly differed in their half maximal inhibitory concentration (IC_50_ after two days XNC: 4.18 *μ*M, XF: 8.84 *μ*M, XN: 12.25 *μ*M). This promises the bioactive potential of minor prenylated hop chalcones for further applications in cancer research and indicates hop as a versatile natural resource of active minor compounds with potential anticarcinogenic effects. The results demonstrate a higher anticarcinogenic or chemopreventive potential of the studied hop compounds compared to other phenolic compounds such as resveratrol (IC_50_: 100μM) or curcumin (IC_50_: 35μM) [55]. The study indicates the need of highly pure minor compounds to clearly associate certain effects with single compounds.

Further, the quantitative proteomics approach provided a comprehensive view on the proteome composition of XN and XNC treated MCF-7 cells. Based on the differences in protein expression, this approach gave insights in molecular mechanisms and possible target structures, which indicate different modes of action between XN and XNC treated cells. XN upregulated proteins involved in cell cycles and DNA replication and downregulated proteins of the type I interferon signaling pathway. In contrast, upregulated proteins after XNC treatment were responses to ER stress and downregulated proteins were involved in cell-cell adhesion. In addition, XNC possibly modulated kinase expression. The applied proteomics approach has the potential to be a state-of-the-art technique for bioactivity screenings of promising bioactive minor hop compounds. The study emphasizes the potential of liquid-liquid chromatography to isolate highly pure natural compounds from complex mixtures in sufficient amounts, which is needed to analyze the bioactivity of each compound specifically. By this, previously unavailable natural minor compounds could be provided in high purity. In addition, XNC might also be a promising preventive or anticarcinogenic target for the use together with current anticarcinogenic drugs to enhance antiproliferative and cytotoxic effects.

## Supporting information

S1 Fig DE proteins after xanthohumol treatment. Differentially expressed proteins in xanthohumol treated MCF-7 with their respective molecular function. Proteins are sorted by their differences in expression compared to control cells, showing only proteins with 2.5 fold up- or downregulation based on log2 transformed LFQ intensities.
(TIF)

S2 Fig DE proteins after xanthohumol C treatment. Differentially expressed proteins in xanthohumol C treated MCF-7 with their respective molecular function. Proteins are sorted by their differences in expression compared to control cells, showing only proteins with 2.5 fold up- or downregulation based on log2 transformed LFQ intensities.
(TIF)

S3 Fig Functional signal networks after xanthohumol treatment. Functional signal networks of up- (a) and downregulated (b) proteins in xanthohumol treated MCF-7. In (b), proteins were marked that are involved in the type I interferon signaling pathway.
(TIF)

S4 Fig Functional signal networks after xanthohumol C treatment. Functional signal networks of up- (a) and downregulated (b) DE proteins in xanthohumol C treated MCF-7. (a): Heat shock proteins were marked in grey. (b): In blue kinases were marked, in grey proteins involved in tubulin, and in black proteins implemented in the type I interferon signaling pathway.
(TIF)

S1 Table Enrichment analysis of upregulated proteins after xanthohumol C treatment. Enrichment analysis of upregulated proteins in xanthohumol C treated MCF-7 implemented in the web tool GOrilla. Gene ontology terms, description of the molecular function in which enriched proteins were involved, p-values, false discovery rates (FDR), and enrichment factors are shown.
(PDF)

S2 Table Enrichment analysis of downregulated proteins after xanthohumol C treatment. Enrichment analysis of downregulated proteins in xanthohumol C treated MCF-7 implemented in the web tool GOrilla. Gene ontology terms, description of the molecular function in which enriched proteins were involved, p-values, false discovery rates (FDR), and enrichment factors are shown.
(PDF)

S1 File All identified peptides as output from MaxQuant. (XLSX)

S2 File All identified protein groups as output from MaxQuant. (XLSX)

S3 File MaxQuant configuration. (XML)

S4 File Quality control report performed with raw data from MaxQuant. (PDF)

S5 File All quantified proteins. (XLSX)

S6 File Downregulated DE proteins after treatment with Xanthohumol C. (XLSX)

S7 File Upregulated DE proteins after treatment with Xanthohumol C. (XLSX)

S8 File Downregulated DE proteins after treatment with Xanthohumol. (XLSX)

S9 File Upregulated DE proteins after treatment with Xanthohumol. (XLSX)
