# Supplementary figures and images for: Analyzing bioactive effects of the minor hop compound xanthohumol C on human breast cancer cells using quantitative proteomics
Source: PLoS One. 2019 Mar 15;14(3):e0213469. doi: 10.1371/journal.pone.0213469 (PMC6420031; doi:10.1371/journal.pone.0213469)

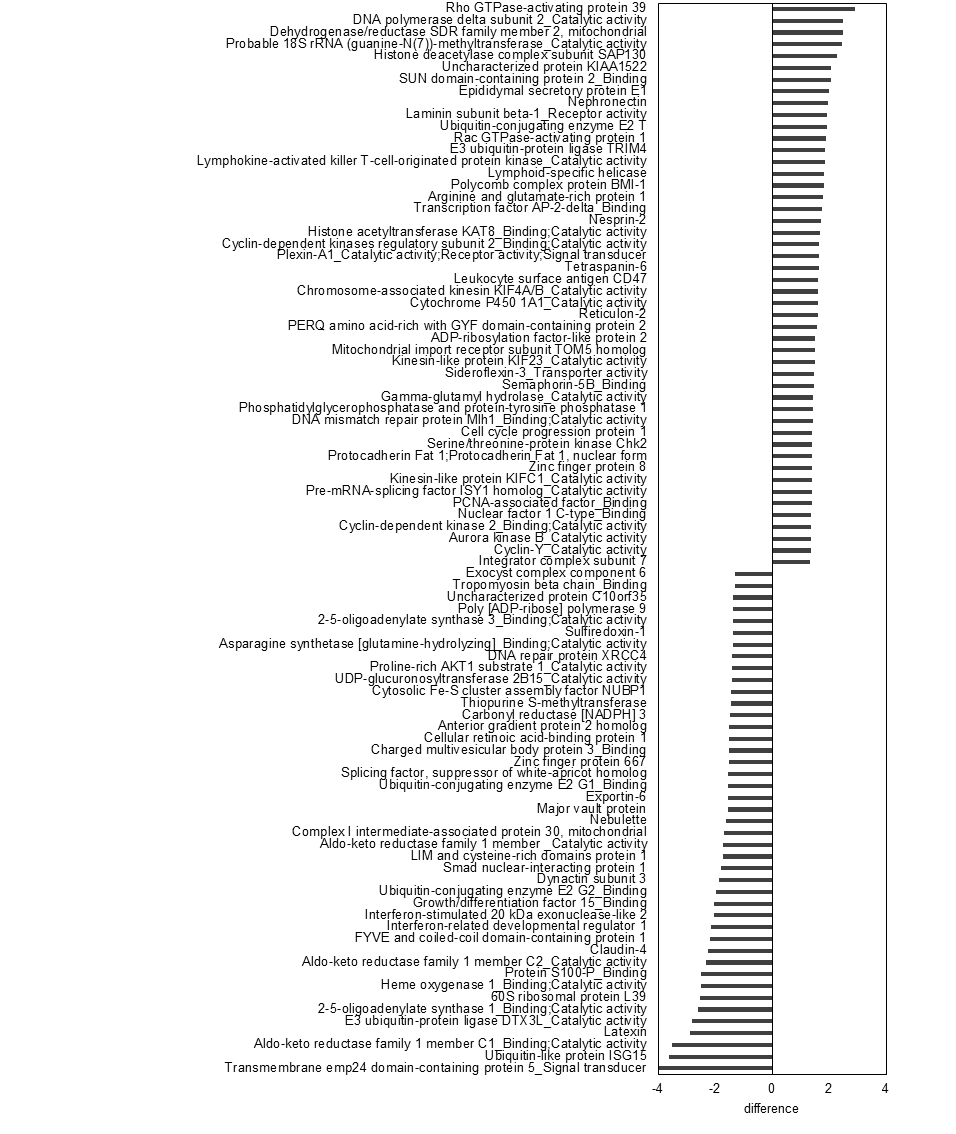

Supplement: S1 Fig — Differentially expressed proteins in xanthohumol treated MCF-7 with their respective molecular function. Proteins are sorted by their differences in expression compared to control cells, showing only proteins with 2.5 fold up- or downregulation based on log2 transformed LFQ intensities. (TIF) [file pone.0213469.s001.tif]

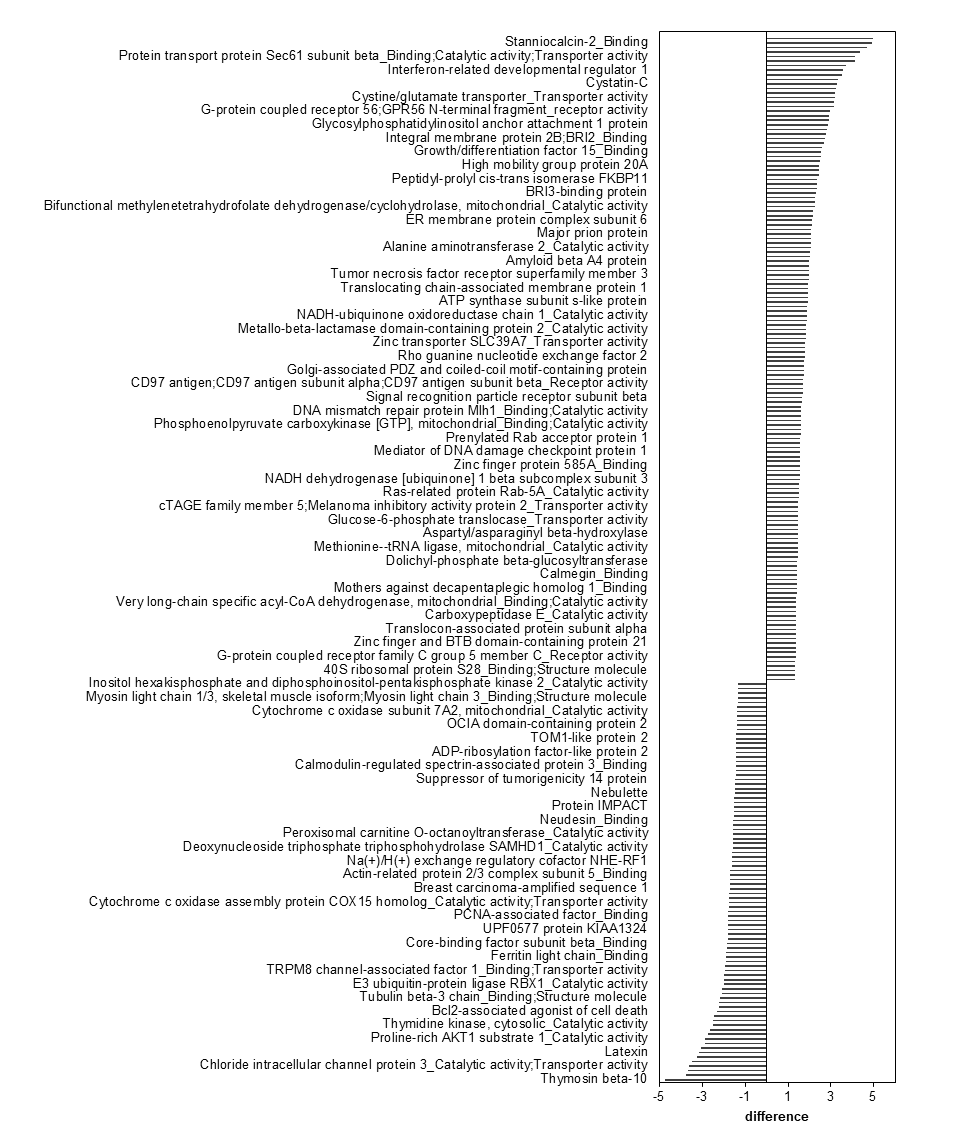

Supplement: S2 Fig — Differentially expressed proteins in xanthohumol C treated MCF-7 with their respective molecular function. Proteins are sorted by their differences in expression compared to control cells, showing only proteins with 2.5 fold up- or downregulation based on log2 transformed LFQ intensities. (TIF) [file pone.0213469.s002.tif]

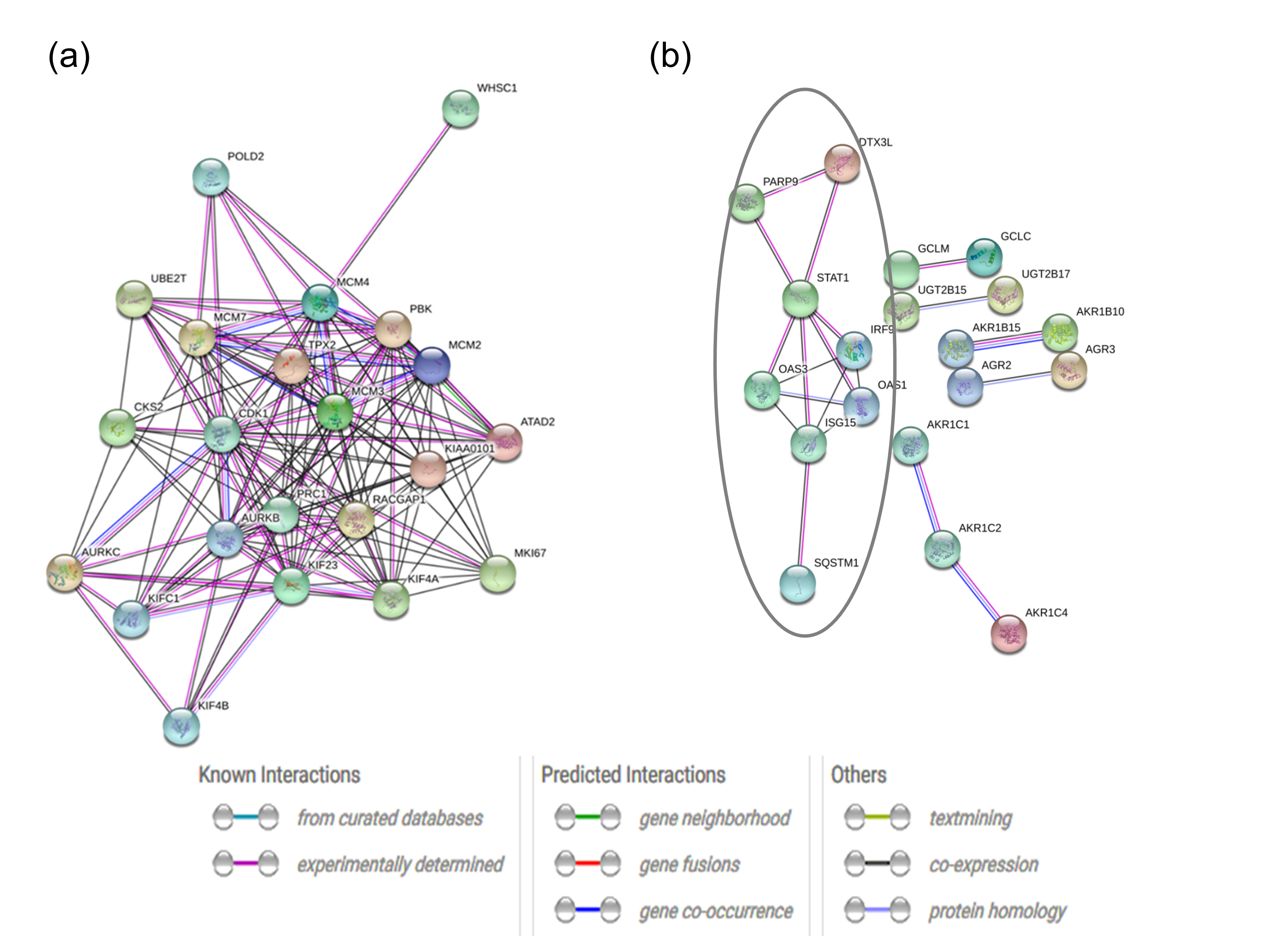

Supplement: S3 Fig — Functional signal networks of up- (a) and downregulated (b) proteins in xanthohumol treated MCF-7. In (b), proteins were marked that are involved in the type I interferon signaling pathway. (TIF) [file pone.0213469.s003.TIF]

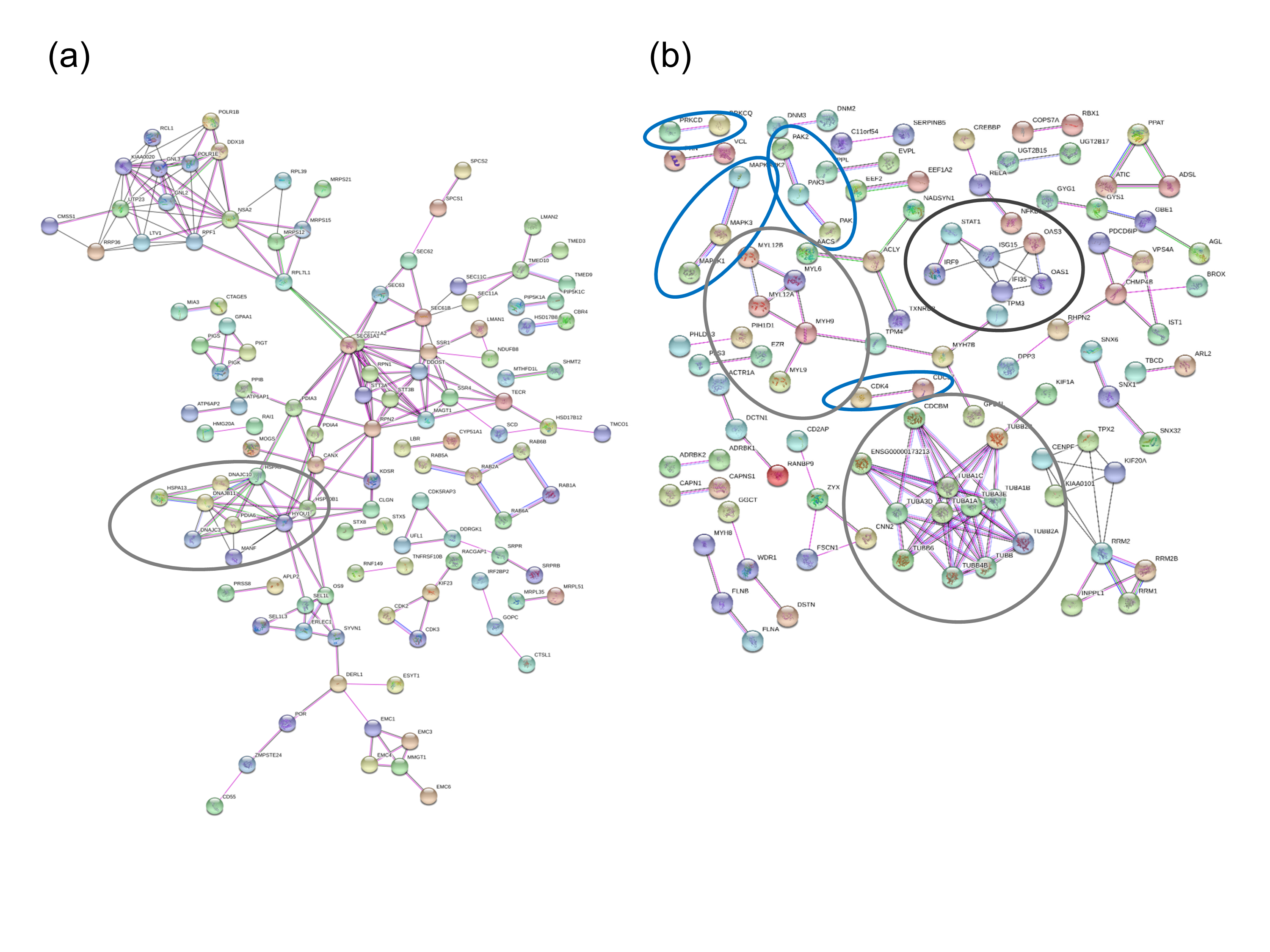

Supplement: S4 Fig — Functional signal networks of up- (a) and downregulated (b) DE proteins in xanthohumol C treated MCF-7. (a): Heat shock proteins were marked in grey. (b): In blue kinases were marked, in grey proteins involved in tubulin, and in black proteins implemented in the type I interferon signaling pathway. (TIF) [file pone.0213469.s004.TIF]
