# Supplementary material for: Analyzing bioactive effects of the minor hop compound xanthohumol C on human breast cancer cells using quantitative proteomics
Source: PLoS One. 2019 Mar 15;14(3):e0213469. doi: 10.1371/journal.pone.0213469 (PMC6420031; doi:10.1371/journal.pone.0213469)
Supplement: S1 Table — Enrichment analysis of upregulated proteins in xanthohumol C treated MCF-7 implemented in the web tool GOrilla. Gene ontology terms, description of the molecular function in which enriched proteins were involved, p-values, false discovery rates (FDR), and enrichment factors are shown. (PDF) [file pone.0213469.s005.pdf]

S1 Table. Enrichment analysis of upregulated proteins after xanthohumol C treatment. Enrichment analysis of upregulated proteins in xanthohumol C treated MCF-7 implemented in the web tool GOrilla. Gene ontology terms, description of the molecular function in which enriched proteins were involved, p-values, false discovery rates (FDR), and enrichment factors are shown.

GO term	description	p-value	FDR q-value	enrichment (N, B, n, b)
GO:0050896	response to stimulus	6.90E-08	2.54E-04	1.47 (398,124,185,85)
GO:1901700	response to oxygen-containing compound	1.26E-07	2.31E-04	2.55 (398,35,116,26)
GO:0010033	response to organic substance	4.51E-07	5.54E-04	1.91 (398,63,139,42)
GO:0002376	immune system process	6.08E-07	5.61E-04	1.84 (398,51,170,40)
GO:0043562	cellular response to nitrogen levels	2.81E-06	2.07E-03	74.62 (398,4,4,3)
GO:0006995	cellular response to nitrogen starvation	2.81E-06	1.73E-03	74.62 (398,4,4,3)
GO:0071310	cellular response to organic substance	3.45E-06	1.82E-03	3.10 (398,24,91,17)
GO:0009605	response to external stimulus	4.43E-06	2.04E-03	1.82 (398,39,185,33)
GO:0014070	response to organic cyclic compound	6.01E-06	2.46E-03	2.73 (398,23,114,18)
GO:0042221	response to chemical	6.83E-06	2.52E-03	1.73 (398,78,139,47)
GO:0009719	response to endogenous stimulus	7.27E-06	2.44E-03	2.63 (398,25,115,19)
GO:0048518	positive regulation of biological process	9.03E-06	2.77E-03	1.45 (398,105,185,71)
GO:0000422	autophagy of mitochondrion	1.03E-05	2.91E-03	59.70 (398,5,4,3)
GO:0061726	mitochondrion disassembly	1.03E-05	2.71E-03	59.70 (398,5,4,3)
