# Supplementary material for: Analyzing bioactive effects of the minor hop compound xanthohumol C on human breast cancer cells using quantitative proteomics
Source: PLoS One. 2019 Mar 15;14(3):e0213469. doi: 10.1371/journal.pone.0213469 (PMC6420031; doi:10.1371/journal.pone.0213469)
Supplement: S2 Table — Enrichment analysis of downregulated proteins in xanthohumol C treated MCF-7 implemented in the web tool GOrilla. Gene ontology terms, description of the molecular function in which enriched proteins were involved, p-values, false discovery rates (FDR), and enrichment factors are shown. (PDF) [file pone.0213469.s006.pdf]

S2 Table. Enrichment analysis of downregulated proteins after xanthohumol C treatment. Enrichment analysis of downregulated proteins in xanthohumol C treated MCF-7 implemented in the web tool GOrilla. Gene ontology terms, description of the molecular function in which enriched proteins were involved, p-values, false discovery rates (FDR), and enrichment factors are shown.

**GO term**	**description**	**p-value**	**FDR q-value**	**enrichment (N, B, n, b)**
GO:0042221	response to chemical	1.23E-07	5.95E-04	1.50 (422,91,222,72)
GO:0010033	response to organic substance	1.28E-07	3.10E-04	1.57 (422,74,222,61)
GO:0008285	negative regulation of cell proliferation	2.39E-06	3.85E-03	2.62 (422,25,129,20)
GO:0048856	anatomical structure development	8.03E-06	9.70E-03	1.43 (422,95,220,71)
GO:0098771	inorganic ion homeostasis	9.77E-06	9.45E-03	2.29 (422,16,184,16)
GO:0050801	ion homeostasis	9.77E-06	7.88E-03	2.29 (422,16,184,16)
GO:0006366	transcription from RNA polymerase II promoter	1.15E-05	7.97E-03	1.94 (422,20,217,20)
GO:0042127	regulation of cell proliferation	1.50E-05	9.06E-03	2.13 (422,43,129,28)
GO:0006873	cellular ion homeostasis	2.23E-05	1.20E-02	2.29 (422,15,184,15)
GO:0055080	cation homeostasis	2.23E-05	1.08E-02	2.29 (422,15,184,15)
GO:0009719	response to endogenous stimulus	2.36E-05	1.04E-02	1.76 (422,36,207,31)
GO:0070887	cellular response to chemical stimulus	2.87E-05	1.16E-02	1.54 (422,58,222,47)
GO:0071310	cellular response to organic substance	2.97E-05	1.10E-02	1.59 (422,49,222,41)
GO:0032501	multicellular organismal process	3.03E-05	1.05E-02	1.43 (422,86,223,65)
GO:0007166	cell surface receptor signaling pathway	3.36E-05	1.08E-02	1.48 (422,61,239,51)
GO:0002376	immune system process	4.51E-05	1.36E-02	1.43 (422,83,224,63)
GO:0051239	regulation of multicellular organismal process	4.69E-05	1.33E-02	1.42 (422,86,225,65)
GO:0055082	cellular chemical homeostasis	5.01E-05	1.35E-02	2.17 (422,18,184,17)
GO:0030003	cellular cation homeostasis	5.12E-05	1.30E-02	2.29 (422,14,184,14)

GO term	description	p-value	FDR q-value	enrichment (N, B, n, b)
GO:0071495	cellular response to endogenous stimulus	6.18E-05	1.49E-02	1.82 (422,28,207,25)
GO:0050896	response to stimulus	7.53E-05	1.73E-02	1.32 (422,143,208,93)
GO:0007165	signal transduction	8.05E-05	1.77E-02	1.34 (422,128,212,86)
GO:0019725	cellular homeostasis	1.40E-04	2.95E-02	2.06 (422,20,184,18)
GO:0006937	regulation of muscle contraction	1.64E-04	3.31E-02	4.10 (422,7,103,7)
GO:0001775	cell activation	1.71E-04	3.30E-02	1.62 (422,48,201,37)
GO:0007568	aging	1.74E-04	3.23E-02	2.01 (422,15,210,15)
GO:1901700	response to oxygen-containing compound	2.56E-04	4.58E-02	1.58 (422,49,207,38)
GO:0055065	metal ion homeostasis	2.61E-04	4.51E-02	2.29 (422,12,184,12)
GO:0048878	chemical homeostasis	2.90E-04	4.84E-02	1.82 (422,27,197,23)
GO:0034109	homotypic cell-cell adhesion	3.23E-04	5.20E-02	3.27 (422,10,116,9)
