# Supplementary material for: Analyzing bioactive effects of the minor hop compound xanthohumol C on human breast cancer cells using quantitative proteomics
Source: PLoS One. 2019 Mar 15;14(3):e0213469. doi: 10.1371/journal.pone.0213469 (PMC6420031; doi:10.1371/journal.pone.0213469)

Performance overview

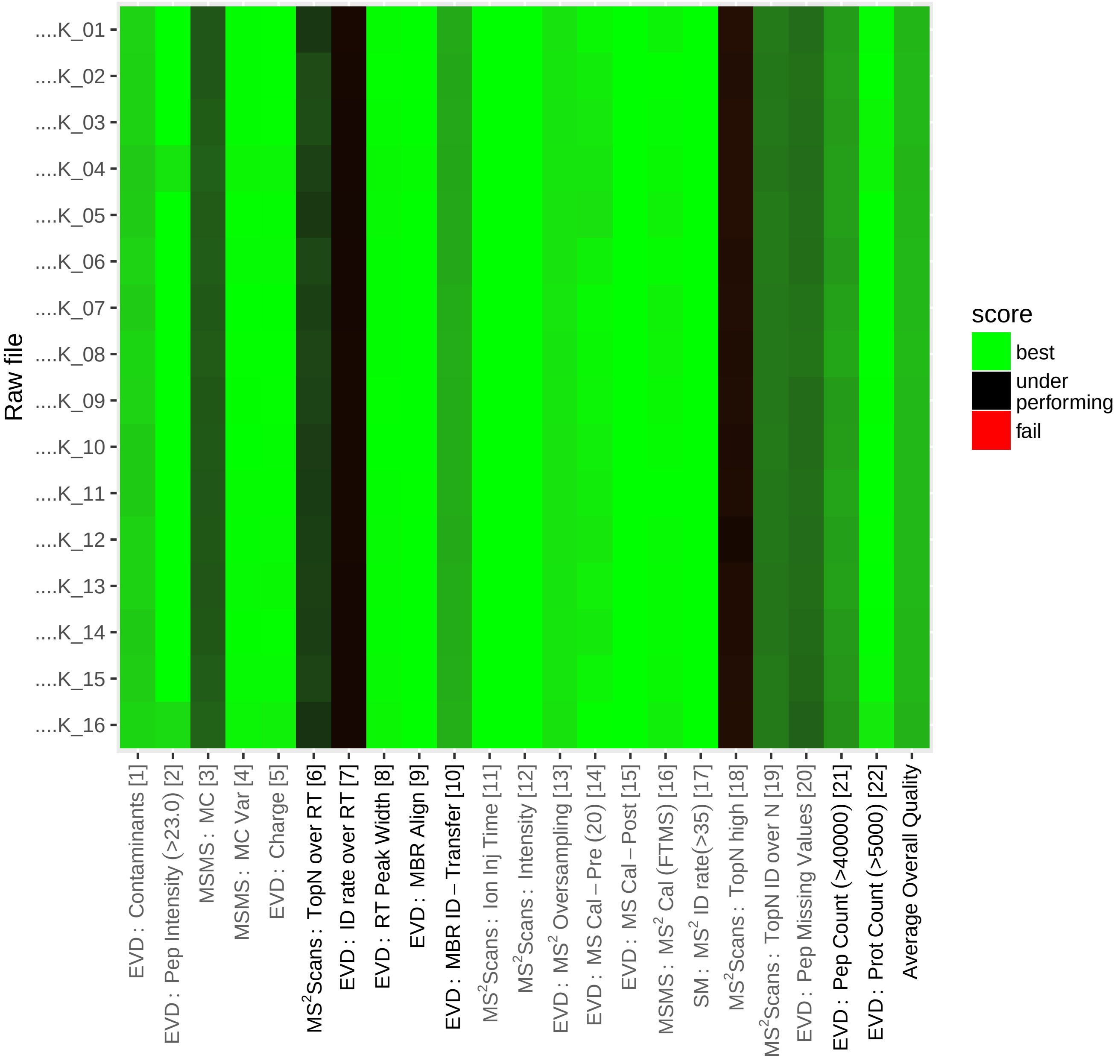

Mapping of Raw files to their short names
Mapping source: file (user−defined)
(automatic shortening of names was not sufficiently short − see 'best effort')

original	short name	best effort
BBM_049_P057_01_HEK_01	....K_01	....K_01
BBM_049_P057_01_HEK_02	....K_02	....K_02
BBM_049_P057_01_HEK_03	....K_03	....K_03
BBM_049_P057_01_HEK_04	....K_04	....K_04
BBM_049_P057_01_HEK_05	....K_05	....K_05
BBM_049_P057_01_HEK_06	....K_06	....K_06
BBM_049_P057_01_HEK_07	....K_07	....K_07
BBM_049_P057_01_HEK_08	....K_08	....K_08
BBM_049_P057_01_HEK_09	....K_09	....K_09
BBM_049_P057_01_HEK_10	....K_10	....K_10
BBM_049_P057_01_HEK_11	....K_11	....K_11
BBM_049_P057_01_HEK_12	....K_12	....K_12
BBM_049_P057_01_HEK_13	....K_13	....K_13
BBM_049_P057_01_HEK_14	....K_14	....K_14
BBM_049_P057_01_HEK_15	....K_15	....K_15
BBM_049_P057_01_HEK_16	....K_16	....K_16

Calculate peak properties	False	MS/MS deisotoping (Unknown)	False
Combined folder location		MS/MS dependent losses (FTMS..	True
Da interval. (FTMS)	100	MS/MS dependent losses (ITMS..	True
Da interval. (ITMS)	100	MS/MS dependent losses (TOF)	True
Da interval. (TOF)	100	MS/MS dependent losses (Unkn..	True
Da interval. (Unknown)	100	MS/MS higher charges (FTMS)	True
Date of writing	11/04/2017 06:18:13	MS/MS higher charges (ITMS)	True
Decoy mode	revert	MS/MS higher charges (TOF)	True
Disable MD5	False	MS/MS higher charges (Unknow..	True
Discard unmodified counterpa..	True	MS/MS recalibration (FTMS)	False
Find dependent peptides	False	MS/MS recalibration (ITMS)	False
Fixed andromeda index folder		MS/MS recalibration (TOF)	False
Fixed modifications	Carbamidomethyl (C)	MS/MS recalibration (Unknown..	False
iBAQ	True	MS/MS tol. (FTMS)	20 ppm
iBAQ log fit	True	MS/MS tol. (ITMS)	0.5 Da
Include contaminants	True	MS/MS tol. (TOF)	40 ppm
Label min. ratio count	2	MS/MS tol. (Unknown)	0.5 Da
Machine name	WINCOMPUTING1	MS/MS water loss (FTMS)	True
Main search max. combination..	200	MS/MS water loss (ITMS)	True
Match between runs	True	MS/MS water loss (TOF)	True
Matching time window [min]	0.7	MS/MS water loss (Unknown)	True
Match unidentified features	False	Peptides used for protein qu..	Razor
Max mods in site table	3	Protein FDR	0.01
Max. peptide length for unsp..	25	PSM FDR	0.01
Max. peptide mass [Da]	4600	Razor protein FDR	True
Min. delta score for modifie..	6	Require MS/MS for LFQ compar..	True
Min. delta score for unmodif..	0	Second peptides	True
Min. peptide Length	7	Separate LFQ in parameter gr..	False
Min. peptide length for unsp..	8	Site FDR	0.01
Min. peptides	1	Site tables	Oxidation (M)Sites.txt<br>Phospho (STY)Sites.txt
Min. razor peptides	1	Stabilize large LFQ ratios	True
Min. score for modified pept..	40	Temporary folder	
Min. score for unmodified pe..	0	Top MS/MS peaks per Da inter..	12
Min. unique peptides	0	Top MS/MS peaks per Da inter..	8
Modifications included in pr..	Oxidation (M)<br>Acetyl (Protein N−term)	Top MS/MS peaks per Da inter..	10
MS/MS ammonia loss (FTMS)	True	Top MS/MS peaks per Da inter..	8
MS/MS ammonia loss (ITMS)	True	Use delta score	False
MS/MS ammonia loss (TOF)	True	Use Normalized Ratios For Oc..	True
MS/MS ammonia loss (Unknown)	True	Use only unmodified peptides..	True
MS/MS deisotoping (FTMS)	True	User name	juergen.behr
MS/MS deisotoping (ITMS)	False	Version	1.5.8.3
MS/MS deisotoping (TOF)	True	Write allPeptides table	True
MS/MS deisotoping tolerance	7	Write ms2Scans table	True

PG: PCA of 'raw intensity'

(excludes contaminants)

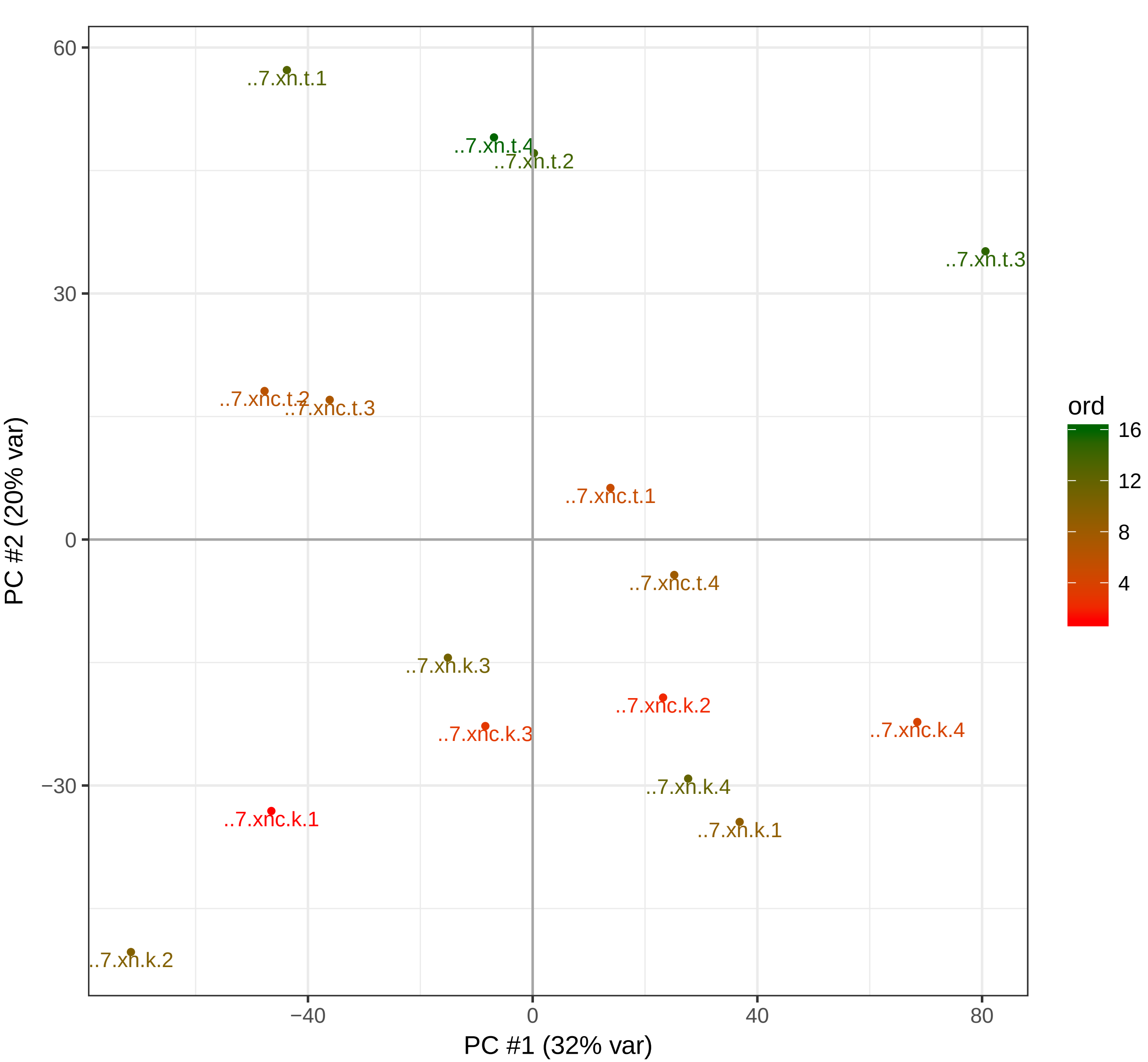

PG: PCA of 'lfq intensity'

(excludes contaminants)

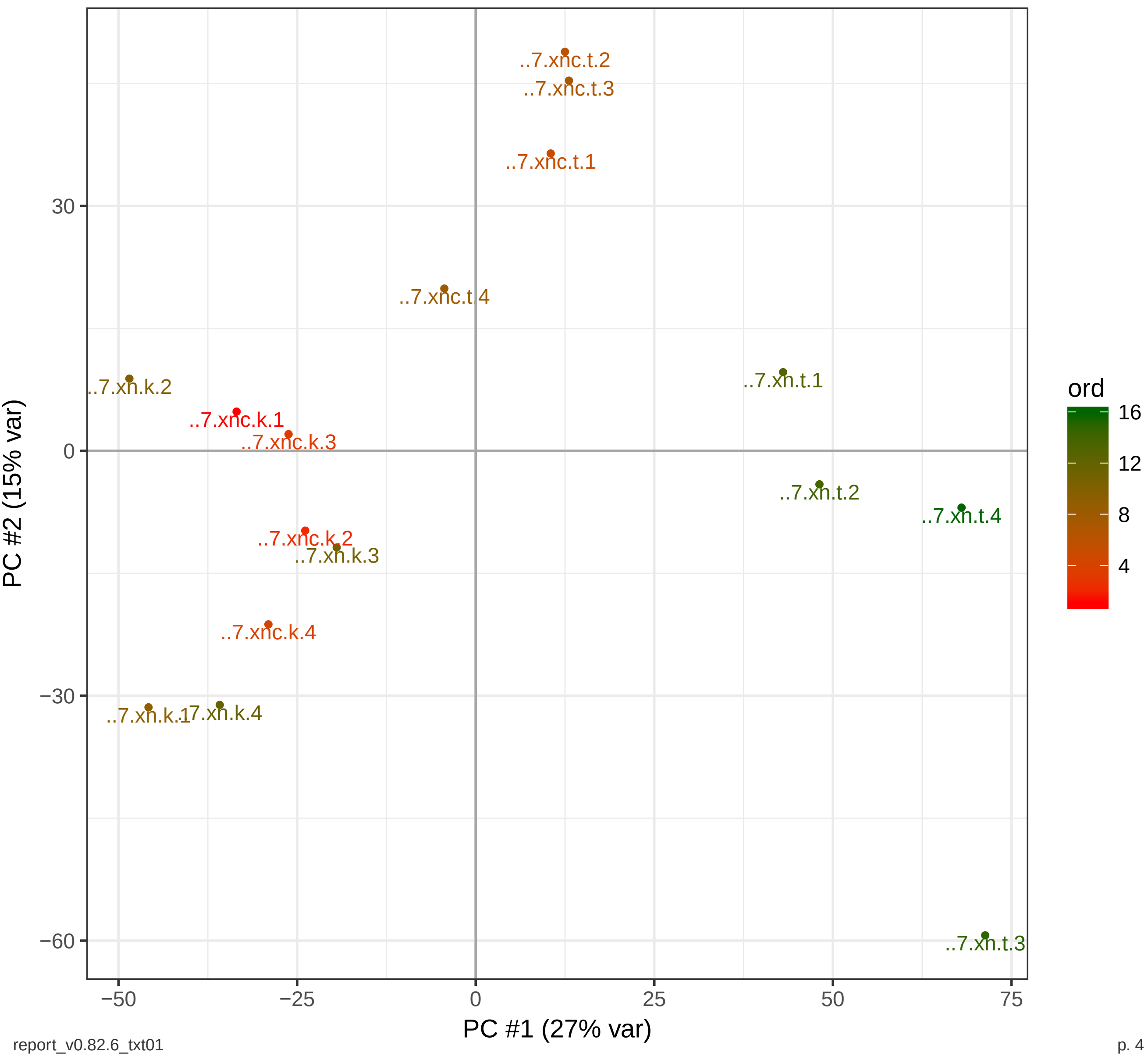

EVD: Top5 Contaminants per Raw file

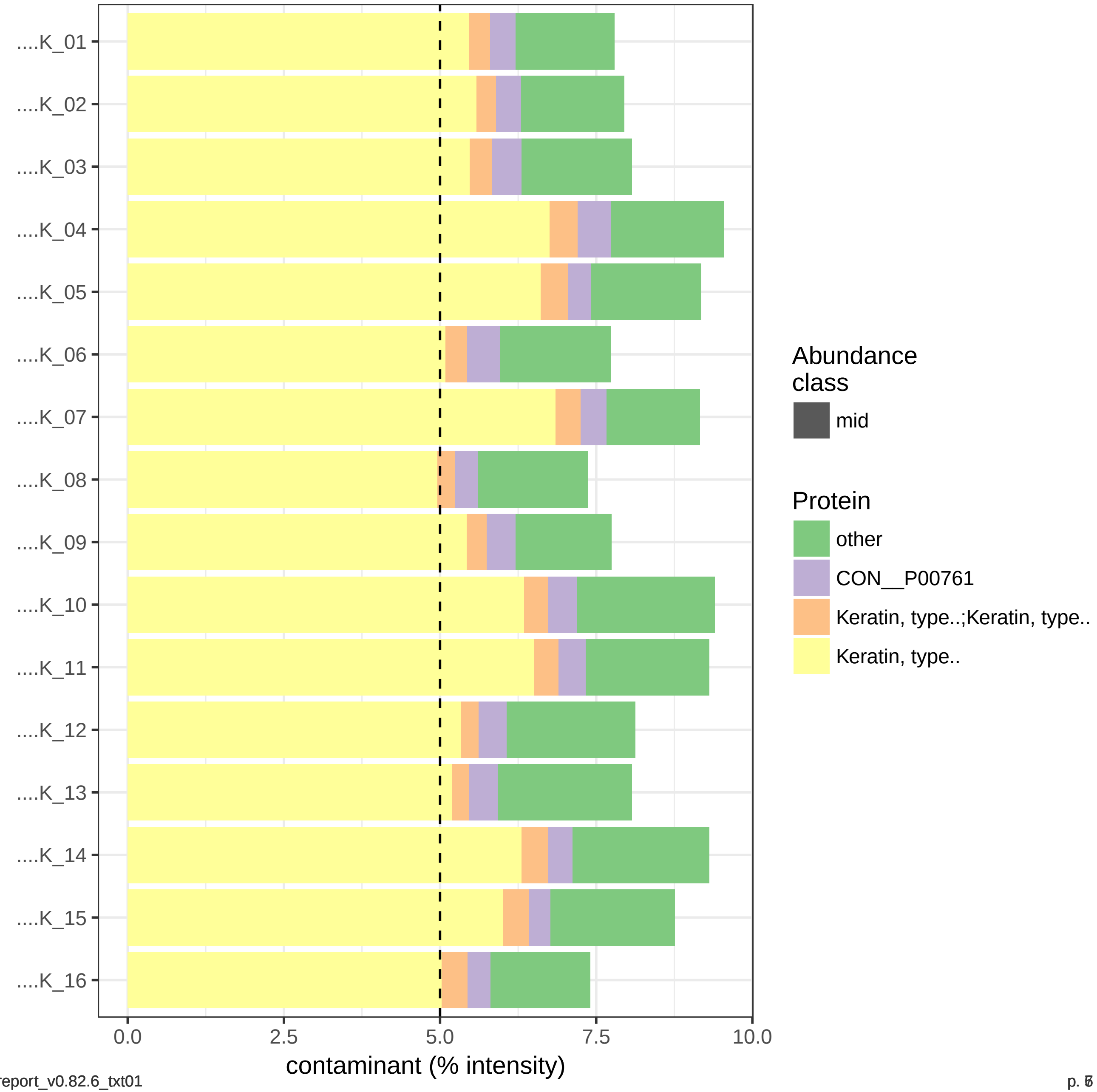

EVD: peptide intensity distribution

RSD 1.3% (expected < 5%)

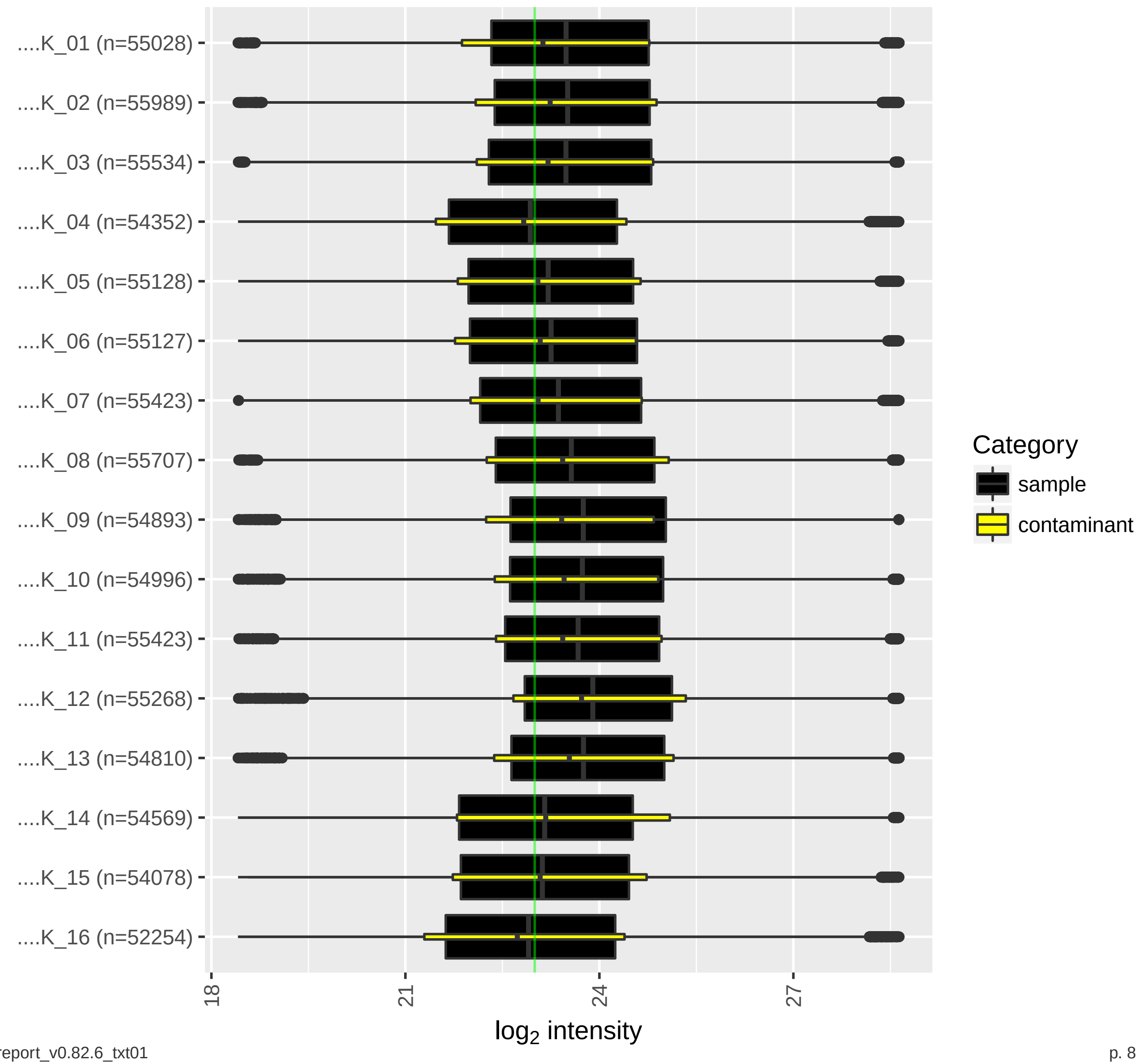

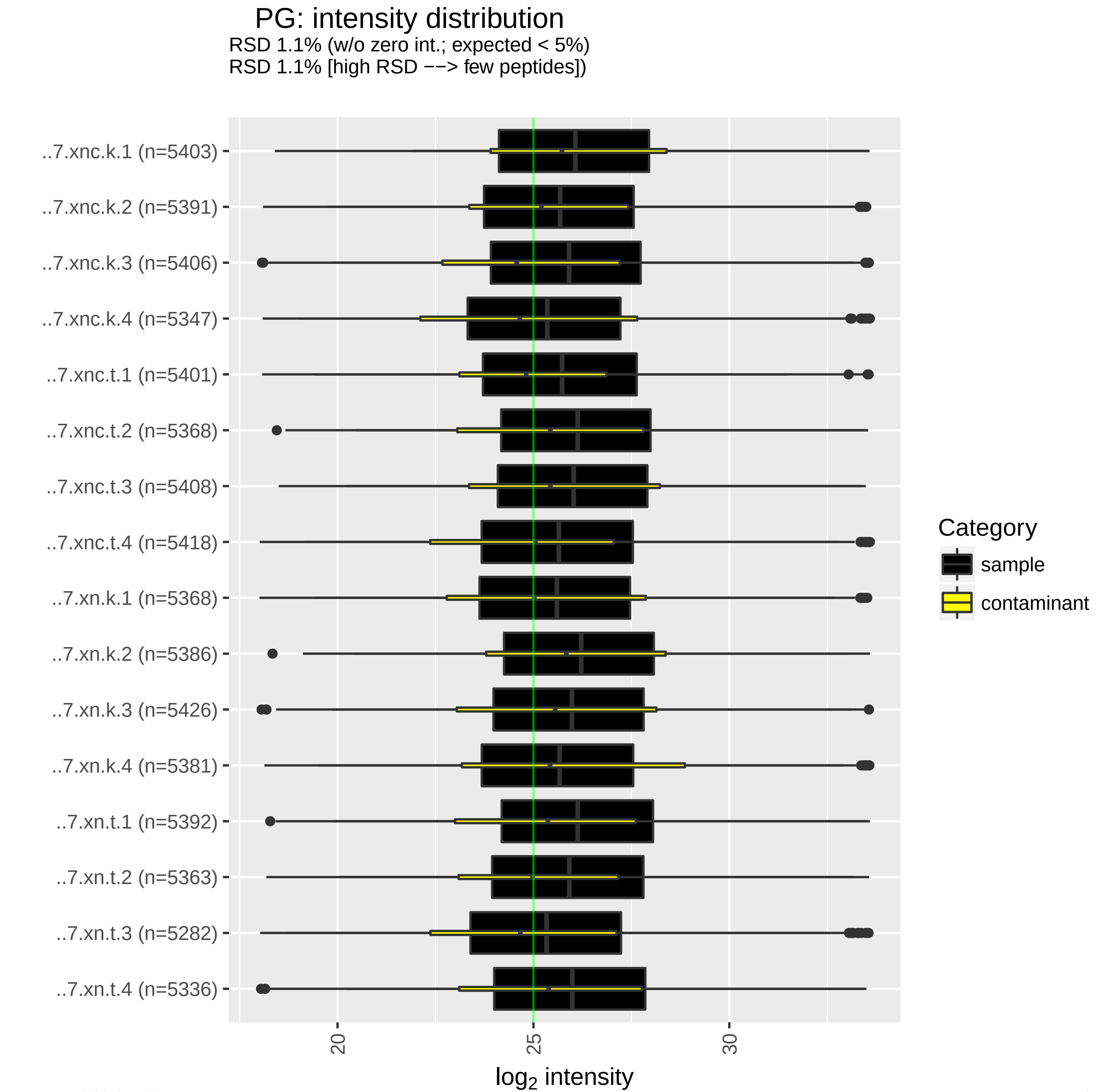

PG: intensity distribution
RSD 1.1% (w/o zero int.; expected < 5%)
RSD 1.1% [high RSD −−> few peptides])
..7.xnc.k.1 (n=5403)
..7.xnc.k.2 (n=5391)
..7.xnc.k.3 (n=5406)
..7.xnc.k.4 (n=5347)
..7.xnc.t.1 (n=5401)
..7.xnc.t.2 (n=5368)
..7.xnc.t.3 (n=5408)
..7.xnc.t.4 (n=5418)
..7.xn.k.1 (n=5368)
..7.xn.k.2 (n=5386)
..7.xn.k.3 (n=5426)
..7.xn.k.4 (n=5381)
..7.xn.t.1 (n=5392)
..7.xn.t.2 (n=5363)
..7.xn.t.3 (n=5282)
..7.xn.t.4 (n=5336)
20
25
30
log2 intensity
Category
sample
contaminant

PG: LFQ intensity distribution

RSD 0.2% (w/o zero int.; expected < 5%)
RSD 0.2% [high RSD −−> few peptides])

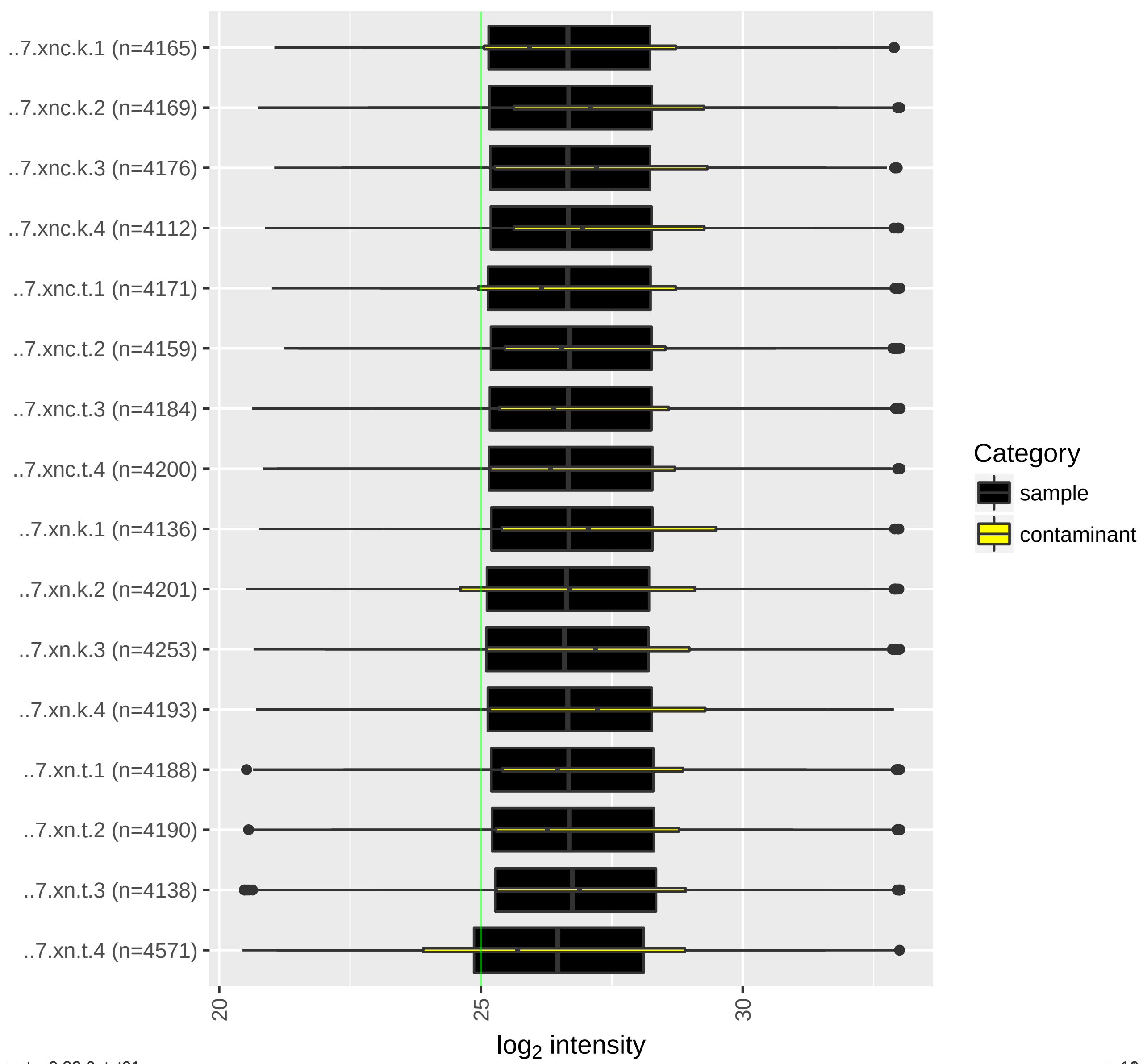

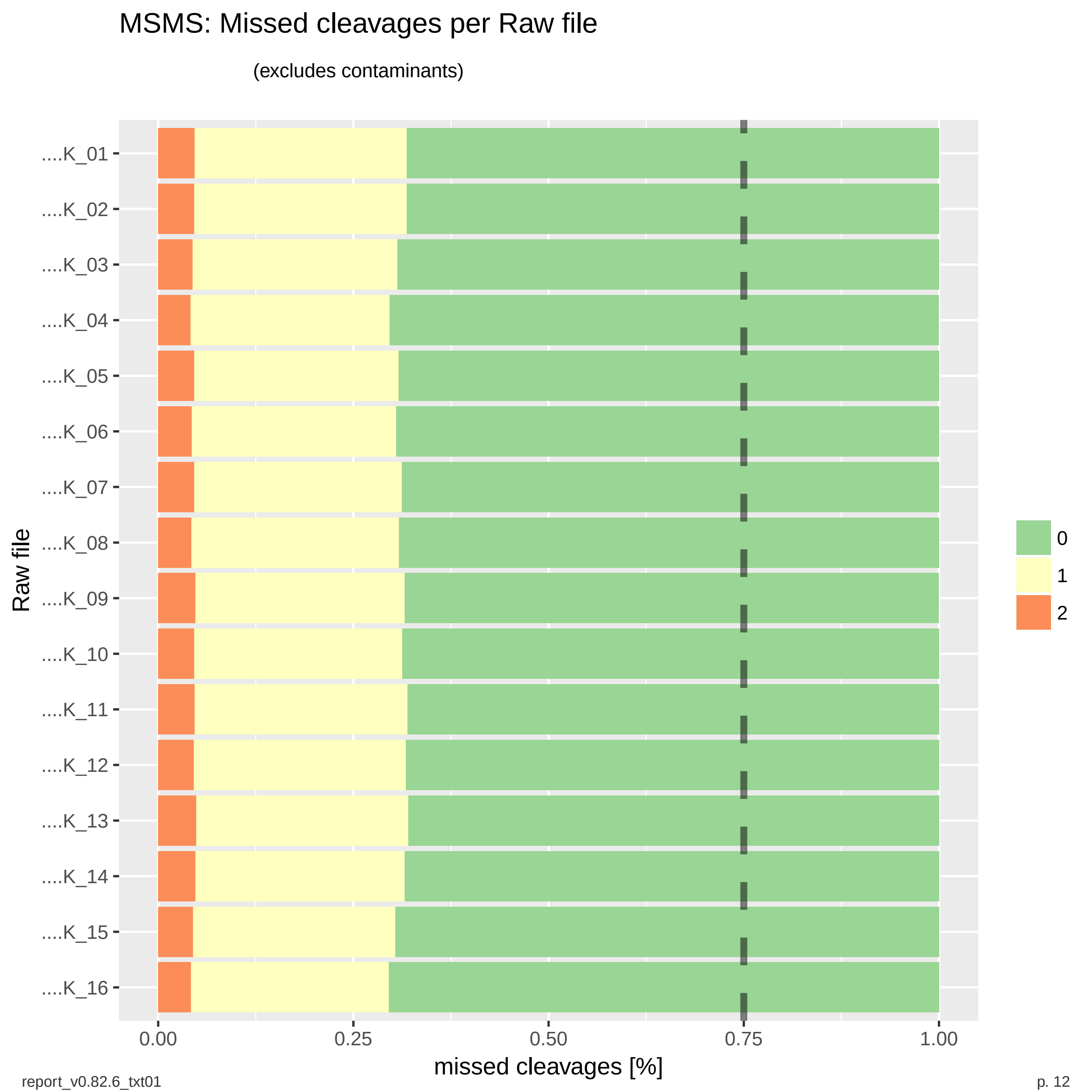
MSMS: Missed cleavages per Raw file
(excludes contaminants)
Raw file
....K_01
....K_02
....K_03
....K_04
....K_05
....K_06
....K_07
....K_08
....K_09
....K_10
....K_11
....K_12
....K_13
....K_14
....K_15
....K_16
0.00
0.25
0.50
0.75
1.00
missed cleavages [%]
0
1
2

EVD: charge distribution

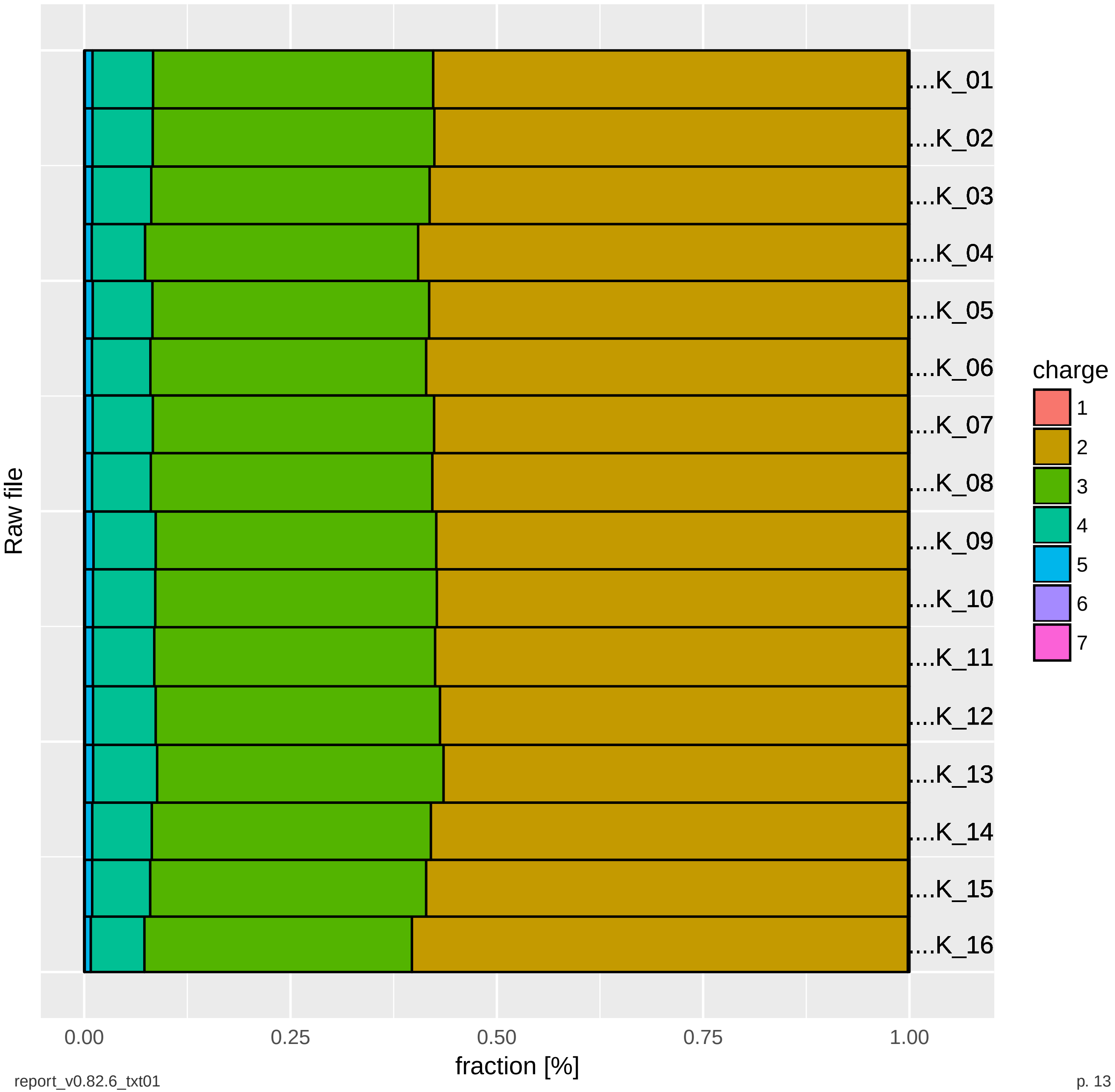

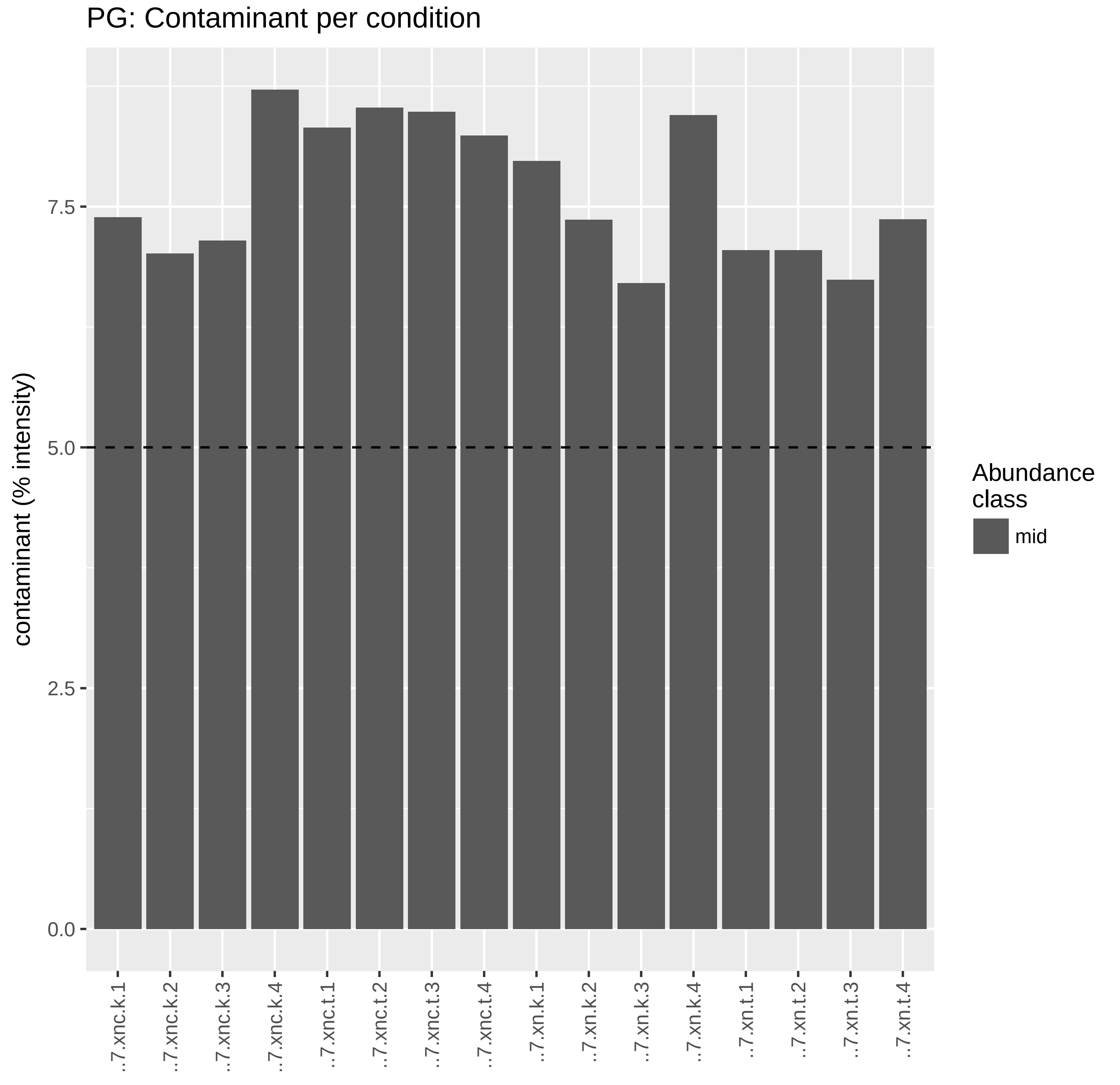
PG: Contaminant per condition
contaminant (% intensity)
0.0
2.5
5.0
7.5
..7.xnc.k.1
..7.xnc.k.2
..7.xnc.k.3
..7.xnc.k.4
..7.xnc.t.1
..7.xnc.t.2
..7.xnc.t.3
..7.xnc.t.4
..7.xn.k.1
..7.xn.k.2
..7.xn.k.3
..7.xn.k.4
..7.xn.t.1
..7.xn.t.2
..7.xn.t.3
..7.xn.t.4
Abundance class
mid

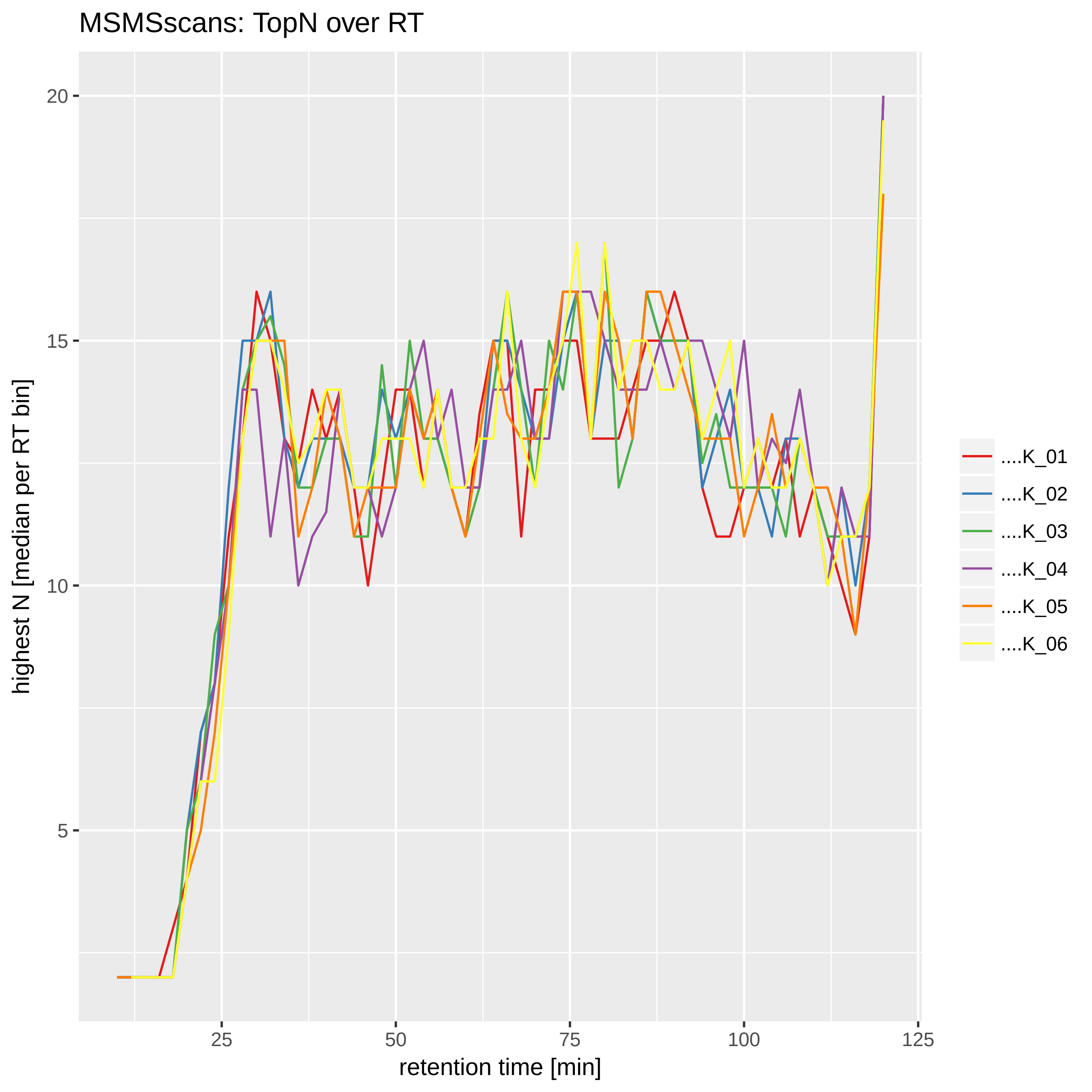
MSMSscans: TopN over RT
highest N [median per RT bin]
20
15
10
5
25
50
75
100
125
retention time [min]
....K_01
....K_02
....K_03
....K_04
....K_05
....K_06

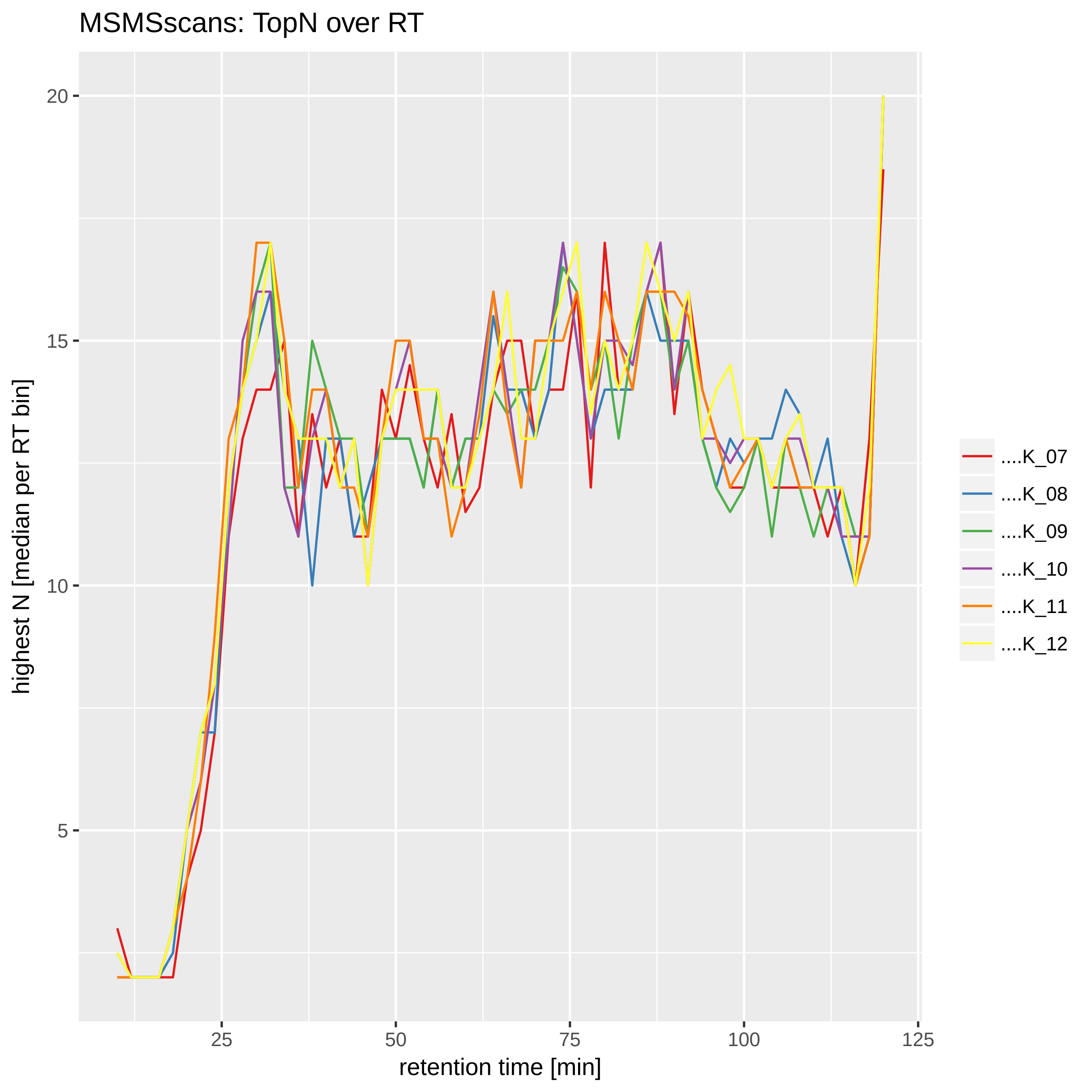

MSMSscans: TopN over RT
highest N [median per RT bin]
retention time [min]
20
15
10
5
25
50
75
100
125
....K_07
....K_08
....K_09
....K_10
....K_11
....K_12

MSMSscans: TopN over RT

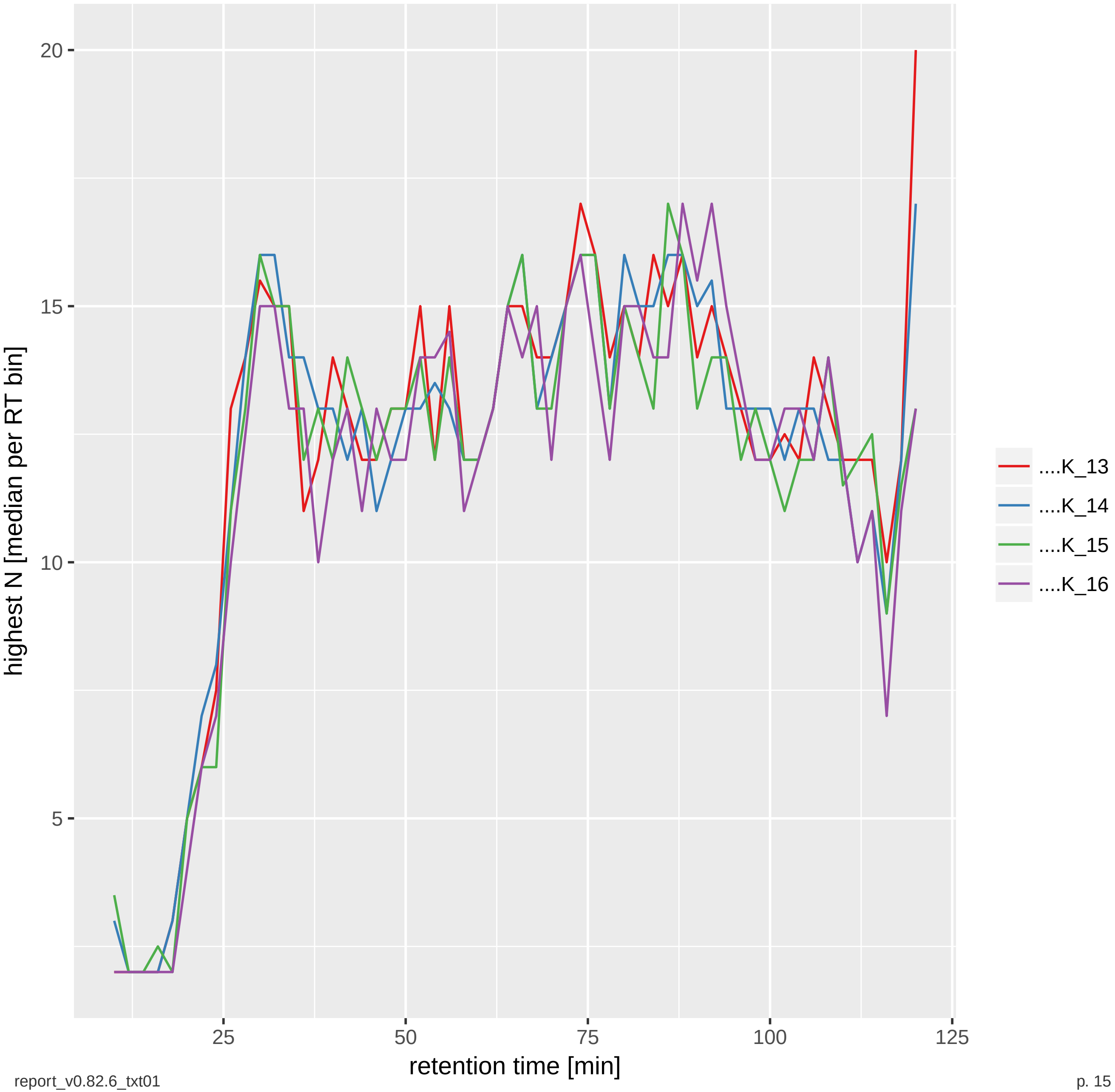

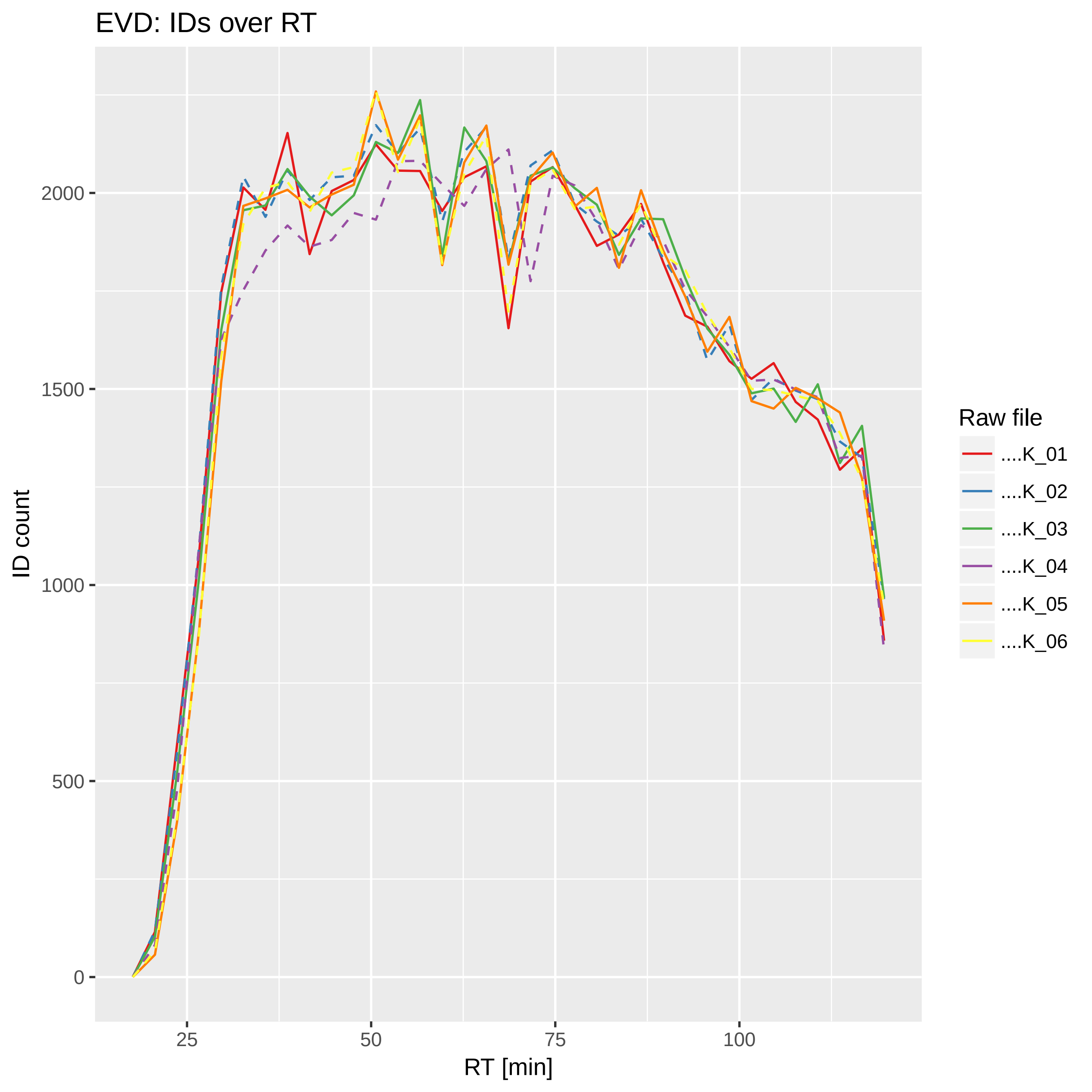

EVD: IDs over RT
ID count
0
500
1000
1500
2000
25
50
75
100
RT [min]
Raw file
....K_01
....K_02
....K_03
....K_04
....K_05
....K_06

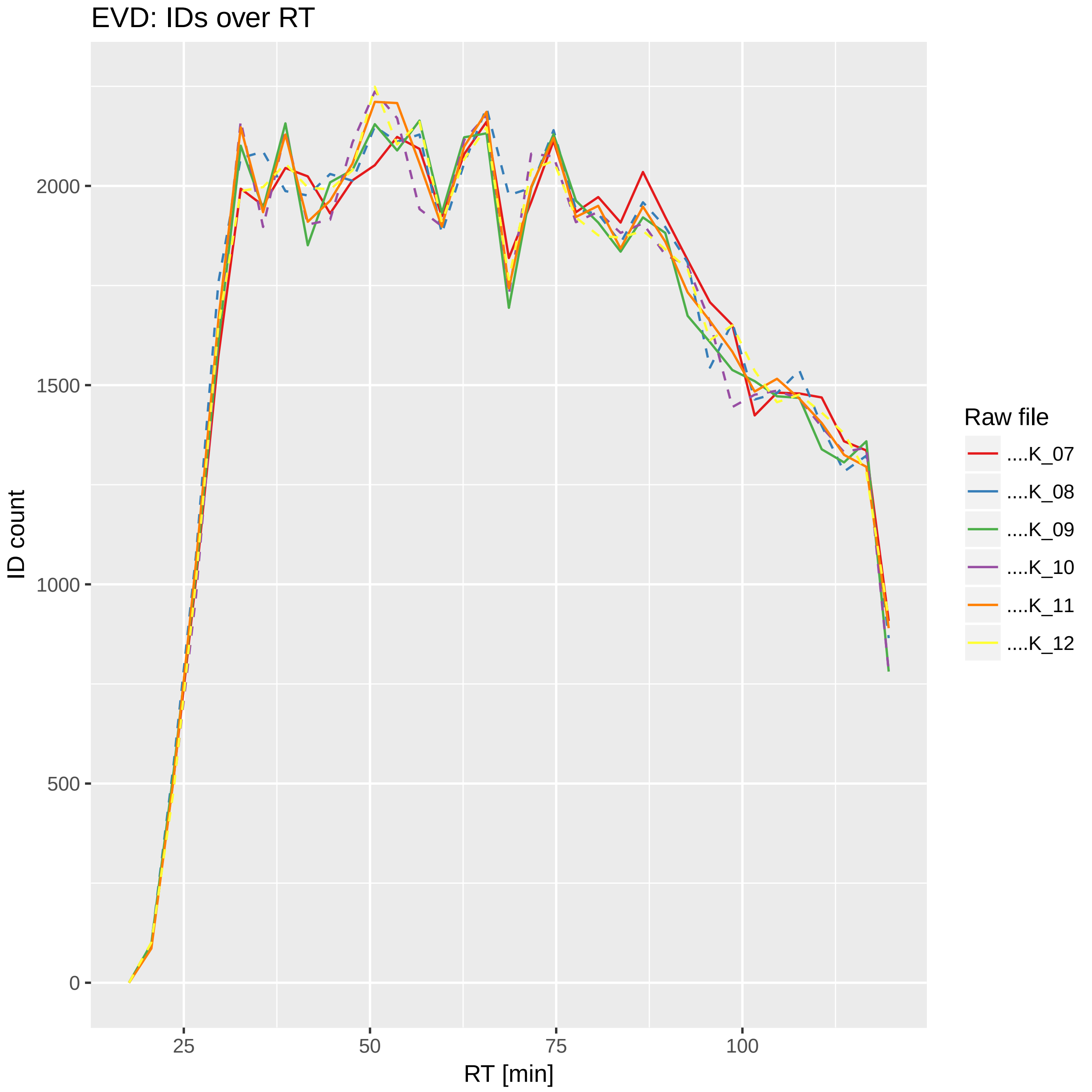
EVD: IDs over RT
ID count
0
500
1000
1500
2000
25
50
75
100
RT [min]
Raw file
....K_07
....K_08
....K_09
....K_10
....K_11
....K_12

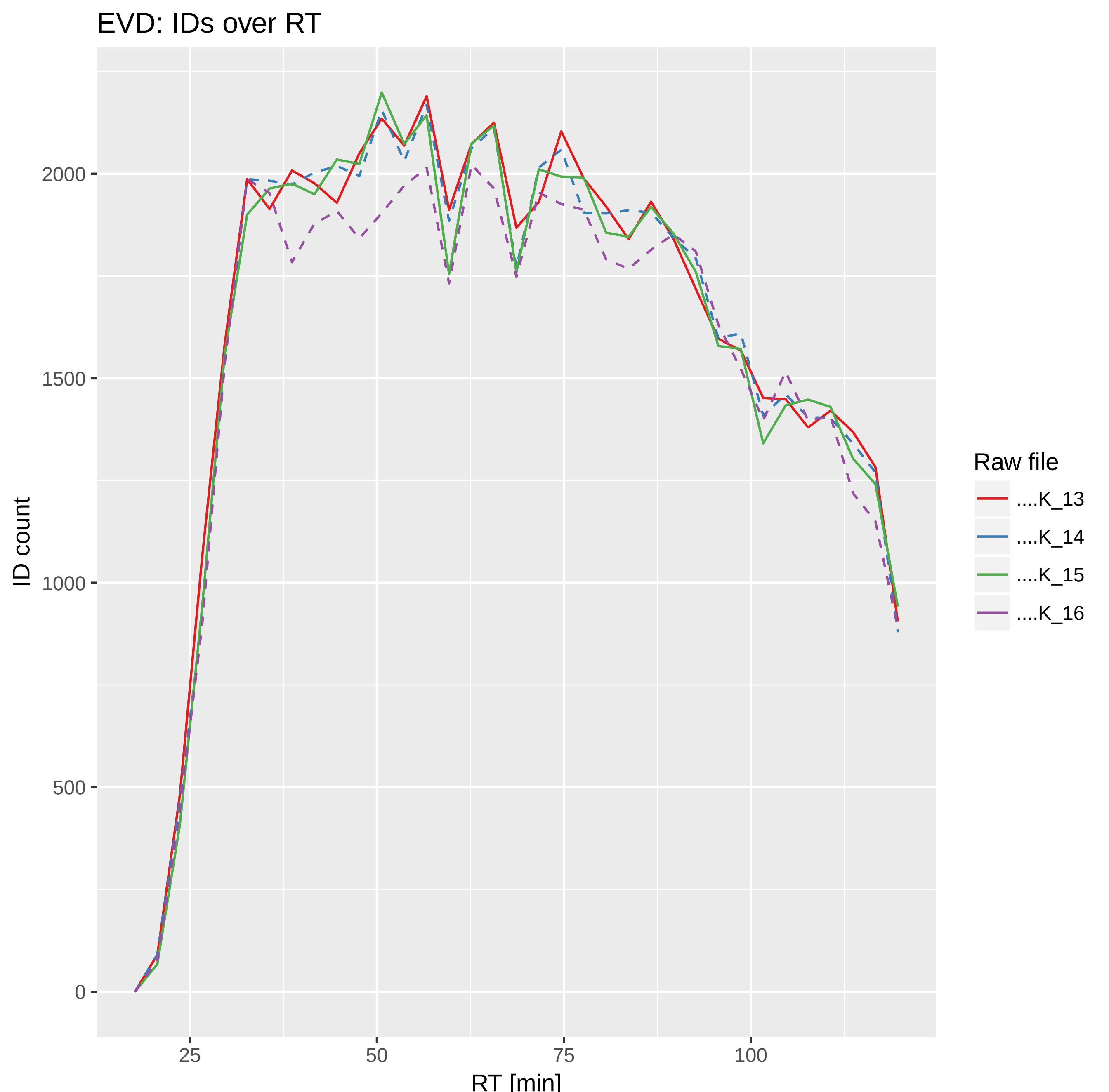
EVD: IDs over RT
ID count
2000
1500
1000
500
0
25
50
75
100
RT [min]
Raw file
....K_13
....K_14
....K_15
....K_16

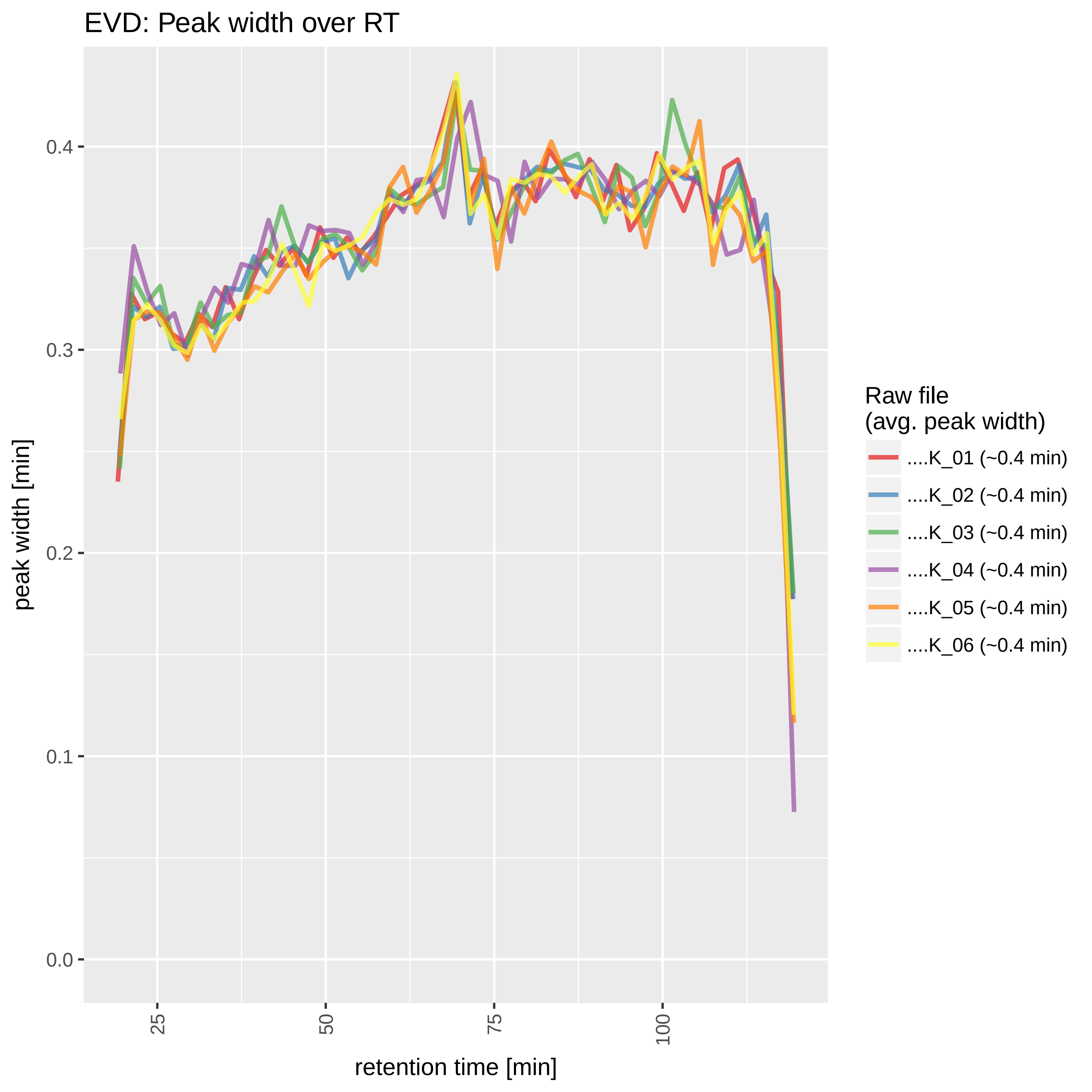

EVD: Peak width over RT
peak width [min]
0.0
0.1
0.2
0.3
0.4
retention time [min]
25
50
75
100
Raw file
(avg. peak width)
....K_01 (~0.4 min)
....K_02 (~0.4 min)
....K_03 (~0.4 min)
....K_04 (~0.4 min)
....K_05 (~0.4 min)
....K_06 (~0.4 min)

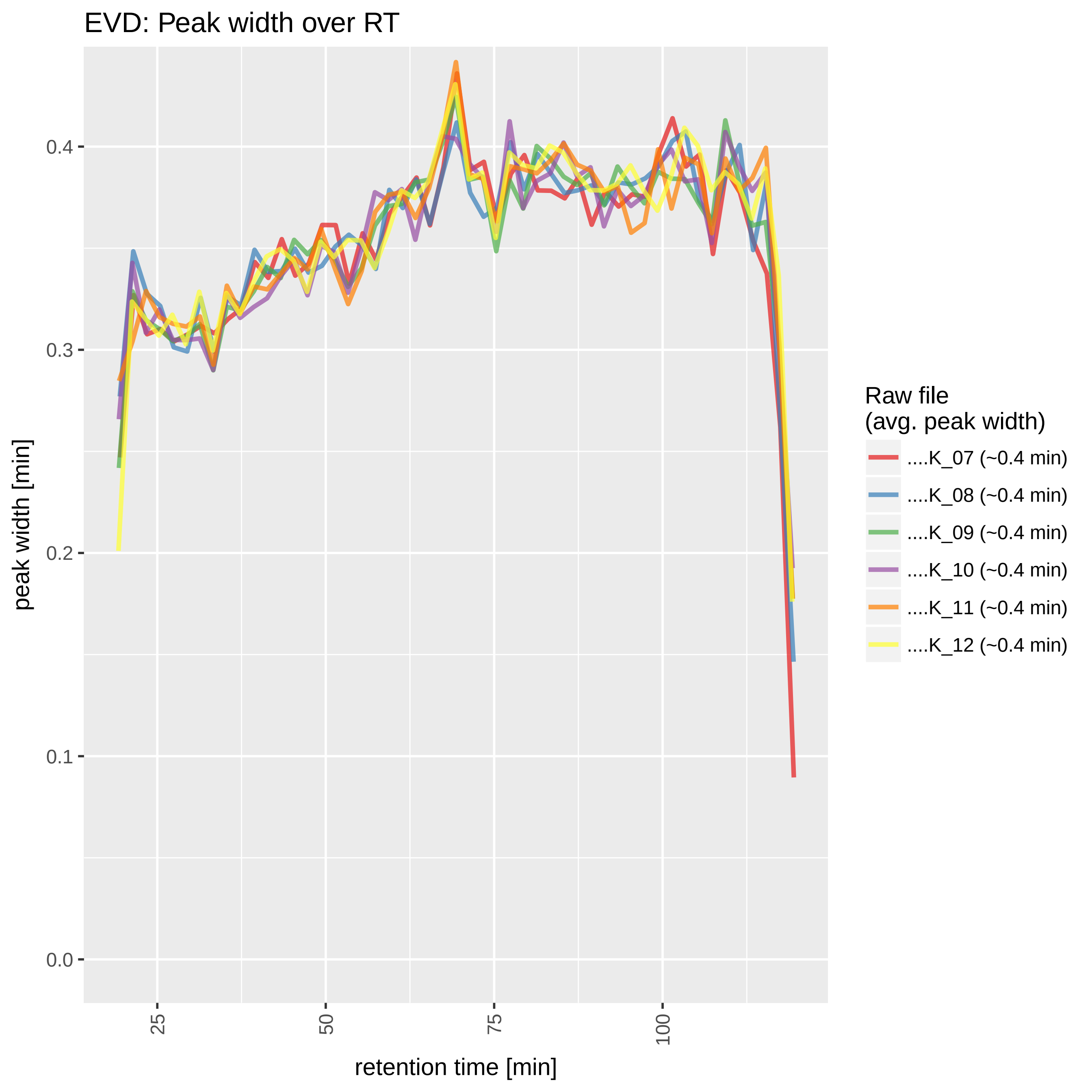
EVD: Peak width over RT
peak width [min]
0.0
0.1
0.2
0.3
0.4
25
50
75
100
retention time [min]
Raw file
(avg. peak width)
....K_07 (~0.4 min)
....K_08 (~0.4 min)
....K_09 (~0.4 min)
....K_10 (~0.4 min)
....K_11 (~0.4 min)
....K_12 (~0.4 min)

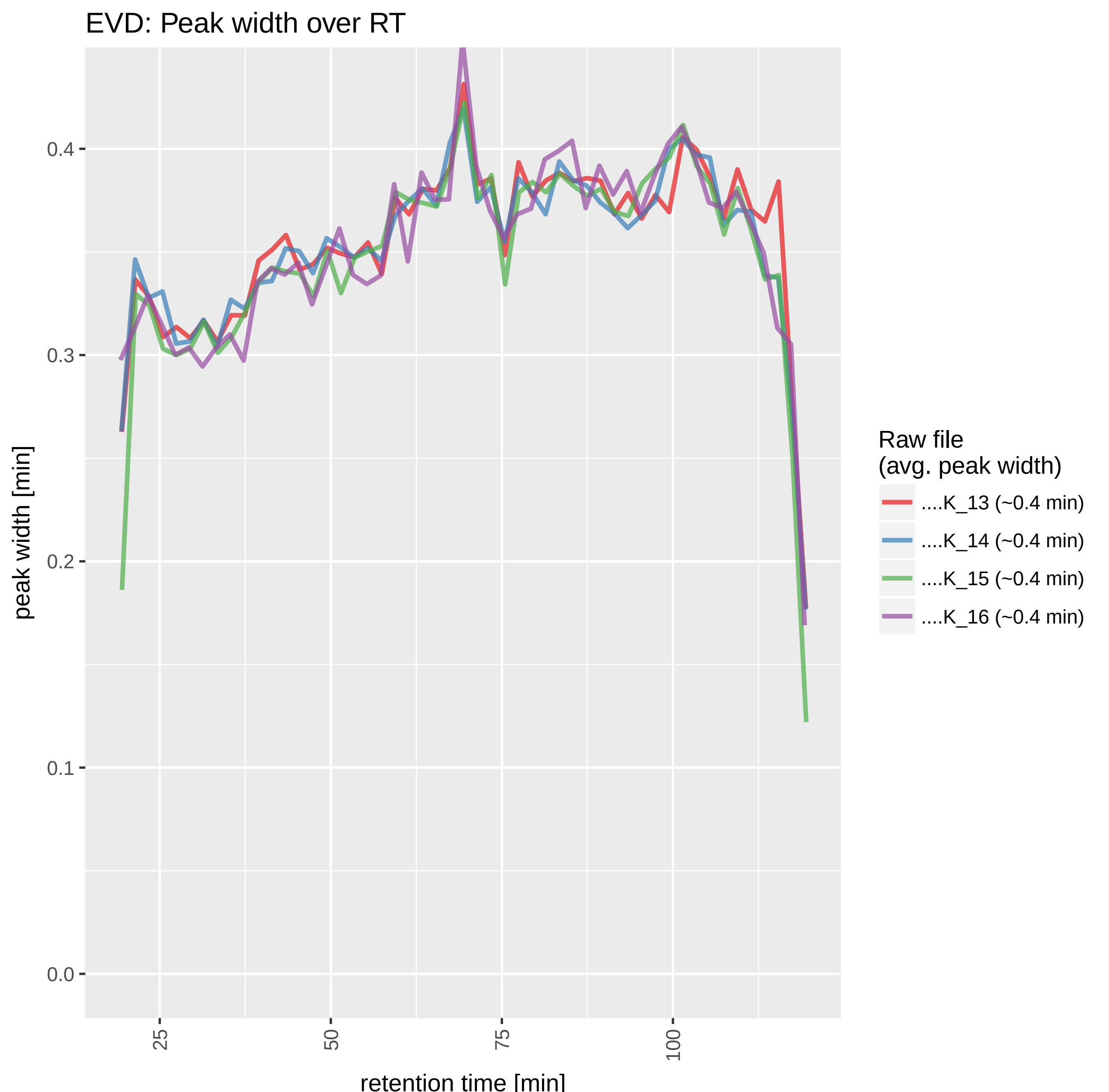
EVD: Peak width over RT
peak width [min]
0.0
0.1
0.2
0.3
0.4
25
50
75
100
retention time [min]
Raw file
(avg. peak width)
....K_13 (~0.4 min)
....K_14 (~0.4 min)
....K_15 (~0.4 min)
....K_16 (~0.4 min)

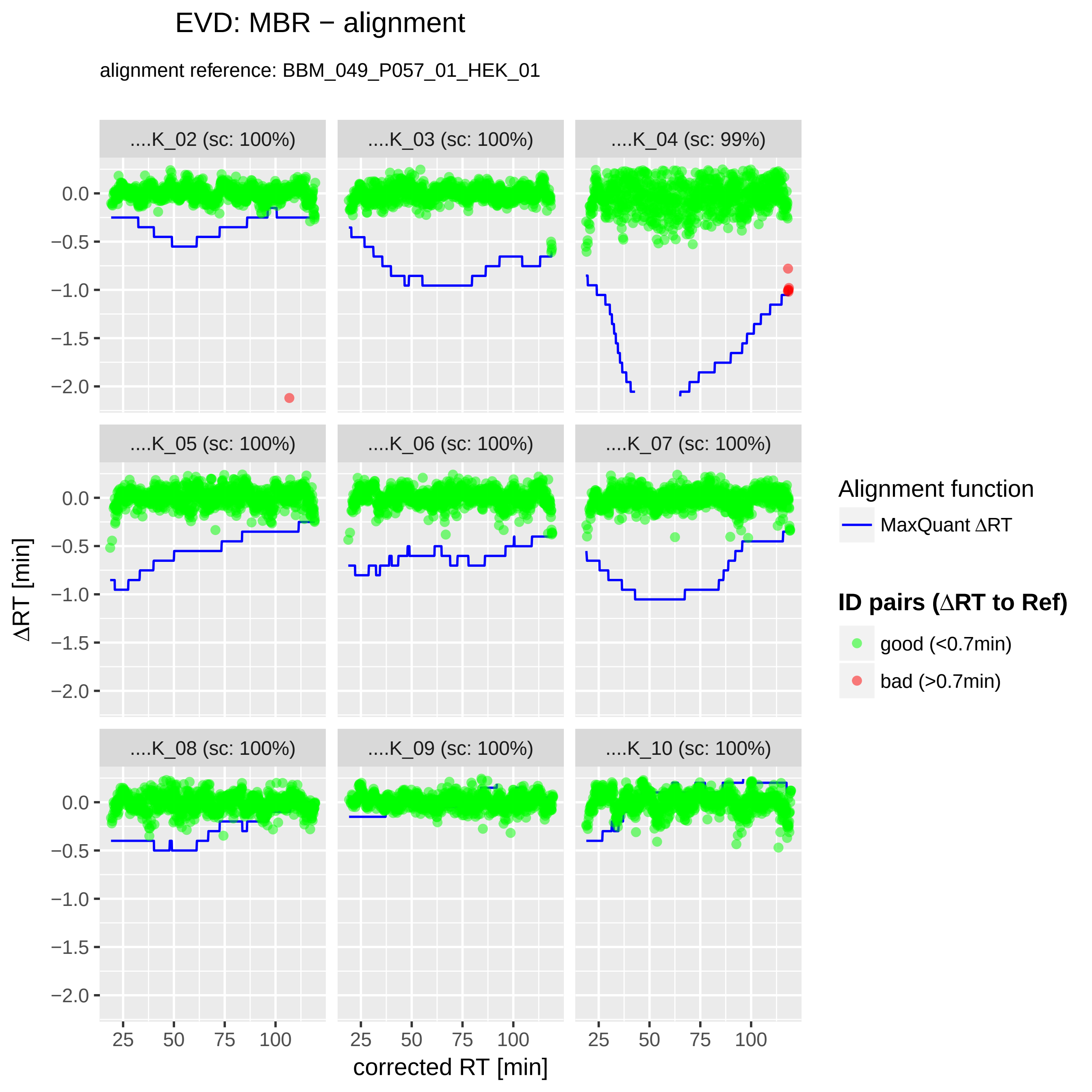
EVD: MBR − alignment
alignment reference: BBM_049_P057_01_HEK_01
....K_02 (sc: 100%)
....K_03 (sc: 100%)
....K_04 (sc: 99%)
....K_05 (sc: 100%)
....K_06 (sc: 100%)
....K_07 (sc: 100%)
....K_08 (sc: 100%)
....K_09 (sc: 100%)
....K_10 (sc: 100%)
ΔRT [min]
0.0
−0.5
−1.0
−1.5
−2.0
25
50
75
100
corrected RT [min]
Alignment function
MaxQuant ΔRT
ID pairs (ΔRT to Ref)
good (<0.7min)
bad (>0.7min)

EVD: MBR − alignment

alignment reference: BBM_049_P057_01_HEK_01

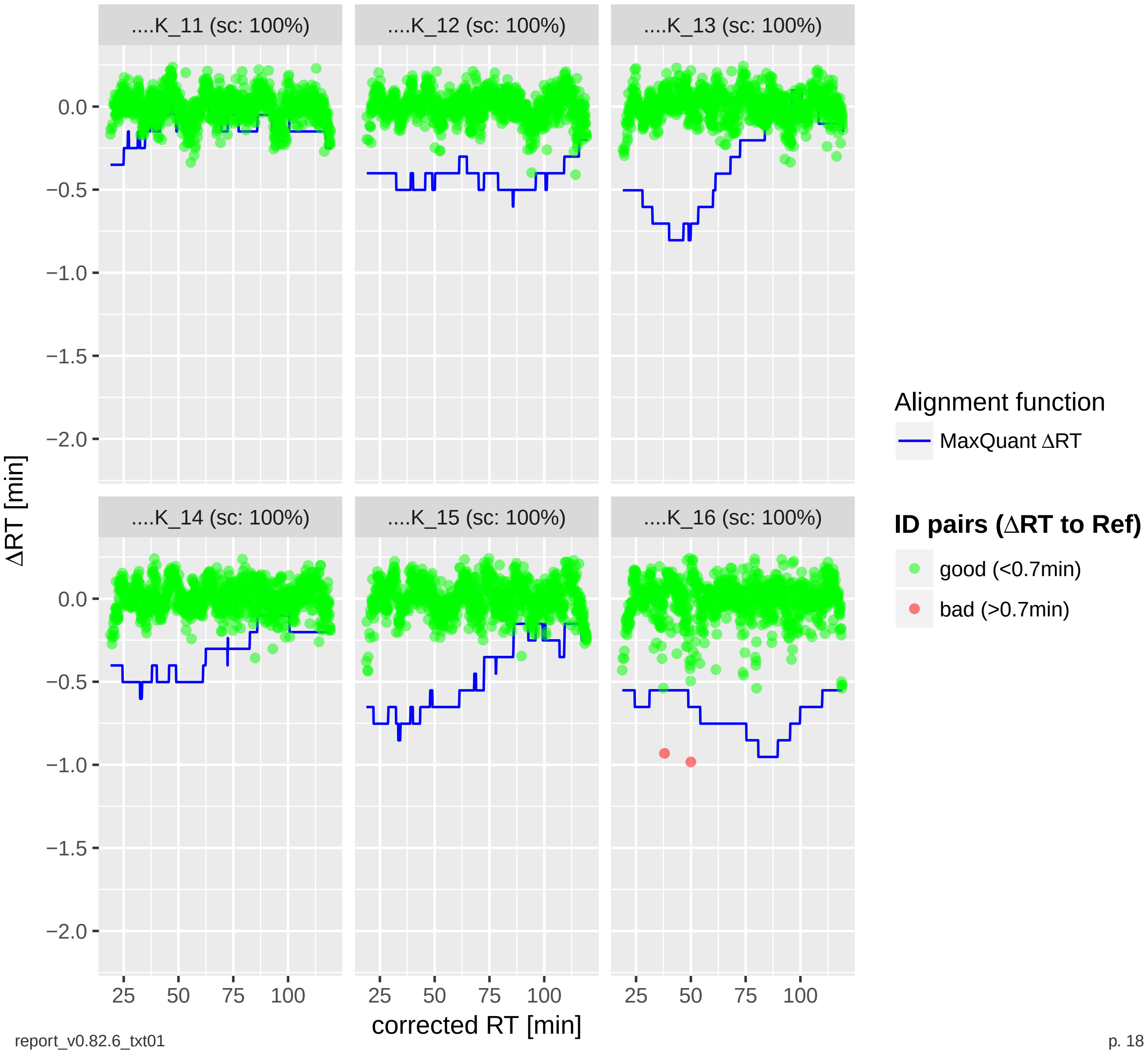

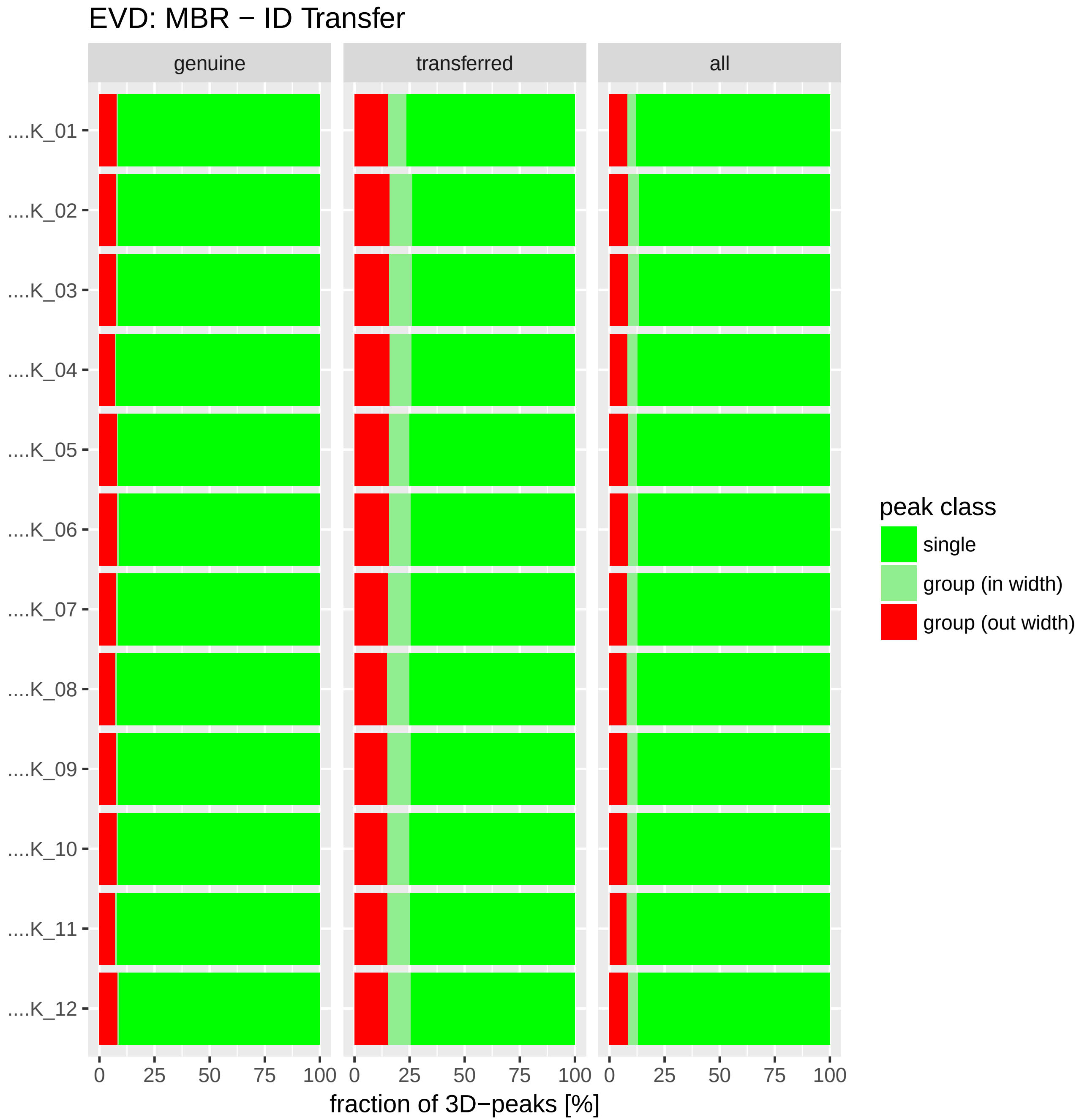

EVD: MBR − ID Transfer
genuine
transferred
all
....K_01
....K_02
....K_03
....K_04
....K_05
....K_06
....K_07
....K_08
....K_09
....K_10
....K_11
....K_12
0
25
50
75
100
fraction of 3D−peaks [%]
peak class
single
group (in width)
group (out width)

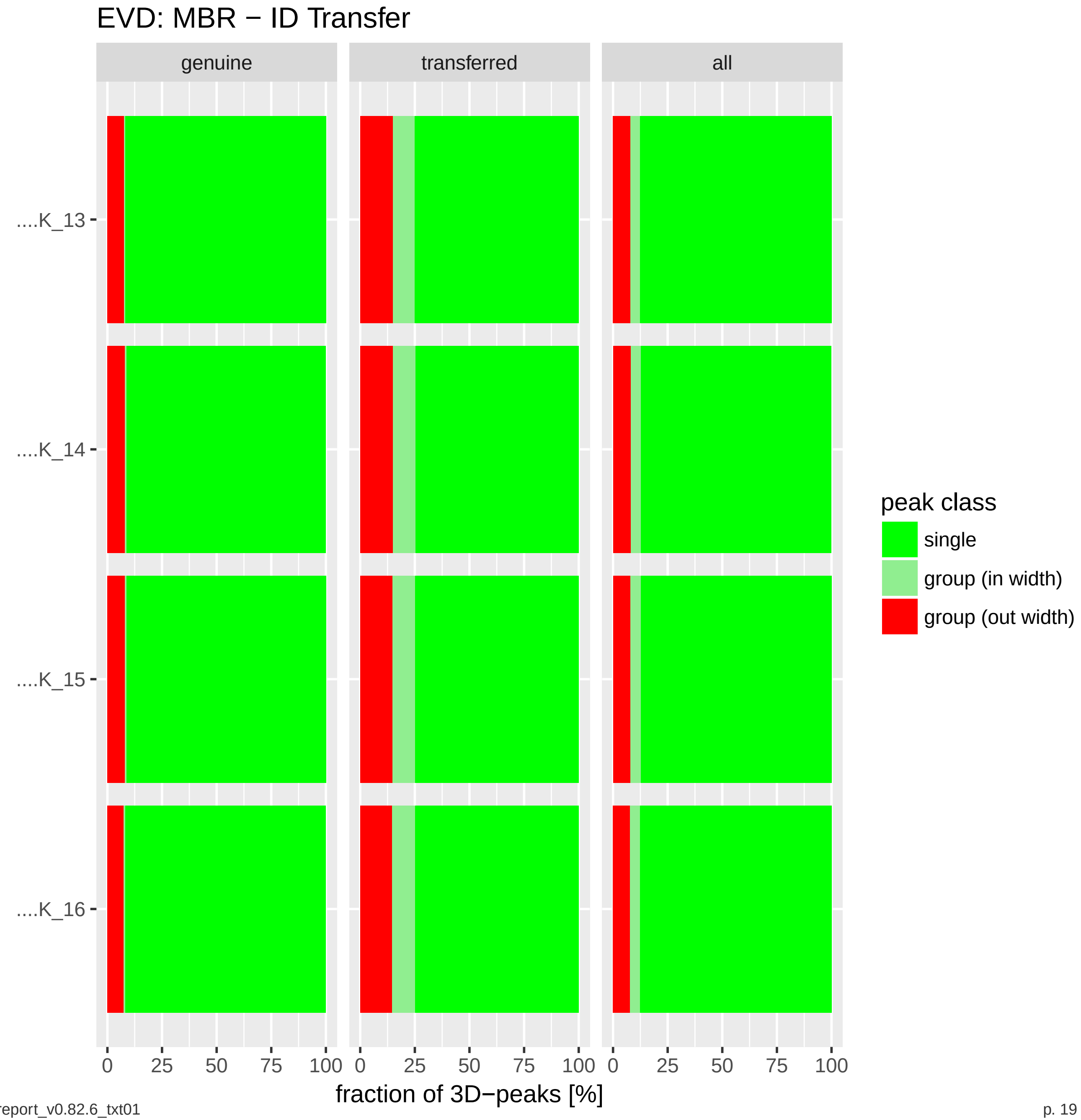
EVD: MBR − ID Transfer
genuine
transferred
all
....K_13
....K_14
....K_15
....K_16
0
25
50
75
100
fraction of 3D−peaks [%]
peak class
single
group (in width)
group (out width)

[experimental] EVD: Clustering Tree of Raw files

by Correlation of Corrected Retention Times

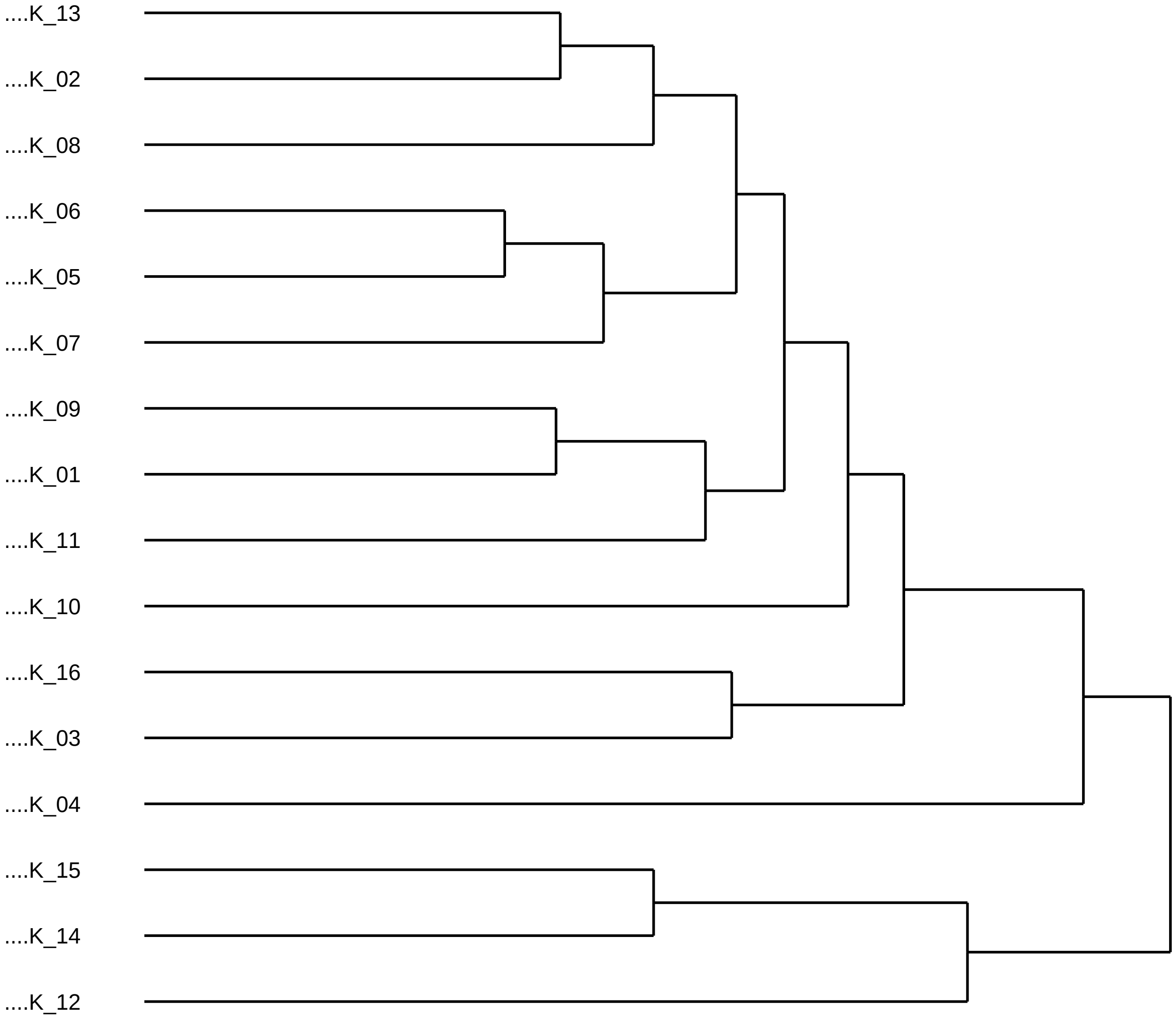

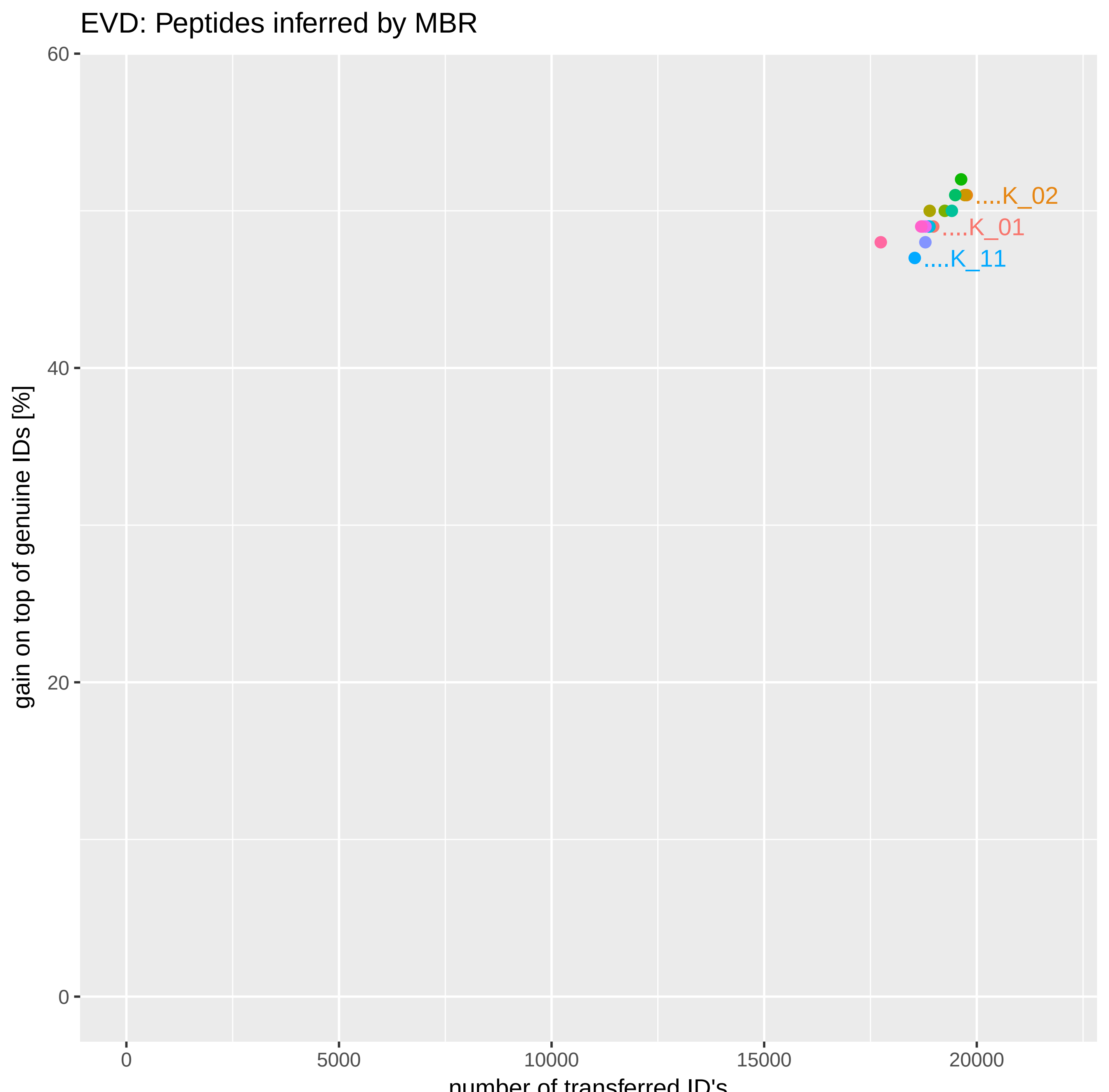
EVD: Peptides inferred by MBR
gain on top of genuine IDs [%]
number of transferred ID's
0
20
40
60
0
5000
10000
15000
20000
....K_02
....K_01
....K_11

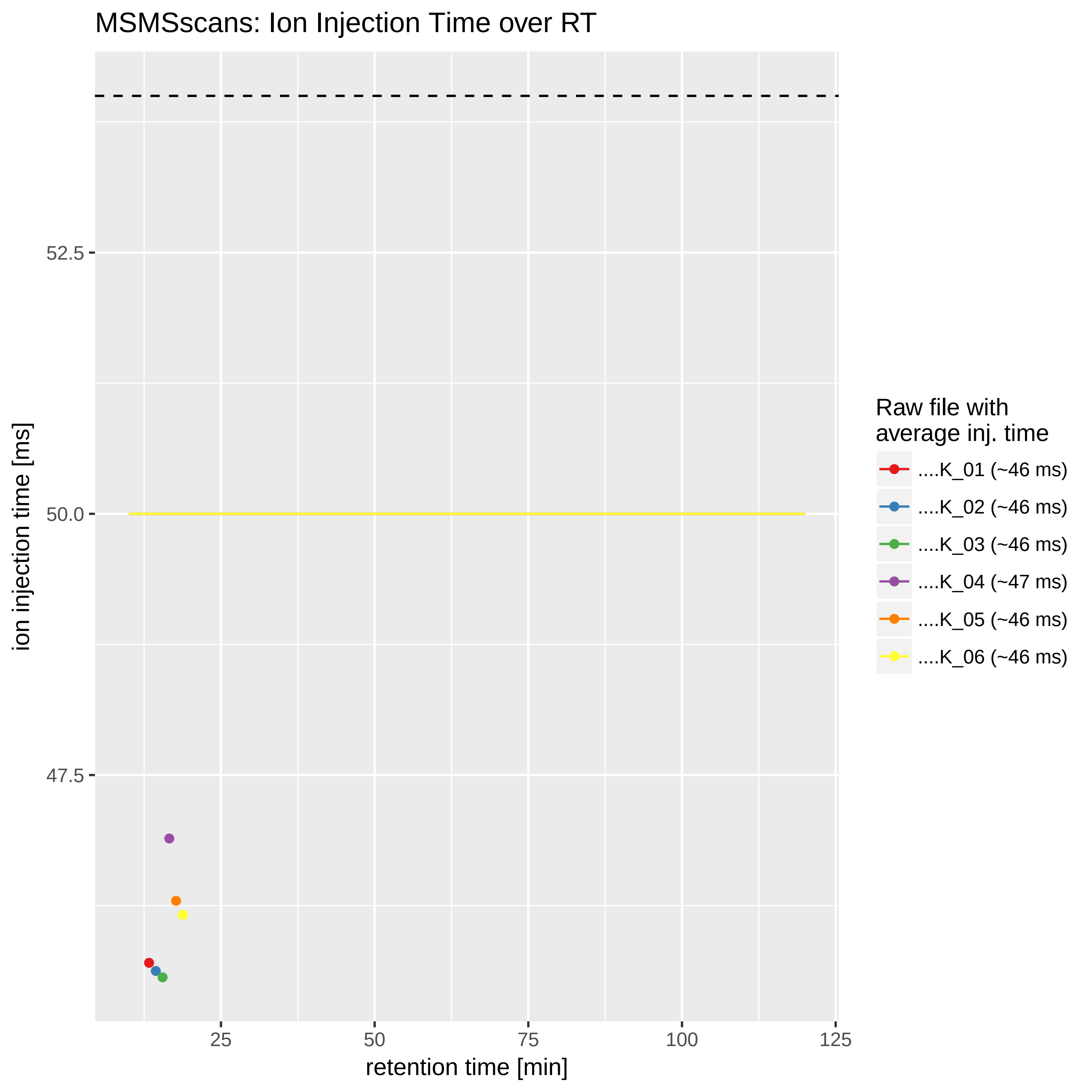

MSMSscans: Ion Injection Time over RT
52.5
50.0
47.5
ion injection time [ms]
25
50
75
100
125
retention time [min]
Raw file with
average inj. time
....K_01 (~46 ms)
....K_02 (~46 ms)
....K_03 (~46 ms)
....K_04 (~47 ms)
....K_05 (~46 ms)
....K_06 (~46 ms)

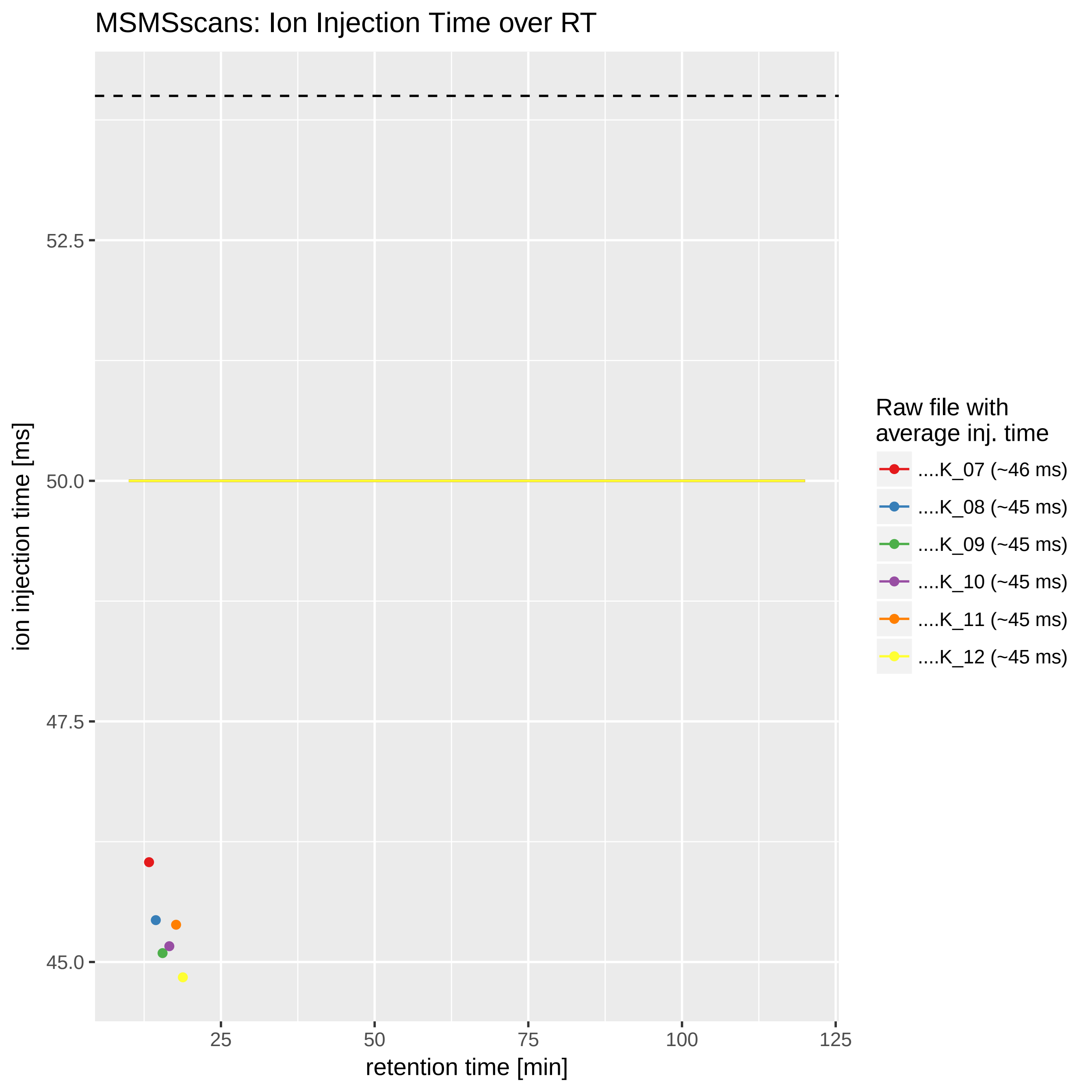
MSMSscans: Ion Injection Time over RT
ion injection time [ms]
52.5
50.0
47.5
45.0
25
50
75
100
125
retention time [min]
Raw file with
average inj. time
....K_07 (~46 ms)
....K_08 (~45 ms)
....K_09 (~45 ms)
....K_10 (~45 ms)
....K_11 (~45 ms)
....K_12 (~45 ms)

MSMSscans: Ion Injection Time over RT

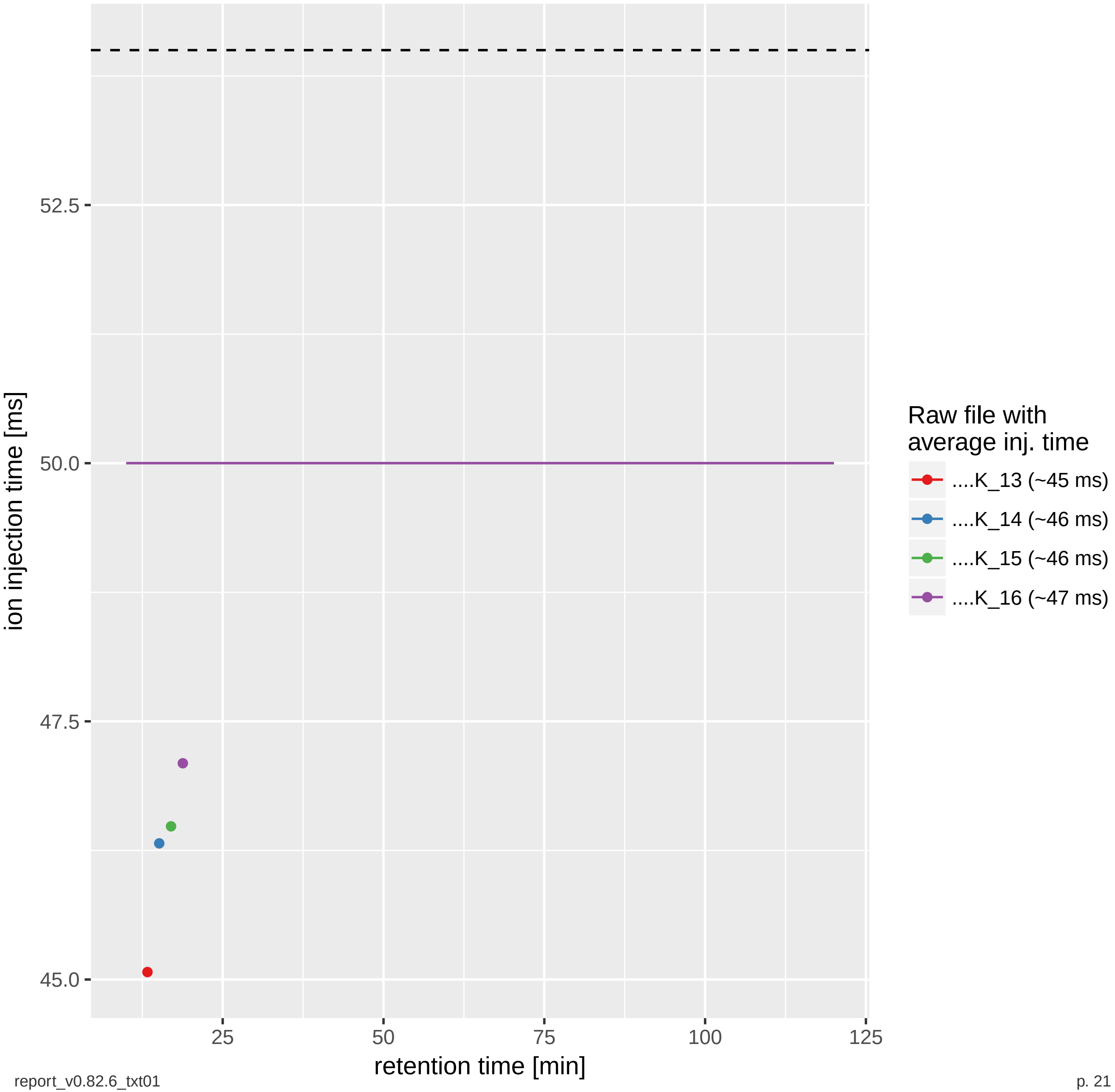

[experimental] MSMSscans: MS/MS intensity

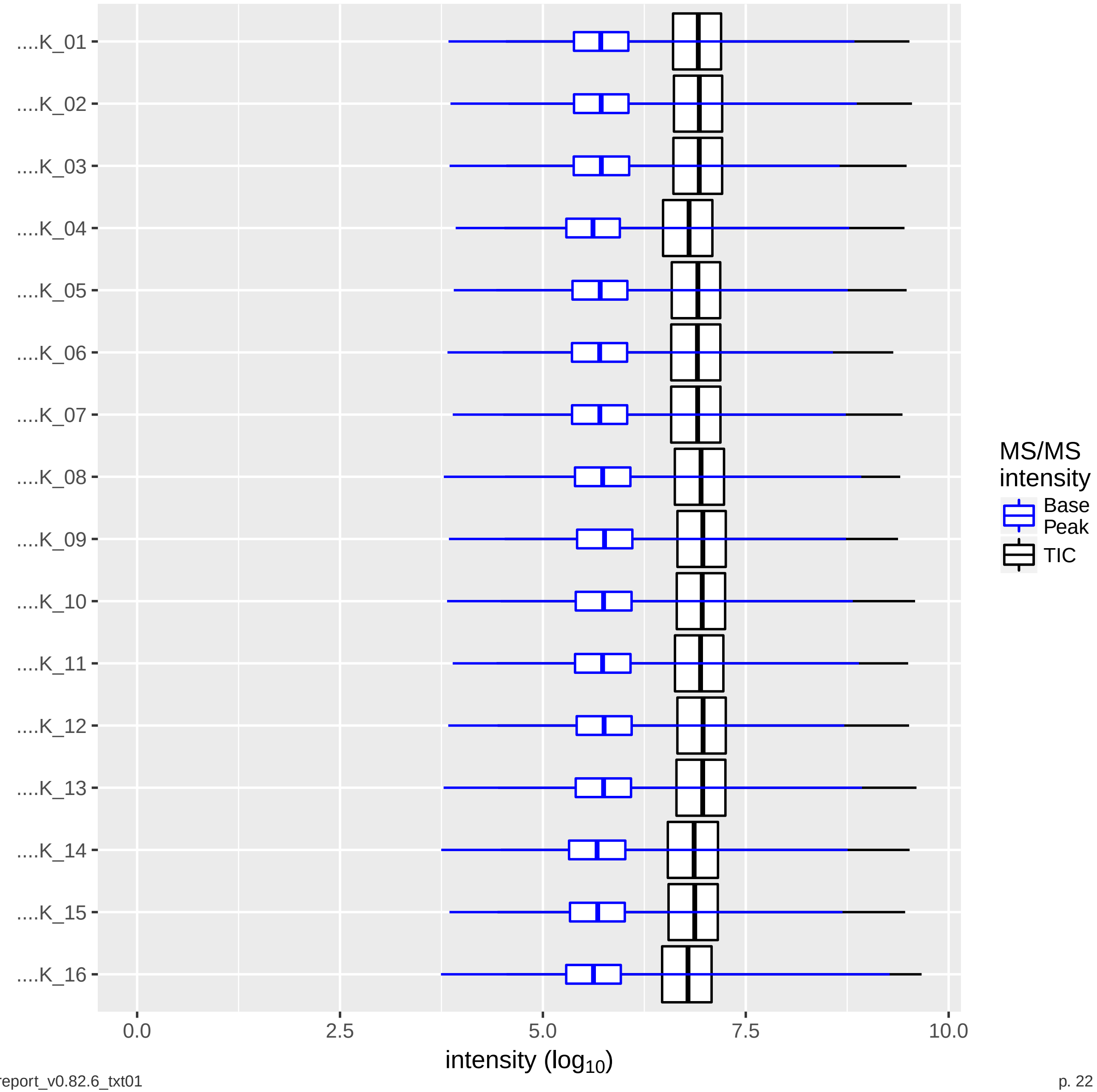

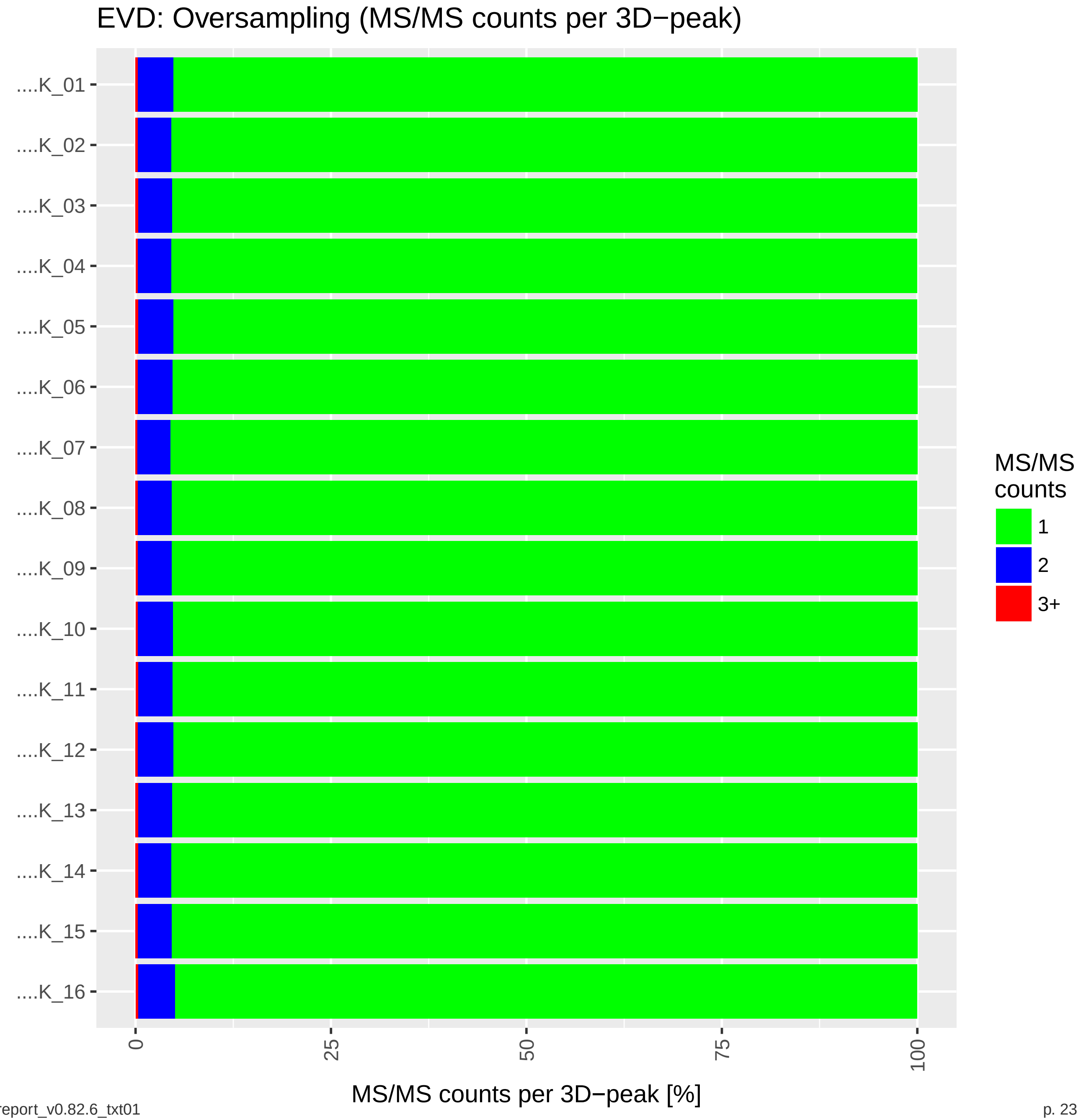
EVD: Oversampling (MS/MS counts per 3D−peak)
....K_01
....K_02
....K_03
....K_04
....K_05
....K_06
....K_07
....K_08
....K_09
....K_10
....K_11
....K_12
....K_13
....K_14
....K_15
....K_16
0
25
50
75
100
MS/MS counts per 3D−peak [%]
MS/MS counts
1
2
3+

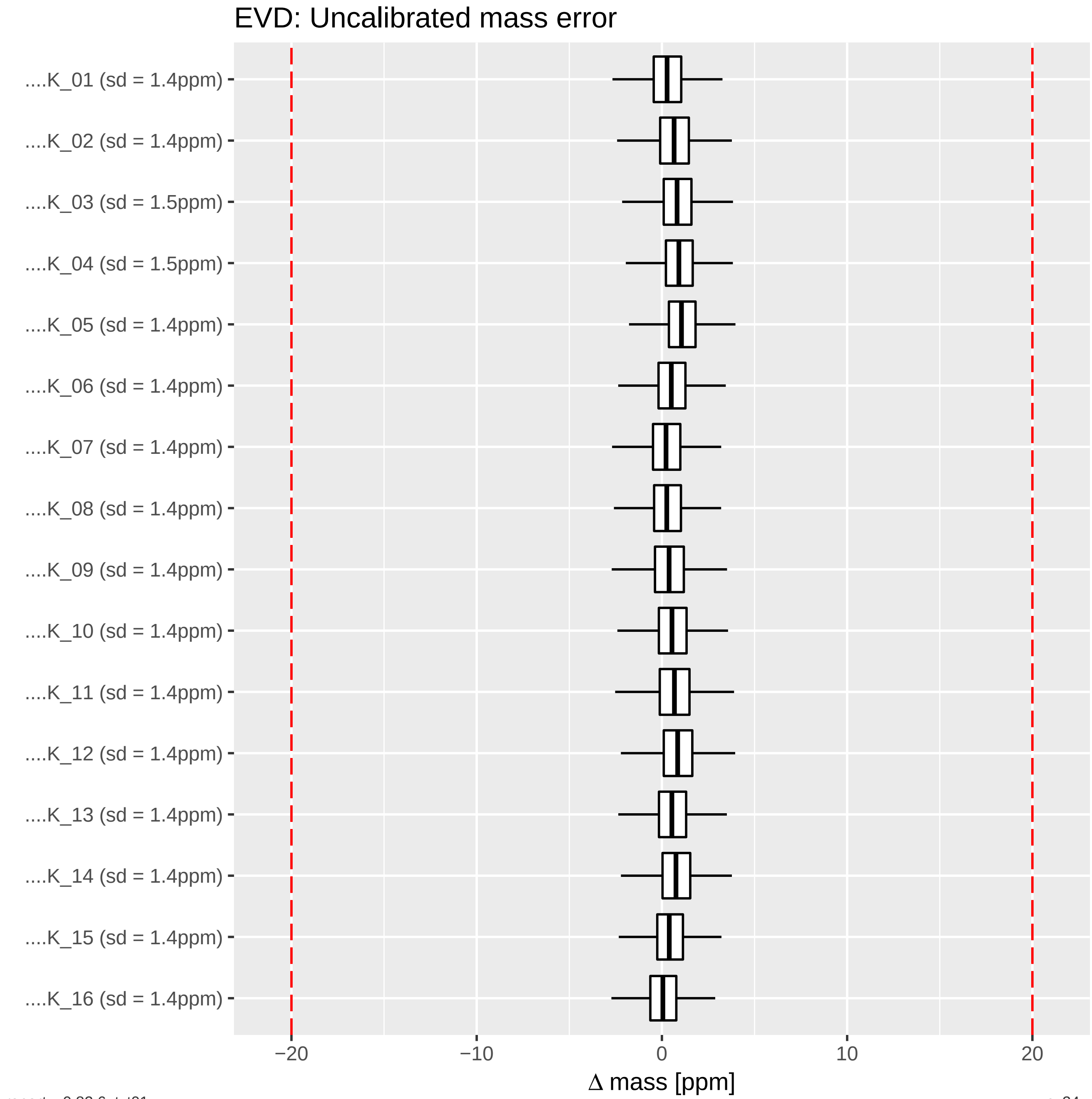
EVD: Uncalibrated mass error
....K_01 (sd = 1.4ppm)
....K_02 (sd = 1.4ppm)
....K_03 (sd = 1.5ppm)
....K_04 (sd = 1.5ppm)
....K_05 (sd = 1.4ppm)
....K_06 (sd = 1.4ppm)
....K_07 (sd = 1.4ppm)
....K_08 (sd = 1.4ppm)
....K_09 (sd = 1.4ppm)
....K_10 (sd = 1.4ppm)
....K_11 (sd = 1.4ppm)
....K_12 (sd = 1.4ppm)
....K_13 (sd = 1.4ppm)
....K_14 (sd = 1.4ppm)
....K_15 (sd = 1.4ppm)
....K_16 (sd = 1.4ppm)
−20
−10
0
10
20
Δ mass [ppm]

EVD: Calibrated mass error

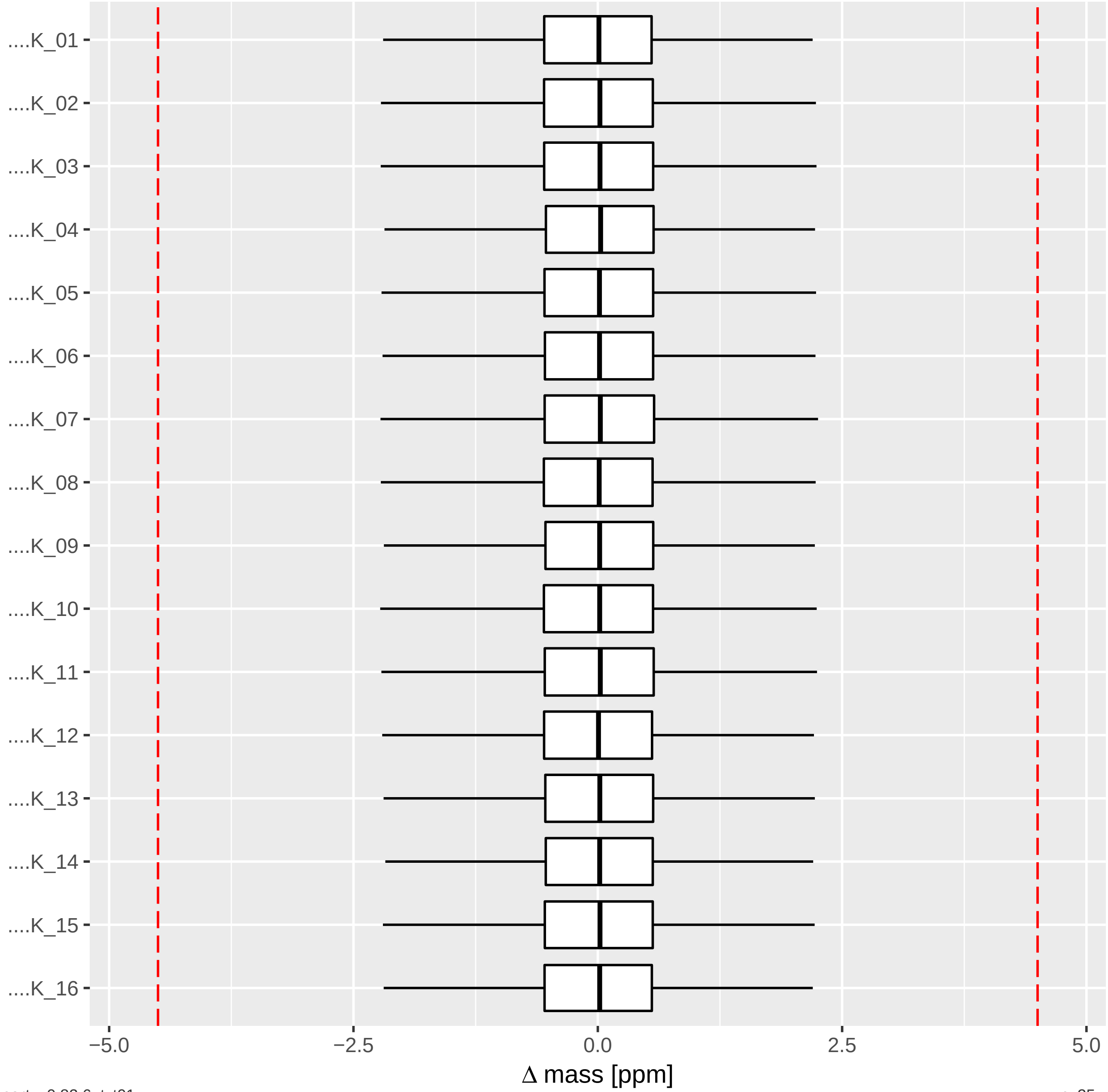

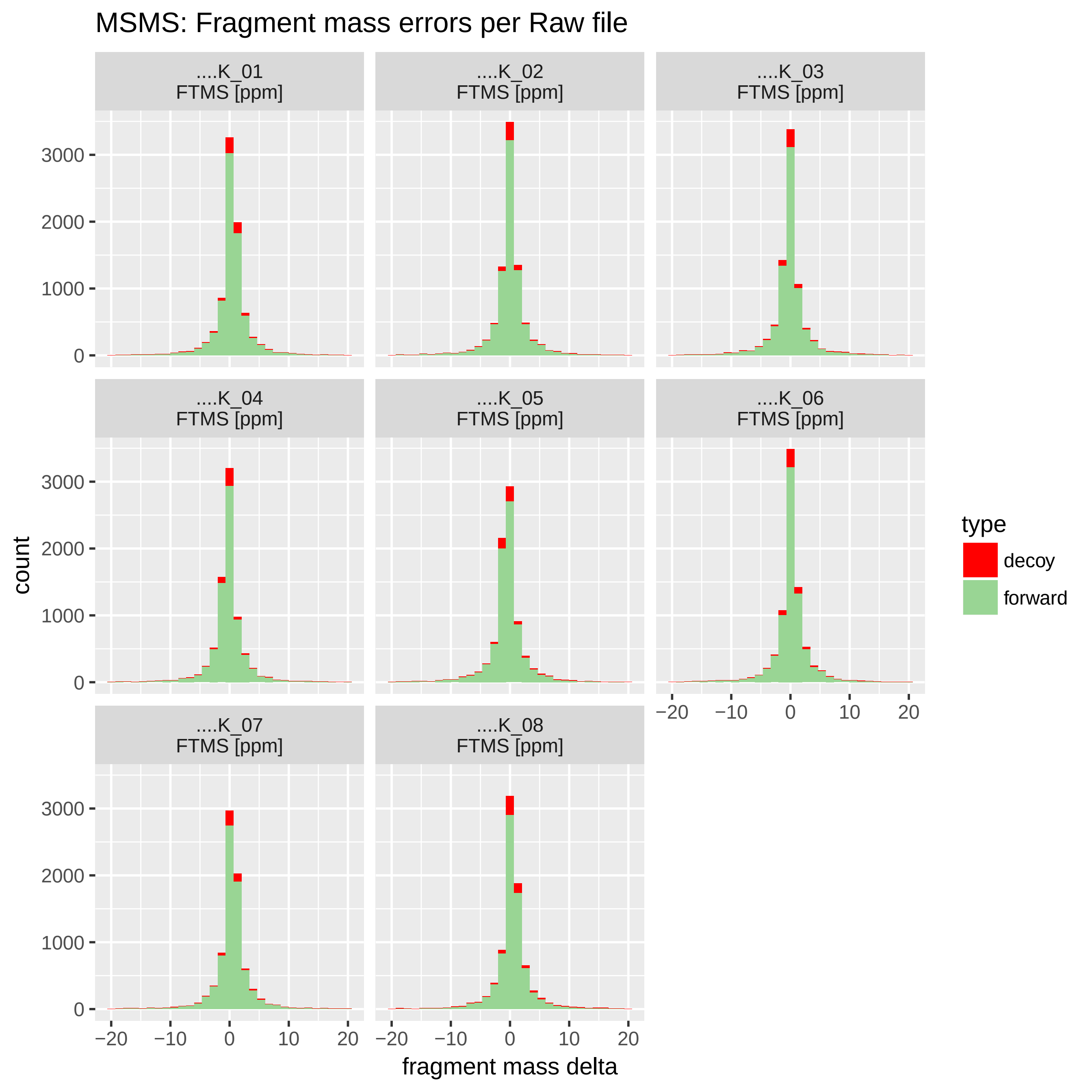
MSMS: Fragment mass errors per Raw file
....K_01
FTMS [ppm]
....K_02
FTMS [ppm]
....K_03
FTMS [ppm]
....K_04
FTMS [ppm]
....K_05
FTMS [ppm]
....K_06
FTMS [ppm]
....K_07
FTMS [ppm]
....K_08
FTMS [ppm]
count
0
1000
2000
3000
−20
−10
0
10
20
fragment mass delta
type
decoy
forward

MSMS: Fragment mass errors per Raw file

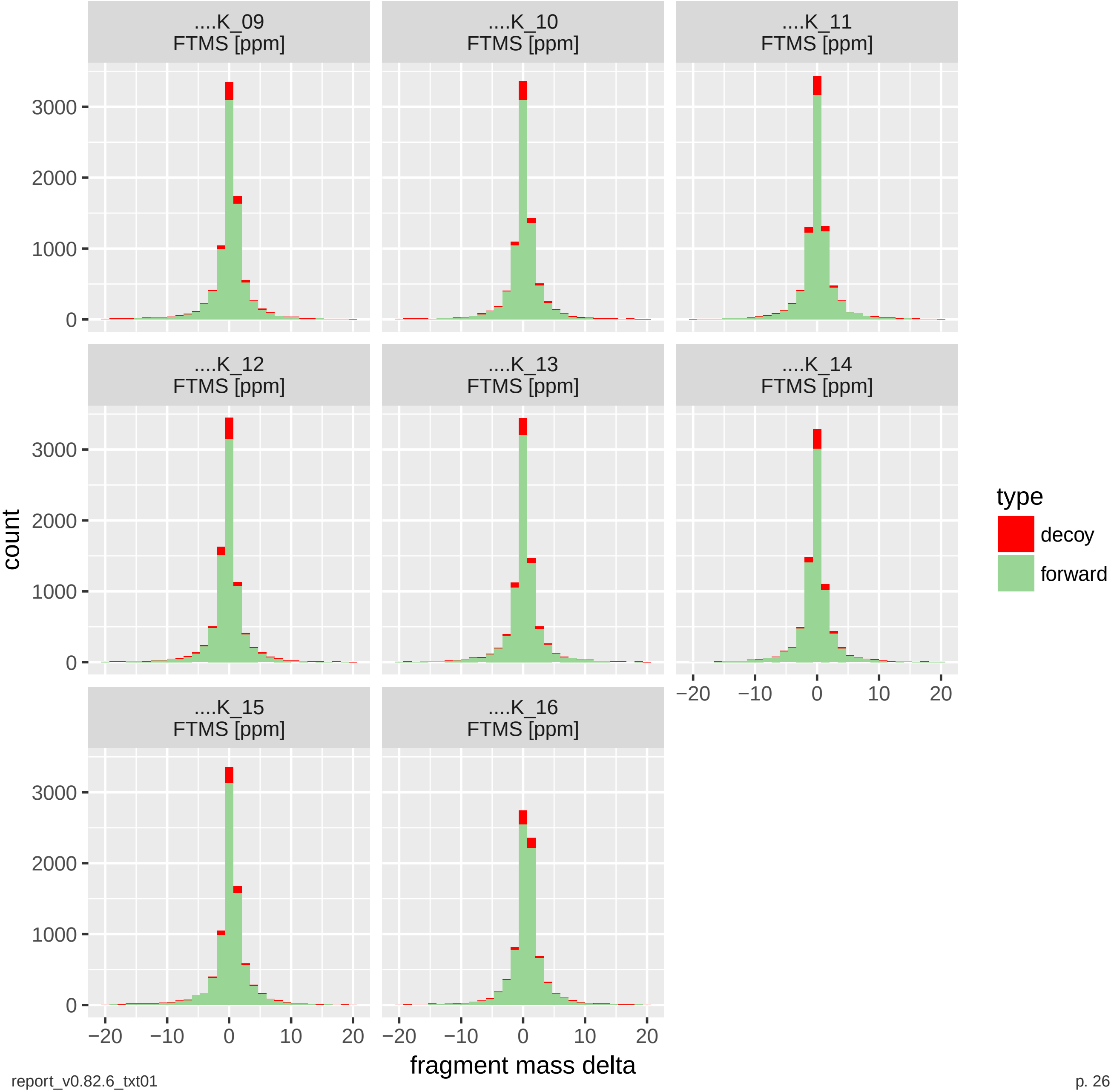

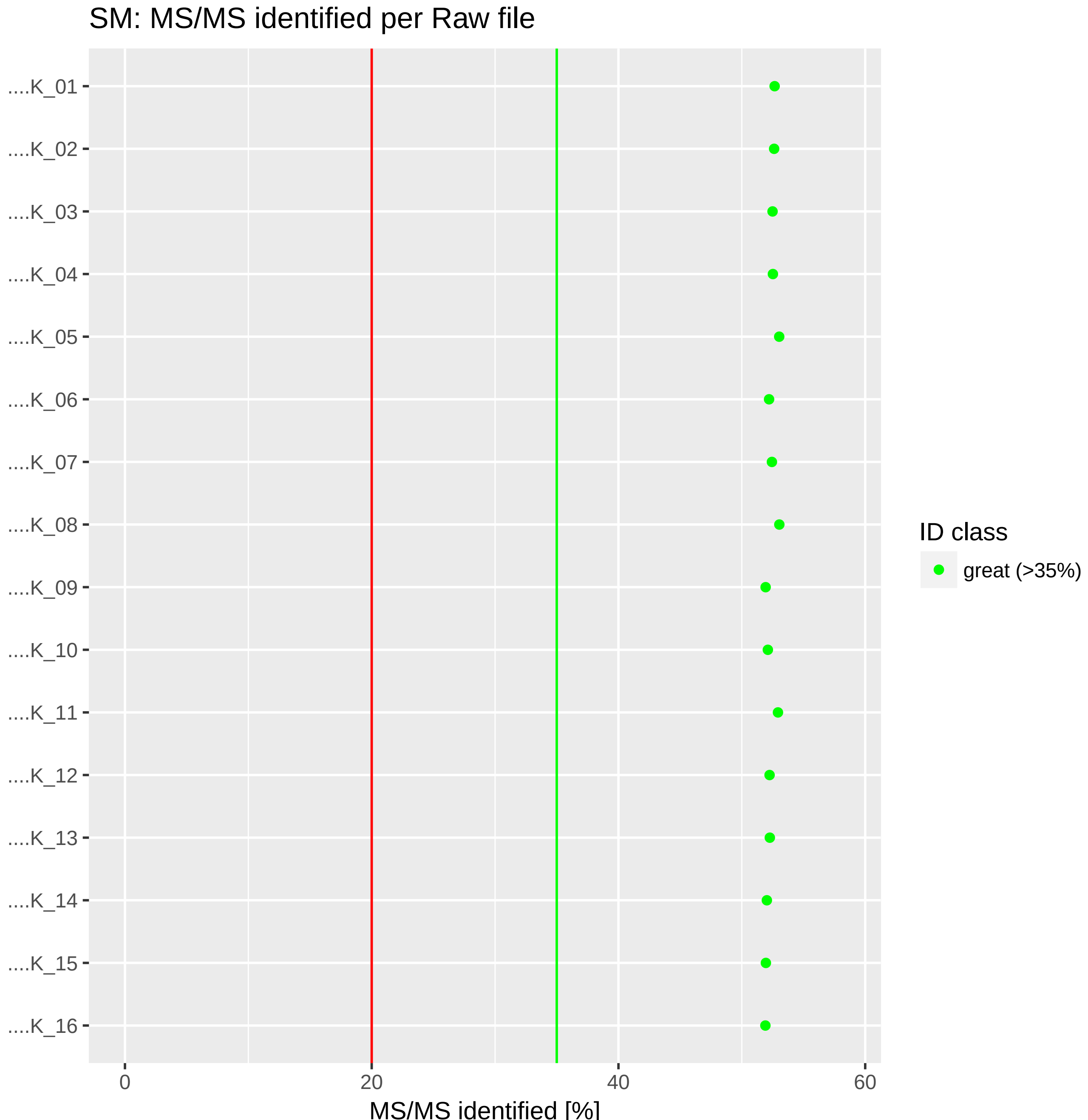

SM: MS/MS identified per Raw file
....K_01
....K_02
....K_03
....K_04
....K_05
....K_06
....K_07
....K_08
....K_09
....K_10
....K_11
....K_12
....K_13
....K_14
....K_15
....K_16
0
20
40
60
MS/MS identified [%]
ID class
great (>35%)

MSMSscans: TopN

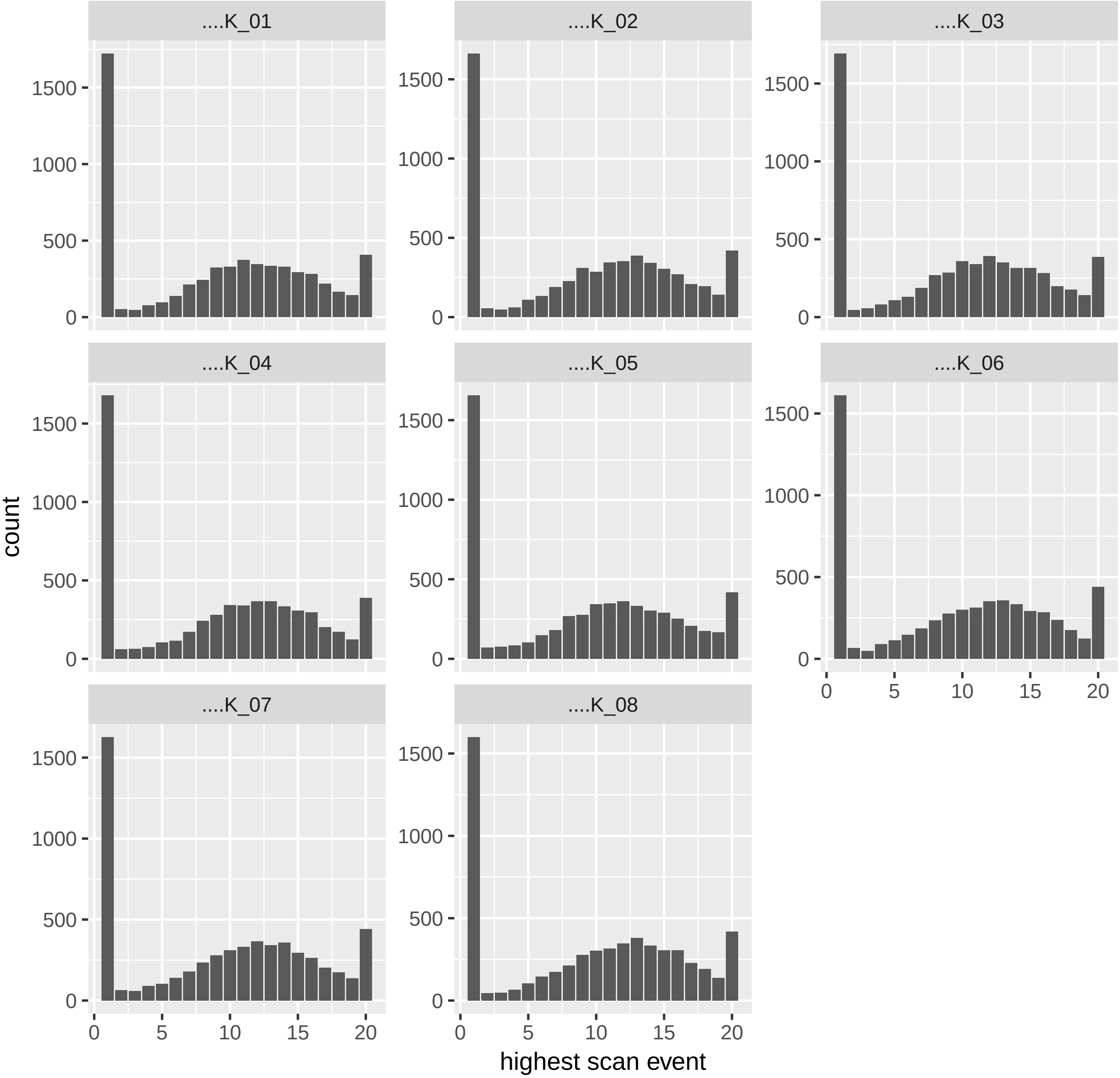

MSMSscans: TopN

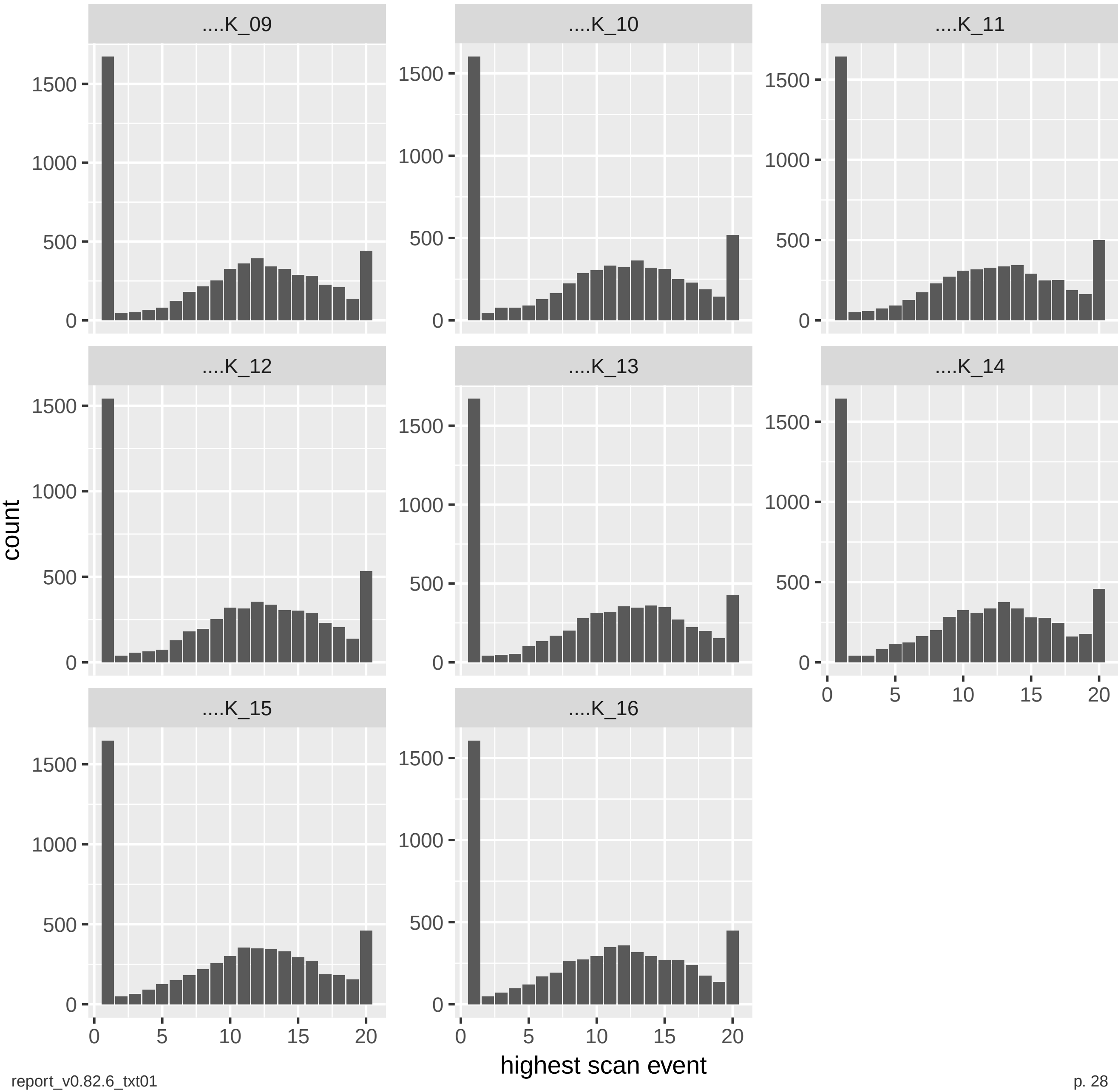

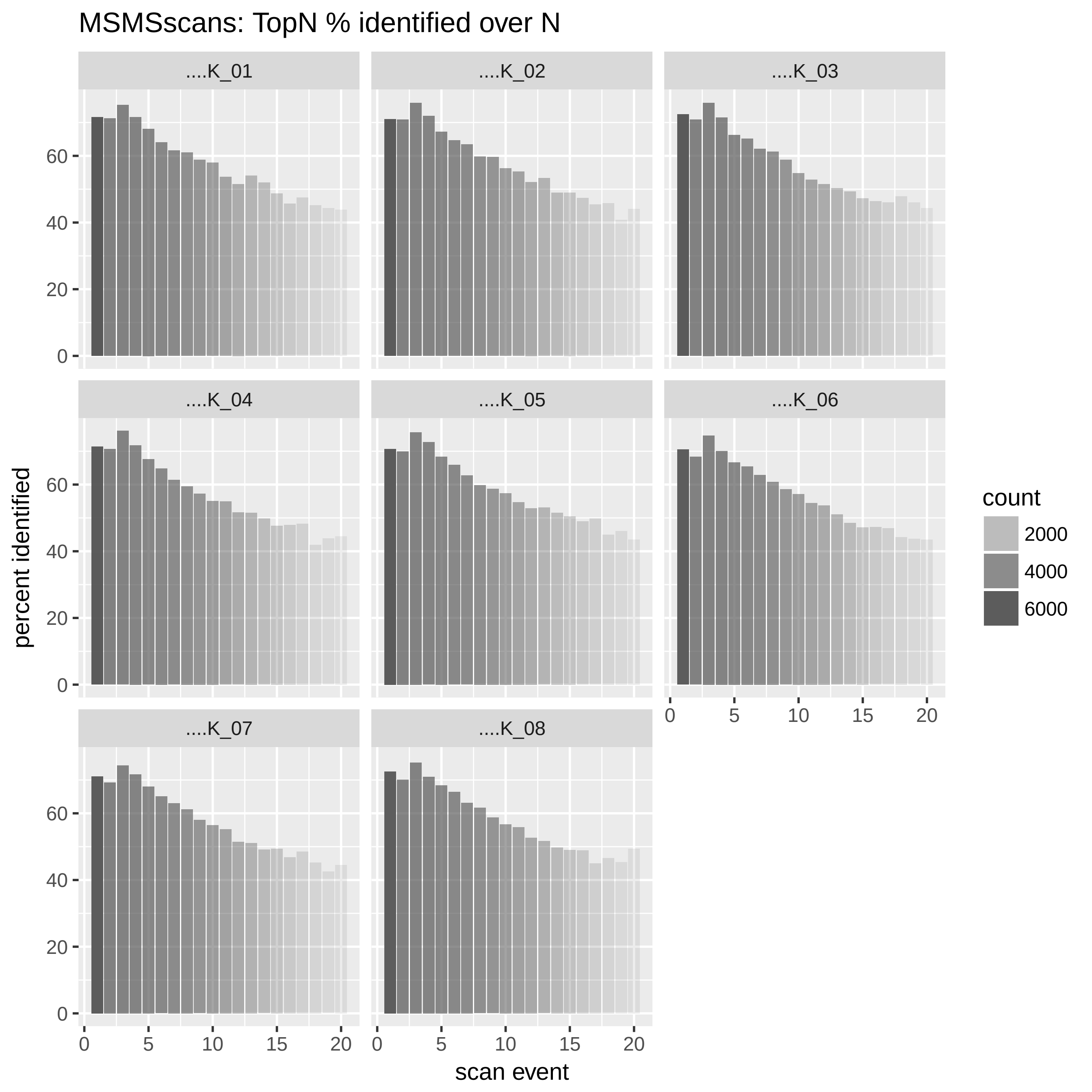
MSMSscans: TopN % identified over N
....K_01
....K_02
....K_03
....K_04
....K_05
....K_06
....K_07
....K_08
percent identified
scan event
count
2000
4000
6000
0
20
40
60
0
5
10
15
20

MSMSscans: TopN % identified over N

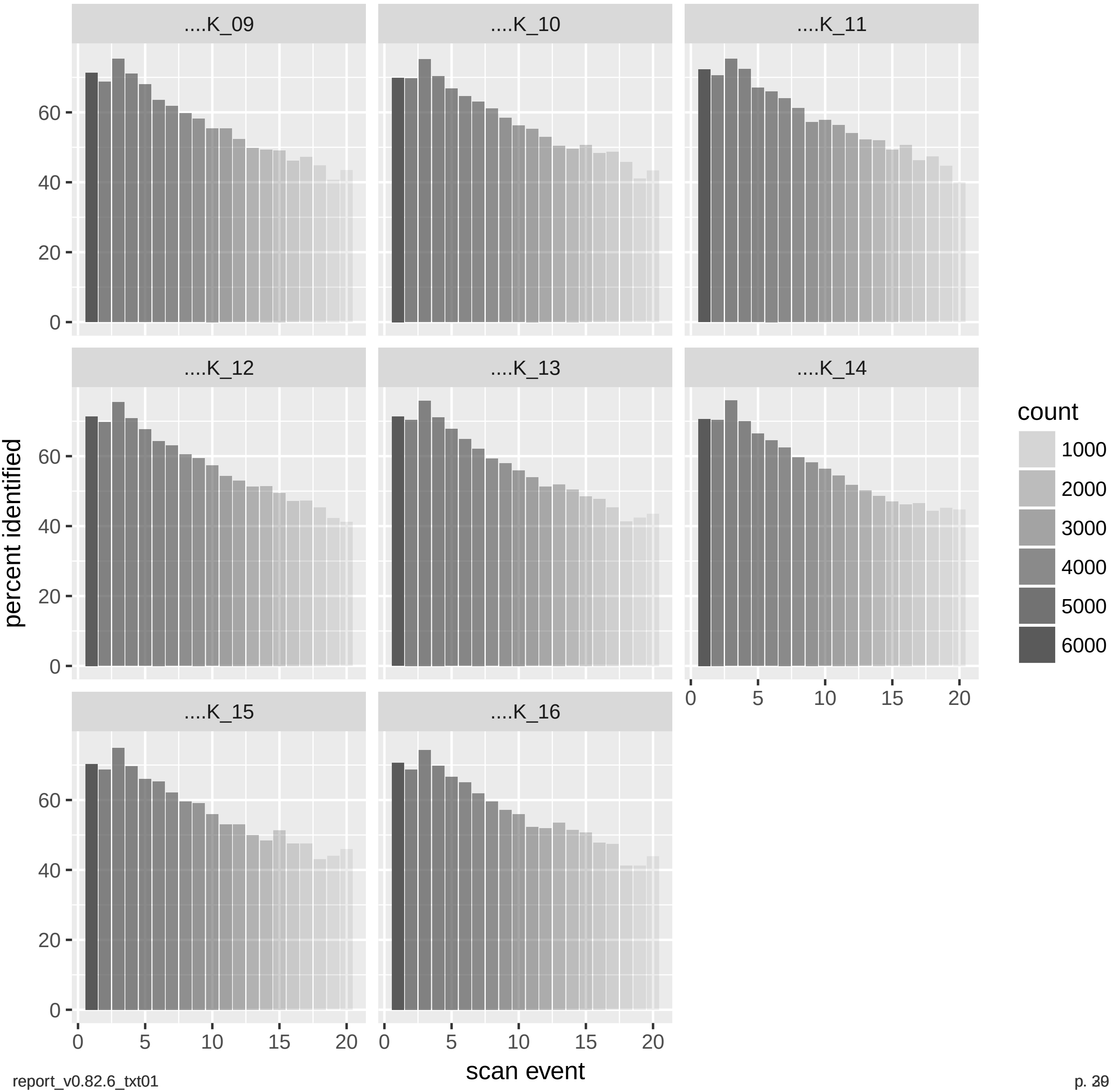

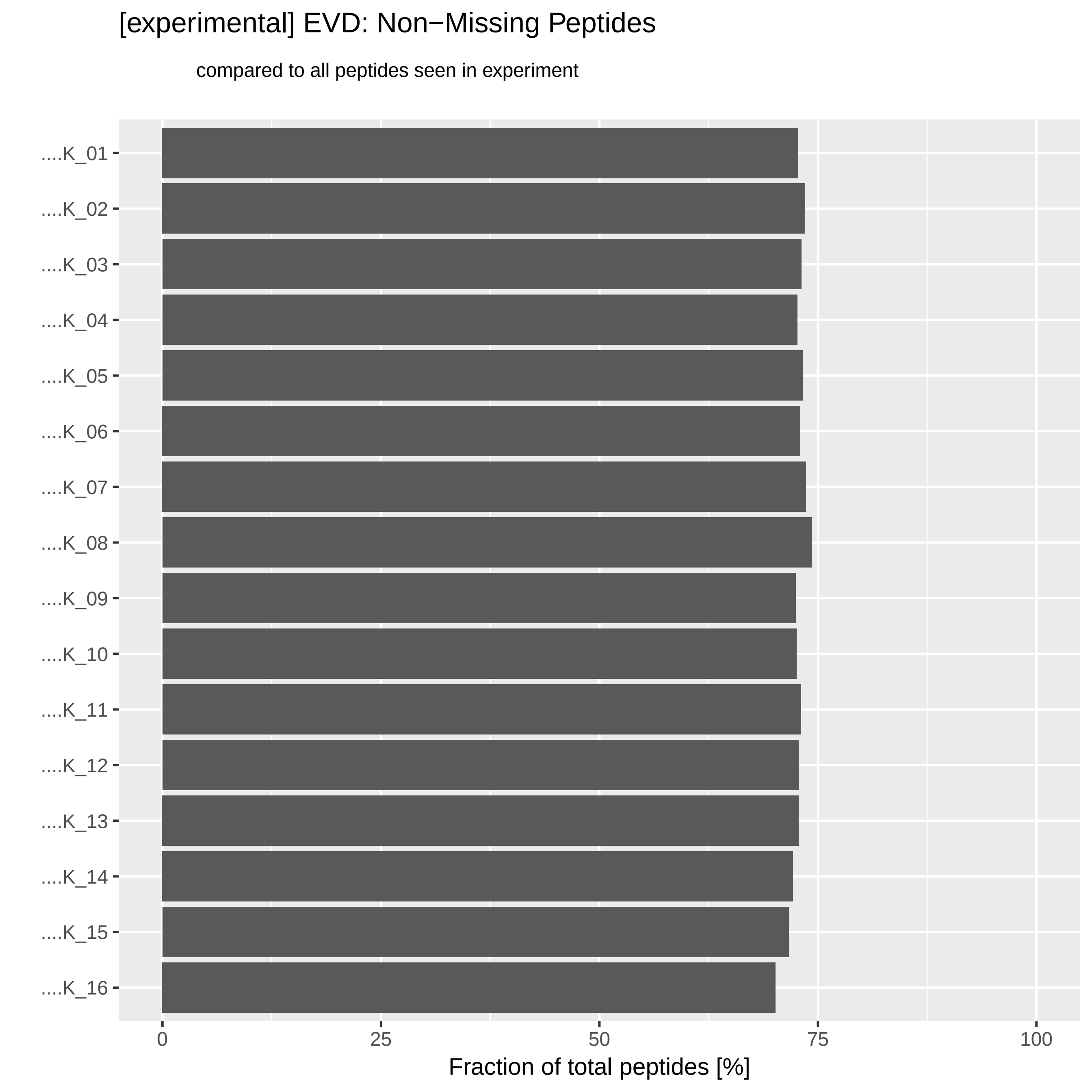
[experimental] EVD: Non−Missing Peptides
compared to all peptides seen in experiment
....K_01
....K_02
....K_03
....K_04
....K_05
....K_06
....K_07
....K_08
....K_09
....K_10
....K_11
....K_12
....K_13
....K_14
....K_15
....K_16
0
25
50
75
100
Fraction of total peptides [%]

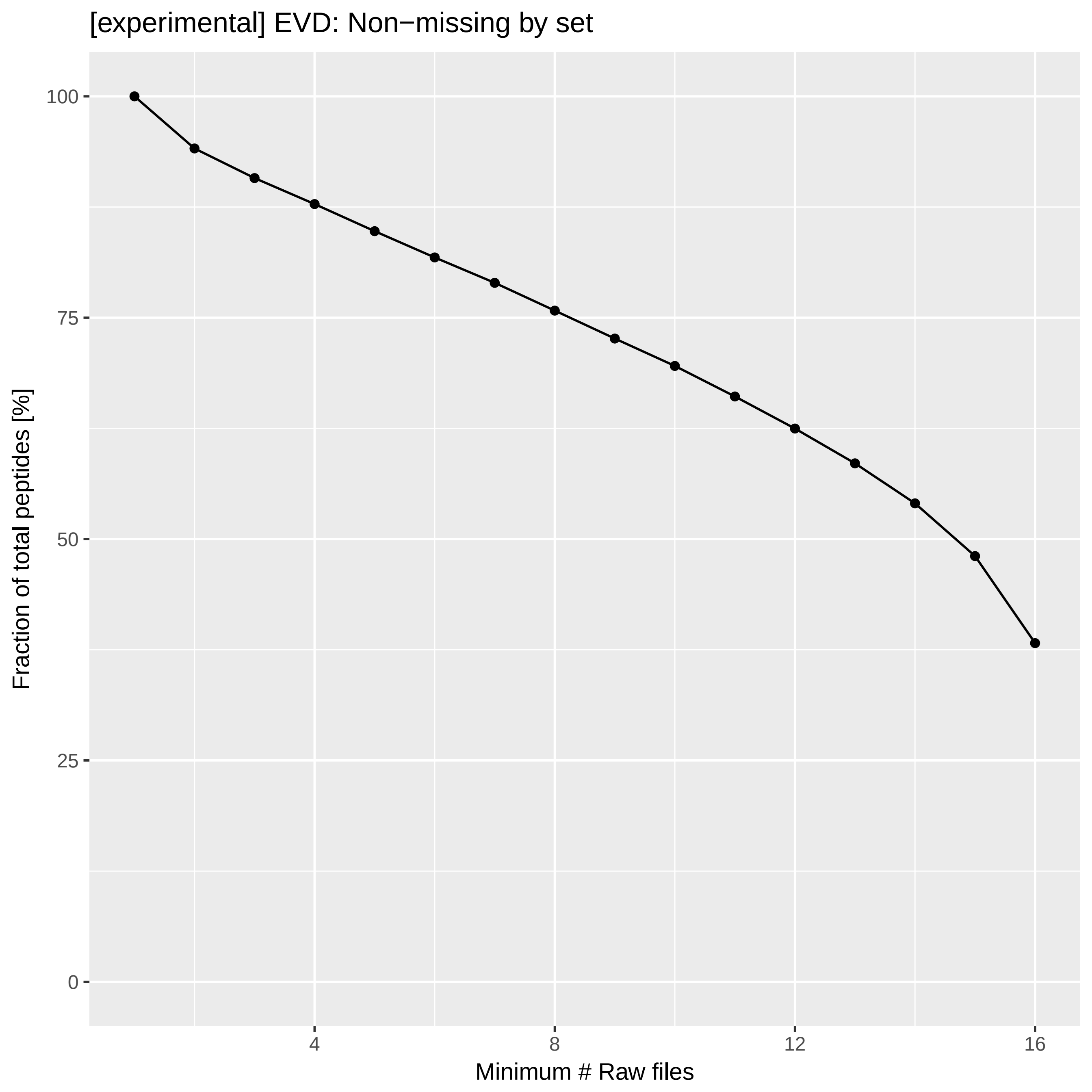
[experimental] EVD: Non−missing by set
Fraction of total peptides [%]
100
75
50
25
0
4
8
12
16
Minimum # Raw files

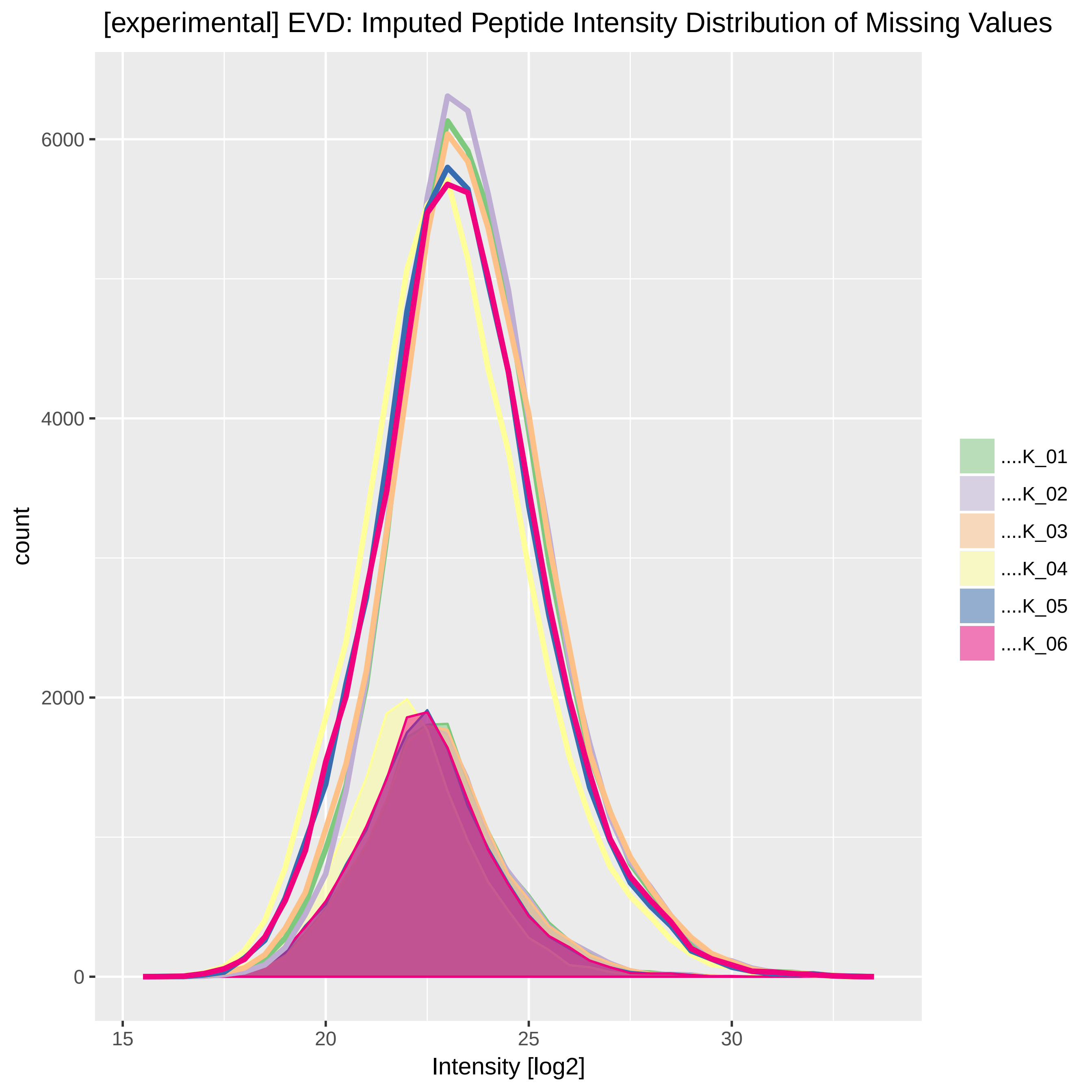
[experimental] EVD: Imputed Peptide Intensity Distribution of Missing Values
count
6000
4000
2000
0
15
20
25
30
Intensity [log2]
....K_01
....K_02
....K_03
....K_04
....K_05
....K_06

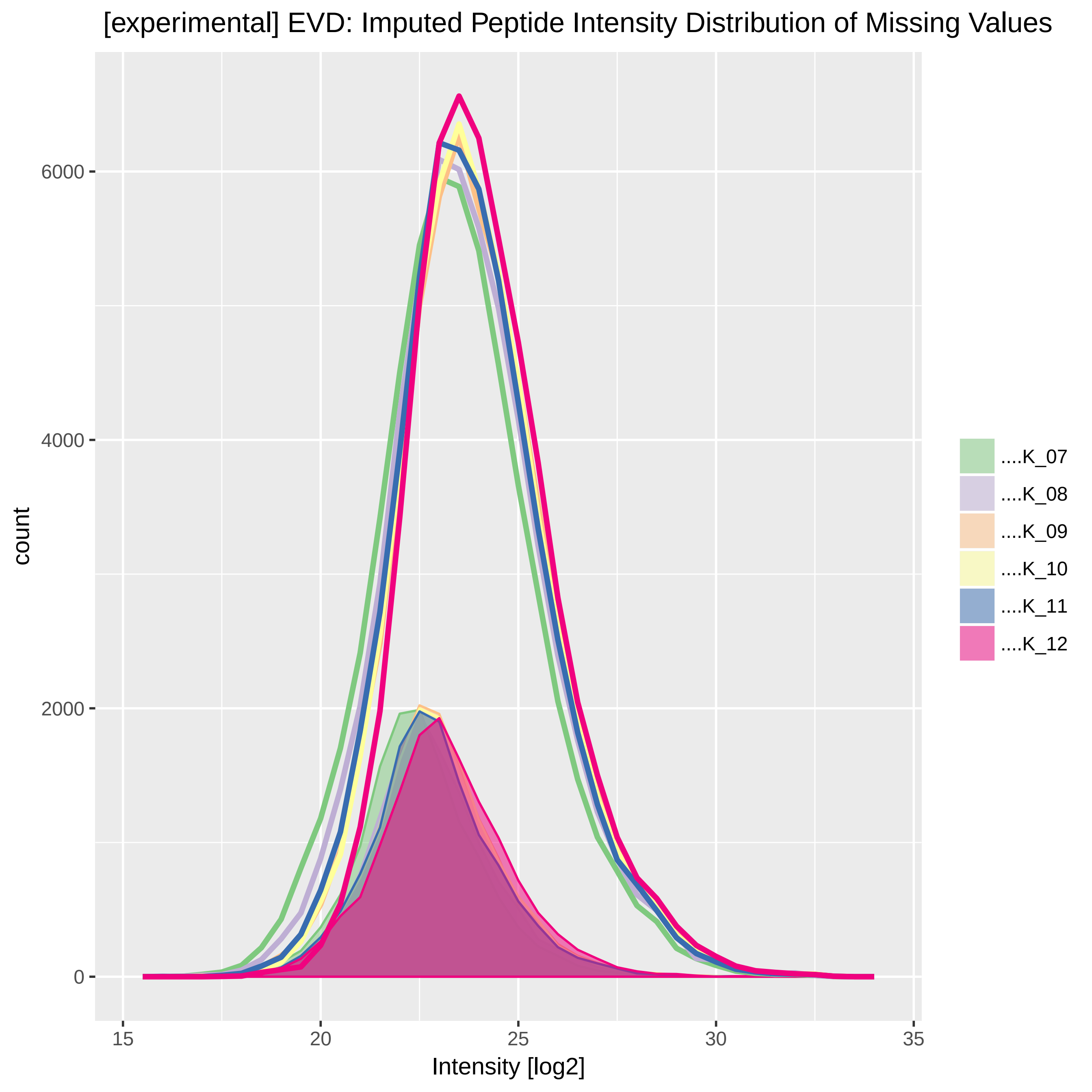

[experimental] EVD: Imputed Peptide Intensity Distribution of Missing Values
count
6000
4000
2000
0
15
20
25
30
35
Intensity [log2]
....K_07
....K_08
....K_09
....K_10
....K_11
....K_12

[experimental] EVD: Imputed Peptide Intensity Distribution of Missing Values

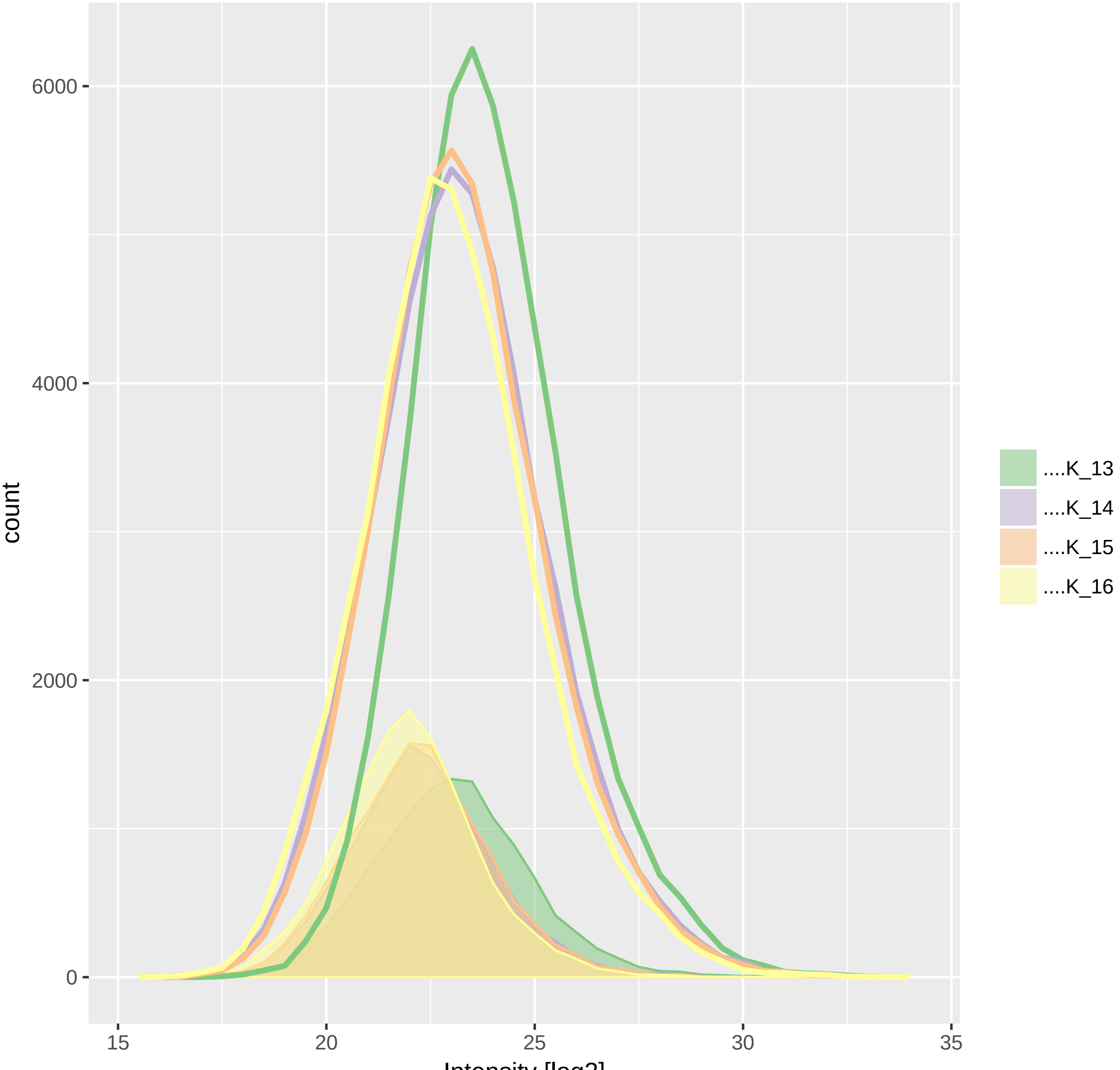

EVD: Peptide ID count

MBR gain: +42%

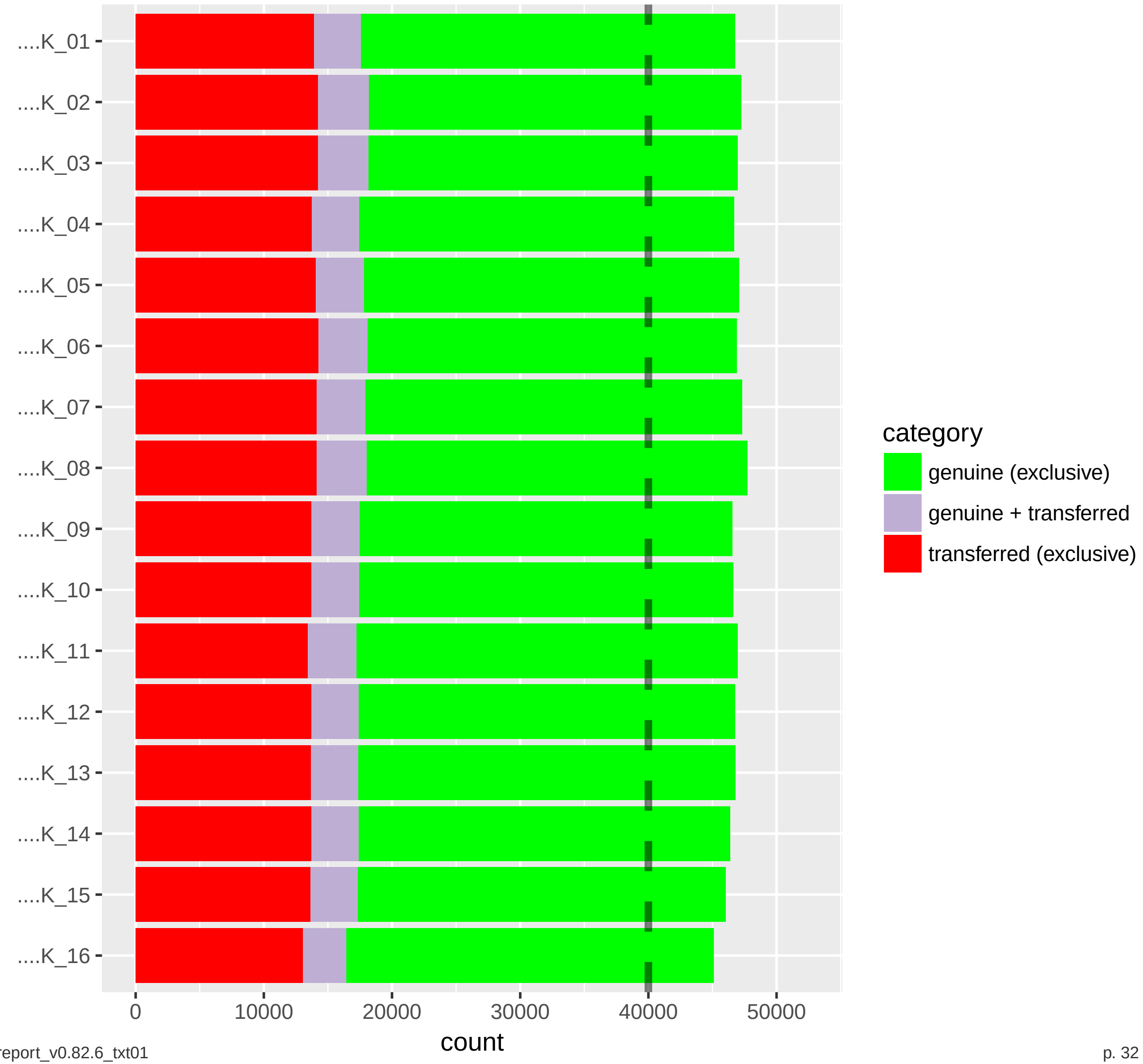

EVD: ProteinGroups count

MBR gain: +12%

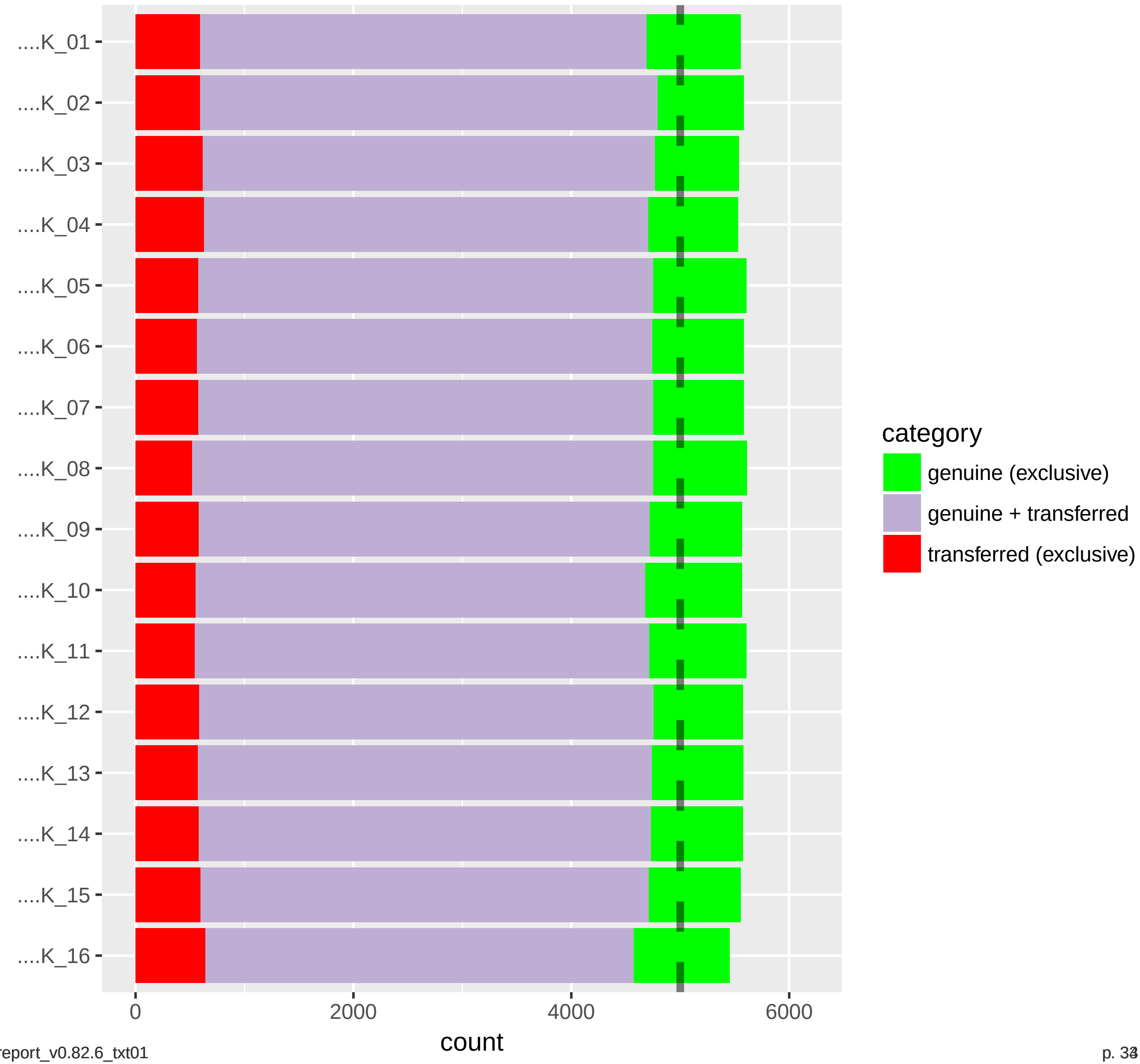

Supplement: S4 File — (PDF) [file pone.0213469.s010.pdf]
